# Marinopyrrole Derivatives as Potential Antibiotic Agents against Methicillin-Resistant *Staphylococcus aureus* (II)

**DOI:** 10.3390/md11082927

**Published:** 2013-08-15

**Authors:** Chunwei Cheng, Yan Liu, Hao Song, Lili Pan, Jerry Li, Yong Qin, Rongshi Li

**Affiliations:** 1 Key Laboratory of Drug Targeting and Drug Delivery Systems of the Ministry of Education and State Key Laboratory of Biotherapy, Department of Medicinal Natural Products, West China School of Pharmacy, Sichuan University, Chengdu 610041, China; E-Mails: chengchunwei666@163.com (C.C.); haoright@163.com (H.S.); pande179@163.com (L.P.); 2 Chemical Biology & Molecular Medicine Program, Department of Drug Discovery, H. Lee Moffitt Cancer Center and Research Institute, 12902 Magnolia Drive, Tampa, FL 33612, USA; E-Mails: yan.liu@moffitt.org (Y.L.); jerry.li.91@berkeley.edu (J.L.); 3 The Innovative Drug Research Centre, Chongqing University, Chongqing 400000, China; 4 Department of Oncologic Sciences, Morsani College of Medicine, University of South Florida, 12901 Bruce B. Downs, Tampa, FL 33612, USA

**Keywords:** antibiotics, marinopyrroles, MRSA, MRSE, MSSA, SAR

## Abstract

Methicillin-resistant *Staphylococcus aureus* (MRSA) continues to be a major problem, causing severe and intractable infections worldwide. MRSA is resistant to all beta-lactam antibiotics, and alternative treatments are limited. A very limited number of new antibiotics have been discovered over the last half-century, novel agents for the treatment of MRSA infections are urgently needed. Marinopyrrole A was reported to show antibiotic activity against MRSA in 2008. After we reported the first total synthesis of (±)-marinopyrrole A, we designed and synthesized a series of marinopyrrole derivatives. Our structure activity relationship (SAR) studies of these novel derivatives against a panel of Gram-positive pathogens in antibacterial assays have revealed that a *para-*trifluoromethyl analog (**33**) of marinopyrrole A is ≥63-, 8-, and 4-fold more potent than vancomycin against methicillin-resistant *Staphylococcus* *epidermidis* (MRSE), methicillin-susceptible *Staphylococcus aureus* (MSSA) and MRSA, respectively. The results provide valuable information in the search for new-generation antibiotics.

## 1. Introduction

The global crisis of antibiotic resistance has spread rapidly over the past several decades. Methicillin-resistant *Staphylococcus aureus* (MRSA) infections have reached epidemic proportions in many countries [1] and represent the most common cause of skin and soft tissue infections in the United States [2]. Both hospital-associated and community-associated MRSA can exhibit broad resistance to multiple classes of antibiotics [1,2,3,4,5]. Hospital-associated MRSA infections are common among healthcare facilities and are resistant to many antibiotics. However, community-associated MRSA strains are highly virulent and even infect healthy individuals; the incidence of these infections has skyrocketed in the past decade. The relative abandonment of antibiotic discovery and development by the pharmaceutical industry has opened opportunities for academic researchers to discover new antibiotics to treat these increasingly problematic infections. Except for the addition of the oxazolidinone linezolid [6] in 2000, the lipopeptide daptomycin [7] in 2003, and the US Food and Drug Administration’s recent approval of ceftaroline [8], a very limited number of new antibiotics have been marketed over the past half-century. Only two new classes of anti-MRSA drugs have been approved in the last 40 years. Clearly, novel agents for the treatment of MRSA infections are urgently needed [9]. 

Marinopyrroles were first reported to show antibiotic activity against MRSA in 2008 by the Fenical group [10]. Due to their novel class of molecular structures and promising biological properties, marinopyrroles have attracted considerable attention [11,12,13,14,15,16,17,18,19]. We reported the first total synthesis of (±)-marinopyrrole A, along with 12 derivatives in early 2010 [12]. Synthesis of (±)-marinopyrrole A via an intermolecular Ullman coupling reaction as a key step to form bispyrrole system was published by Kanakis and Sarli five months later [13]. In 2011, the Nicolaou group published a new five-step method to access marinopyrrole derivatives, (+)- and (−)-atropisomer after a chiral separation of (±)-marinopyrrole A using HPLC, as well as their antibiotic activities against MRSA [14]. Recently, the Moore group published biosynthesis of marinopyrrole A via an *N*,*C*-bipyrrole homocoupling catalyzed by two flavin-dependent halogenases [17]. Most recently, racemic marinopyrrole B was reported by total synthesis from the Clive group [18]. During the preparation of this manuscript, a review of the marinopyrroles appeared [19]. We have reported synthesis of a novel series of “non-symmetrical” marinopyrrole derivatives and their antibiotic activities [15]. These derivatives demonstrated superior antibiotic activities to that of the parent marinopyrrole A against MRSA [15]. Last year, we published optimization of the key step to avoid the formation of oxazepine byproduct [16].

## 2. Results and Discussion

### 2.1. Synthesis of Marinopyrrole Derivatives

**Scheme 1:**
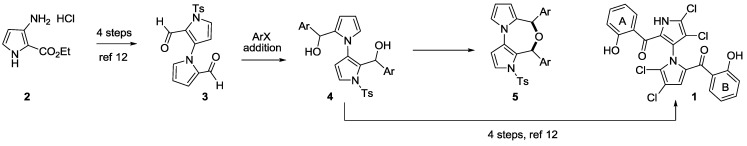
Synthesis of marinopyrrole A.

**Scheme 2:**
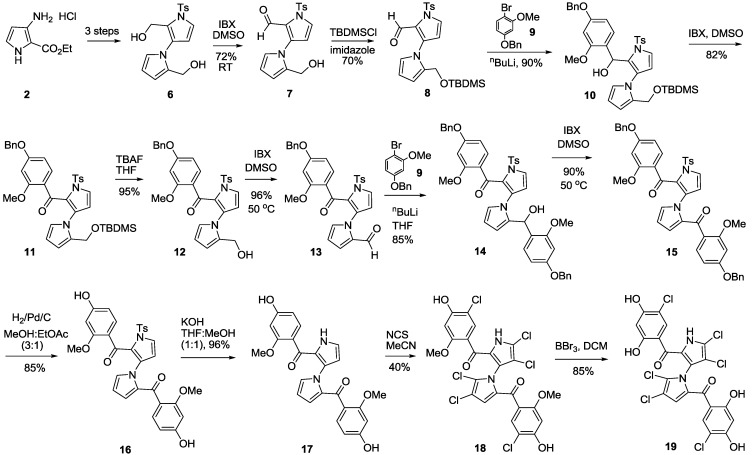
General route for the synthesis of marinopyrrole derivatives **18** and **19**.

**Figure 1:**
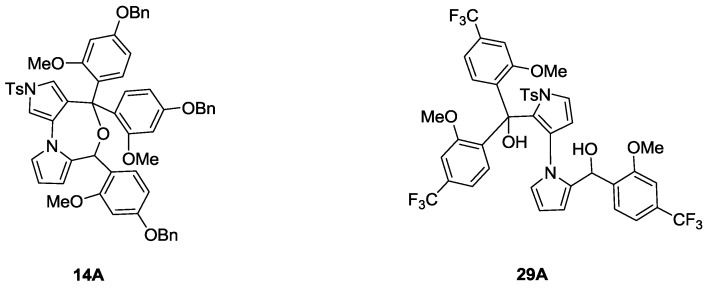
Dialkylation byproduct.

**Scheme 3:**
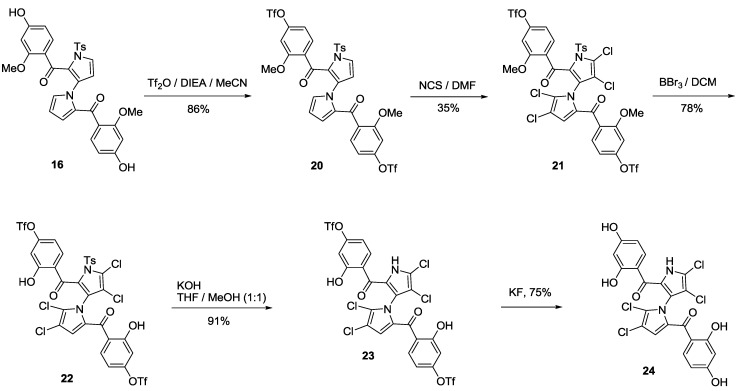
Synthesis of marinopyrrole derivative **24**.

In 2010, we reported the first total synthesis of (±)-marinopyrrole A (**1**) and a dozen “symmetrical” derivatives [12], which bear the same substituents with the same substitution patterns on both rings A and B attached to the carbonyl groups as shown in Scheme 1. A nine-step synthesis was required to access **1**, in which a limitation was the formation of oxazepine byproduct **5**. Recently, we optimized the synthesis of marinopyrrole derivatives [16] to circumvent the chemistry issues that we reported in our first publication [12]. As shown in Scheme 1, the formation of oxazepine **5** after a Grignard addition to aldehyde **3**, followed by work-up under weakly acidic conditions (AcOH) cannot be avoided due to reactive diol intermediate **4**. Oxazepine **5** is readily formed even on silica gel with attempted purification by column chromatography. Although this issue was avoided by direct oxidation of the crude diol **4** to ketone [12], the reproducibility suffered and the yields varied from batch to batch. The key to solving such chemistry issues was sequential introduction of rings A and B via mono-protection of aldehyde **3**, as we reported earlier [16]. In this paper, we report alternative approaches to accomplishing the sequential introduction of rings A and B. As shown in Scheme 2, selective oxidation of diol **6** was achieved by IBX in DMSO in 72% yield. Protection of **7** with TBDMS afforded intermediate **8** in 70% yield. Addition of **9** to aldehyde **8** in 90% yield followed by oxidation of the resulting alcohol **10** by IBX in DMSO afforded ketone **11** in 82% yield. Compound **12** was obtained by removal of the silyl-protecting group in **11** by TBAF in 95% yield. Oxidation of **12** by IBX in DMSO furnished the aldehyde 13 in 96% yield. Addition of **9** to **13** furnished **14** in 85% yield, which was subjected to oxidation by IBX in DMSO affording diketone **15** in 90% yield. Double alkylation on both aldehyde and ketone in **13** was observed, generating both desired product **14** in 85% and oxazepine byproduct **14A** in 5% yield (Figure 1 and Experimental Section). It is worthwhile to note that this new synthetic strategy is versatile. This synthetic route has paved the way to access not only the “symmetrical” marinopyrroles but also “non-symmetrical” congeners when two different Grignard or organic lithium reagents are used. Deprotection of diketone **15** by hydrogenolysis furnished **16**. Removal of tosyl protecting group by KOH generated **17**, which was then converted to **18** by chlorination with NCS. The final symmetrical marinopyrrole derivative **19** was obtained after demethylation using BBr_3_/DCM [12]. With intermediate **16** as a starting material (Scheme 3), the two phenolic hydroxyl groups were activated by trifluoromethanesulfonic anhydride in the presence of DIEA in anhydrous acetonitrile. Tetrachlorination of **20** with NCS in DMF furnished **21** in 35% yield. Demethylation of **21** with BBr_3_ followed by removal of the tosyl-protecting group afforded an intermediate **23**. Compound **24** was obtained after removal of Tf with KF in 75% yield. 

**Scheme 4:**
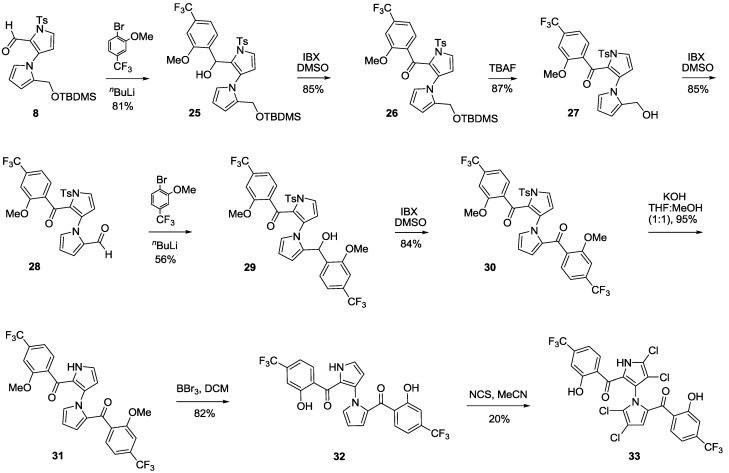
Synthesis of marinopyrrole derivative **33**.

A nine-step sequence to access symmetrical marinopyrrole derivative **33** is shown in Scheme 4. Similar to the synthetic route towards compound **19** in Scheme 2, (2-methoxy-4-(trifluoromethyl)phenyl)lithium was added to aldehyde **8** followed by oxidation of **25** by IBX in DMSO to afford **26**. Second addition of (2-methoxy-4-(trifluoromethyl)phenyl)lithium to aldehyde **28**, generated by removal of TBDMS in **26** with TBAF followed by IBX oxidation of **27**, furnished **29**. As with the synthesis of compound **14** in Scheme 2, double alkylation on both aldehyde and ketone in **28** occurred, furnishing both **29** as a desired product in 56% yield and diol byproduct **29A** in 15% yield (Figure 1 and Experimental Section). However, no oxazepine byproduct was observed, presumably due to stabilization of precursor **29A** by electron withdrawing CF_3_ groups in the *para-*position of phenyl rings. Diketone **30** was obtained after oxidation of **29** with IBX in DMSO in 84% yield. Removal of the tosyl group in **30** afforded **31**, which was then subjected to demethylation using BBr_3_ in DCM in 82% yield. The final compound **33** was obtained after tetrachlorination of **32** with NCS in MeCN.

### 2.2. SAR Studies

**Figure 2:**
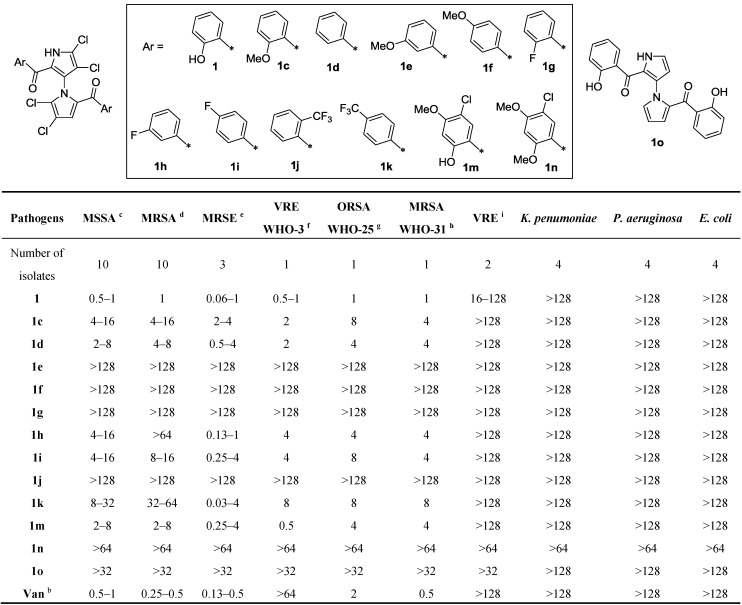
*In vitro* activity of marinopyrrole A and an initial set of derivatives ^a^.

^a^ MIC in μg/mL, synthesis of compounds **1-10** was reported previously [12], except for those marked with WHO, all pathogens were isolated between July 2008 and October 2009 in hospitals located in Beijing, Guangzhou, Sichuan, Shandong and Jiangsu Province; ^b^ Vancomycin; ^c^ Methicillin-susceptible *Staphylococcus aureus*; ^d^ Methicillin-resistant *Staphylococcus aureus*; ^e^ Methicillin-resistant *Staphylococcus epidermidis*; ^f^ Moderately vancomycin-resistant *Enterococcus faecium* with the *VanA* gene; ^g^ Oxacillin-resistant *Staphylococcus aureus*; ^h^ Methicillin-resistant *Staphylococcus aureus* with the *mecA* gene and *PBP2a*; ^i^ Highly vancomycin-resistant *Enterococcus faecalis*.

After we achieved the first total synthesis of (±)-marinopyrrole A (**1**) and 12 derivatives [12], their activity against seven groups of Gram-positive and three Gram-negative pathogens were evaluated. The results are reported here. Vancomycin was used as a positive control in all experiments. The potency of the compound was determined and expressed as minimum inhibitory concentration (MIC). As shown in Figure 2, the parent compound **1** demonstrated antibiotic activity comparable to that of vancomycin. The de-halogenated precursor **10**, which lacks the tetrachloro substituents on the bispyrrole, exhibited >64 fold lower activity (MIC > 32 μg/mL) against the pathogens tested. This result indicates that four chloro atoms on the pyrrole rings play an important role for antibiotic activity. The hydroxyl group in the *ortho-*position of the phenyl ring (**1**) is critical. Replacement of the hydroxyl group with other substituents such as H, F, OMe, or CF_3_ led to lower activity or to a complete loss of activity (cf., **1c**, **1d**, **1g**, **1j**, and **1n**, Figure 2). Besides forming an internal hydrogen bond with the ketone moiety, as described for marinopyrrole B (3′-Br analogue of **1**) [10], the hydroxyl group in **1** probably serves similarly as a hydrogen bond donor rather than a hydrogen bond acceptor when binding to the targets. This observation was supported by the fact that compounds **1c** and **1n**, which possess a hydrogen bond acceptor, showed significant loss of activity. An electron-withdrawing group seems to be tolerated in the *meta-* and *para-*positions of the phenyl ring and the size of the substituent is less important (cf., **1d**–**1i**, and **1k**). Compound **1k**, which bears the strong electron-withdrawing group CF_3_ in the *para-*position and lacks the key hydroxyl group in the *ortho-*position, exhibited the most potent activity against MRSE compared with the parent compound **1**, vancomycin, and other derivatives. Although an electron-donating group in the *para-*position may reduce potency, compound **1m** (bearing a hydroxyl group in the *ortho-*position) also showed activities against the tested pathogens MSSA, MRSA, ORSA (oxacillin-resistant *Staphylococcus* *aureus*), MRSE, and VRE (vancomycin-resistant *Enterococcus faecalis*) comparable to **1**. This result reinforces further that the hydroxyl group in the *ortho-*position of the phenyl ring is pivotal. All compounds are inactive against Gram-negative pathogens *K. pneumoniae*, *P. aeruginosa*,and *E. coli*. With the preliminary SAR data acquired from the initial set of marinopyrrole derivatives in hand as shown in Figure 2, design of several symmetrical marinopyrrole congeners was focused on the substitutions in both *ortho-* and *para-*positions of the phenyl rings. Keeping the *ortho*-hydroxyl and *meta-*chloro groups in each ring constant, the *para-*methoxy was replaced by hydroxyl, furnishing compound **19** (Scheme 2) to see whether hydrogen bond donors or hydrogen bond acceptors in the *para-*position are preferred (comparing the activity with compound **1m**). Compound **24**, bearing two hydroxyl groups in both *ortho-* and *para-*positions, was designed and synthesized (Scheme 3) to probe if hydrogen bond donors are favored in both *ortho-* and *para-*positions. Compound **33** was designed and synthesized (Scheme 4) based on the SAR information obtained from compound **1** and **1k**. As shown in Figure 2, compound **1k** demonstrated anti-MRSE activity in the range similar to that of **1**, but with greater potency than that of vancomycin. However, **1k** showed much lower activity against the other pathogens.

### 2.3. *In Vitro* Antibacterial Activity

**Figure 3:**
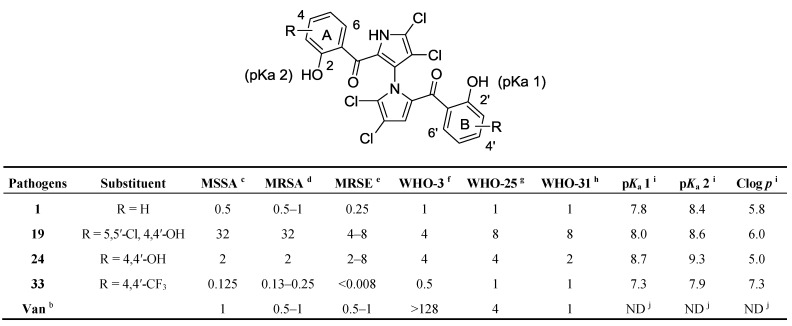
*In vitro* activity and physicochemical properties of marinopyrrole A andnew derivatives ^a^.

^a^ MIC in μg/mL, except for those marked with WHO, all pathogens were isolated in recent 2–3 years from hospitals located in Beijing, Guangzhou, Sichuan, Shandong and Jiangsu Province; ^b^ Vancomycin; ^c^ Methicillin-susceptible *Staphylococcus aureus*, data from **8** isolates;^d^ Methicillin-resistant *Staphylococcus aureus*,data from **8** isolates; ^e^ Methicillin-resistant *Staphylococcus epidermidis*, data from **4** isolates; ^f^ Moderately vancomycin-resistant *Enterococcus faecium* with the *VanA* gene; ^g^ Oxacillin-resistant *Staphylococcus aureus*; ^h^ Methicillin-resistant *Staphylococcus aureus* with the *mecA* gene and *PBP2a*; ^i^ Calculated using ChemAxon Software Version 5.12.3; ^j^ Not determined.

A preliminary evaluation of **1** and 12 marinopyrrole derivatives showed that they were active in various magnitudes against seven groups of Gram-positive pathogens and inactive against three Gram-negative pathogens (Figure 2). The parent compound **1** exhibited potency similar to that of vancomycin against the clinical isolates of methicillin-susceptible *Staphylococcus aureus* (MSSA), methicillin-resistant *Staphylococcus aureus* (MRSA),methicillin-resistant *Staphylococcus epidermidis* (MRSE), and non-clinical isolates of MRSA (WHO-31) and oxacillin-resistant *Staphylococcus aureus* (ORSA, WHO-25). Design and synthesis of marinopyrrole A derivatives based on the initial results of SAR studies led to novel derivatives **19**, **24**, and **33** using our recently optimized chemistries (*vide supra*). Among these derivatives as shown in Figure 3, compound **19** showed 4–16 fold decreased potency against MRSA and 2–16 fold against MRSE, respectively, when compared with compound **1m**. The only structural difference is the *para-*hydroxyl (**19**) and *para-*methoxy (**1m**) substitution on the phenyl groups. Compound **24**, bearing the same *para-*hydroxyl but lacking *meta-*chloro when compared with **19**, exhibited similar potency to that of **1m**. Compound **33** bearing both a strong electron-withdrawing group (CF_3_) in the *para-*position and an *ortho-*hydroxyl group exhibited the most potent antibacterial activity. As shown in Figure 3, compound **33** is eight-fold and four-fold more potent than vancomycin and **1** against MSSA, respectively. Compound **33** is also 2–4 fold more potent than both vancomycin and **1** against MRSA. Most significantly, compound **33** with MIC of 8 ng/mL is >62 fold and >31 fold more potent than vancomycin and **1** against MRSE, respectively. With respect to the antibacterial activities of **33** and **1k**, which lack the *ortho-*hydroxyl group, compound **33** showed at least 128, 64, and 4 fold more potent than **1k** against MRSA, MSSA, and MRSE, respectively. This result further reinforces that the perihydroxyl group is crucial. The effect of the electron withdrawing CF_3_ group in **33** on the p*K*_a_ of the phenols and lipophilicity may play an essential role in activity.

### 2.4. Physicochemical Properties of the Marinopyrroles

Both p*K*_a_ and log *p* values were calculated using ChemAxon Software Version 5.12.3 [20,21]. The p*K*_a_ values of marinopyrrole A (**1**) are predicted to be 7.8 (p*K*_a_ 1) and 8.4 (p*K*_a_ 2), respectively (Figure 3). The difference in p*K*_a_ values for the hydroxyl group in ring A and ring B is presumably due to the axially chiral environment. The p*K*_a_ values of **1** are 1.6–2.2 log units lower than that of phenol (p*K*_a_ = 9.98 [22]). Equilibrium may exist between open conformations and closed conformations in **1**, similar to those in a recent report of intramolecular hydrogen bonding in medicinal chemistry [23]. In the open forms, both perihydroxyl and carbonyl groups are available for hydrogen bond interactions as a donor and an acceptor, respectively. Both polar groups in the open forms can interact with solvents, intermolecular molecules, or protein targets. While in the closed forms, both polar groups form a stable six-membered ring system via internal hydrogen bonds resulting in more lipophilic molecules. The Fenical group reported the X-ray structure of marinopyrrole B (3′-Br analog of **1**) that confirmed the intramolecular hydrogen bonds between the perihydroxyl and the carbonyl group [10]. These intramolecular hydrogen bond interactions contribute to not only lowering the p*K*_a_ values but also increasing its lipophilicity [23]. The calculated log *p* value of **1** is 5.8, which marginally violates the rule of five (RO5), drug-like properties formulated by Lipinski [24]. The calculated log *p* values of **24** obeyed the RO5, presumably due to the presence of more hydrophilic hydroxyl group. The p*K*_a_ and Clog *p* values of **33** are predicted to be 7.3–7.9 and 7.3, 0.5 log units lower in p*K*_a_ and 1.5 log units higher in Clog *p* than those of **1**, respectively. These changes are due to additional electron-withdrawing CF_3_ group. Although the marinopyrroles reported in this paper are most likely in the closed forms like marinopyrrole B reported [10], it is possible for them to adopt the open forms if stronger hydrogen bonds between the marinopyrroles and the protein target predominate. In this case, the internal hydrogen bonds of marinopyrroles are weakened and the resulting molecules are expected to have lower log *p* values. Notwithstanding that there are no direct correlations between the p*K*_a_, Clog *p* and *in vitro* antibiotic activity of the marinopyrroles discussed above, it is important to understand physicochemical properties when their SARs are optimized.

## 3. Experimental Section

### 3.1. Synthesis of Marinopyrrole Derivatives

All chemicals were purchased from commercial suppliers and used without further purification. All solvents were dried and distilled before use. Tetrahydrofuran was distilled from sodium/benzophenone. Dichloromethane and acetonitrile were distilled over calcium hydride. Flash column chromatography was performed with silica gel (200–300 mesh). ^1^H NMR spectra were recorded at either 400 MHz or 600 MHz at ambient temperature. ^13^C NMR spectra were recorded at either 100 or 150 MHz at ambient temperature. Infrared spectra were recorded on a spectrophotometer (Perkin-Elmer Spectrum 100). Copies of NMR spectra of all the described compounds are provided in a Supporting Information Document. Melting points were determined with melting point apparatus (Fukai X-4). High resolution mass spectra were performed by electrospray ionization (ESI) on an Agilent ESI-TOF LC-MS 6200 system. Analytical HPLC was performed on an Agilent 1100 series with diode array detectors and auto samplers. All tested compounds possessed a purity of not less than 95%. 

2-(Hydroxymethyl)-1′-tosyl-1′*H*-1,3′-bipyrrole-2′-carbaldehyde (**7**). To a solution of (1′-tosyl-1′*H*-1,3′-bipyrrole-2,2′-diyl)dimethanol **6** obtained from ethyl 3-amino-1*H*-pyrrole-2-carboxylate hydrochloride **2** in three steps [3] (2.00 g, 5.8 mmol) in DMSO (30 mL) was added IBX (1.78 g, 6.4 mmol) at room temperature. After being stirred for 5 h, the mixture was quenched with water (50 mL). The suspension was filtered and the filtrate was extracted with EtOAc (25 mL × 3). The combined organic layers were dried over anhydrous sodium sulfate, filtered and concentrated in vacuum. The residue was purified by flash column chromatography (25% EtOAc/petroleum ether, *R*_f_ = 0.2) to give **7** (1.43 g, 72%) as a liquid. ^1^H NMR (400 MHz, CDCl_3_) δ 2.43 (s, 3H), 2.87 (br, s, 1H), 4.44 (s, 2H), 6.33 (d, *J* = 3.2 Hz, 1H), 6.38 (dd, *J* = 4.0, 2.8 Hz, 1H), 7.05 (s, 1H), 7.09 (dd, *J* = 4.4, 2.0 Hz, 1H), 7.31 (d, *J* = 3.6 Hz, 1H), 7.35 (d, *J* = 8.4 Hz, 2H), 7.82 (d, *J* = 8.4 Hz, 2H), 9.46 (s, 1H) ppm; ^13^C NMR (CDCl_3_, 100 MHz) δ 21.70, 53.00, 111.00, 111.16, 121.55, 123.57, 127.11, 127.11, 127.32, 128.70, 130.24, 130.24, 132.90, 132.99, 135.55, 145.77, 179.27 ppm; HRMS (M + Na^+^) calcd. for C_17_H_16_N_2_NaO_4_S 367.0728, found 367.0733; IR (KBr) 3425, 3118, 2924, 2875, 2730, 1662, 1595, 1369, 1317, 1177, 1086, 1012, 774, 754 and 669 cm^−1^.

2-((*tert*-Butyldimethylsilyloxy)methyl)-1′-tosyl-1′*H*-1,3′-bipyrrole-2′-carbaldehyde (**8**). To a solution of **7** (6.00 g, 17.4 mmol) in dry CH_2_Cl_2_ (60 mL) was added imidazole (2.37 g, 34.8 mmol) at room temperature. After being stirred for 5 min, TBDMSCl (5.30 g, 34.8 mmol) was added. The mixture was stirred for about 2.5 h and quenched with water (50 mL) and extracted with CH_2_Cl_2_ (25 mL × 3). The combined organic layers were dried over anhydrous sodium sulfate, filtered and concentrated in vacuum. The residue was purified by flash column chromatography (20% EtOAc/petroleum ether, *R*_f_ = 0.35) to give **8** (5.59 g, 70%) as a liquid. ^1^H NMR (400 MHz, CDCl_3_) δ −0.04 (s, 6H), 0.82 (s, 9H), 2.42 (s, 3H), 4.61 (s, 2H), 6.34–6.36 (m, 2H), 7.03 (t, *J* = 1.6 Hz, 1H), 7.11 (dd, *J* = 4.0, 1.6 Hz, 1H), 7.26 (d, *J* = 2.4 Hz, 1H), 7.29 (d, *J* = 8.4 Hz, 2H), 7.85 (d, *J* = 8.4 Hz, 2H), 9.46 (s, 1H) ppm; ^13^C NMR (CDCl_3_, 100 MHz) δ −5.67, −5.67, 18.44, 21.65, 25.88, 25.88, 25.88, 53.34, 110.65, 111.12, 121.08, 121.73, 127.09, 127.09, 127.46, 128.65, 129.90, 129.90, 132.11, 133.25, 136.34, 145.19, 179.04 ppm; HRMS (M + Na^+^) calcd. for C_23_H_30_N_2_NaO_4_SSi 481.1593, found 481.1591; IR (KBr) 3444, 3130, 2952, 2928, 2857, 2787, 1665, 1595, 1463, 1336, 1252, 1182, 1009, 841, 668, 604 cm^−1^.

(4-(Benzyloxy)-2-methoxyphenyl)(2-((*tert*-butyldimethylsilyloxy)methyl)-1′-tosyl-1′*H*-1,3′-bipyrrol-2′-yl)methanol (**10**). To a solution of 4-(benzyloxy)-1-bromo-2-methoxybenzene (**9**) (0.80 g, 2.7 mmol) in dry THF (5 mL) at −78 °C under N_2_ was slowly added *N-*BuLi (1.09 mL, 2.5 M in *N*-pentane, 2.7 mmol). After being stirred for 40 min, a solution of **8** (0.50 g, 1.1 mmol) in dry THF (1.5 mL) was added slowly via a syringe. The mixture was stirred for about 1 h and quenched by addition of a saturated aqueous NH_4_Cl (15 mL) solution and extracted with EtOAc (15 mL × 3). The combined organic layers were dried over anhydrous sodium sulfate, filtered and concentrated in vacuum. The residue was purified by flash column chromatography (15% EtOAc/petroleum ether, *R*_f_ = 0.2) to give **10** (0.66 g, 90%) as a pale yellow solid. mp 39.3–41.7 °C; ^1^H NMR (400 MHz, CDCl_3_) δ −0.03 (s, 3H), −0.02 (s, 3H), 0.84 (s, 9H), 2.40 (s, 3H), 2.73 (d, *J* = 5.2 Hz, 1H), 3.68 (s, 3H), 4.38 (d, *J* = 12.0 Hz, 1H), 4.63 (d, *J* = 11.6 Hz, 1H), 5.04 (s, 2H), 5.74 (d, *J* = 5.2 Hz, 1H), 6.02 (t, *J* = 2.0 Hz, 1H), 6.12 (t, *J* = 3.2 Hz, 1H), 6.28 (d, *J* = 3.6 Hz, 1H), 6.47–6.49 (m, 2H), 6.66 (t, *J* = 2.4 Hz, 1H), 7.14–7.17 (m, 2H), 7.28 (d, *J* = 10.4 Hz, 2H), 7.33–7.44 (m, 5H), 7.85 (d, *J* = 8.4 Hz, 2H) ppm; ^13^C NMR (CDCl_3_, 100 MHz) δ −5.70, −5.70, 18.44, 21.49, 25.87, 25.87, 25.87, 53.07, 55.21, 63.61, 69.97, 99.11, 104.80, 107.70, 107.96, 111.32, 121.26, 123.50, 123.74, 126.97, 126.97, 127.46, 127.46, 127.87, 128.01, 128.31, 128.31, 128.46, 129.21, 129.67, 129.67, 136.36, 136.40, 136.79, 144.84, 157.16, 159.22 ppm; HRMS (M + Na^+^) calcd. for C_37_H_44_N_2_NaO_6_SSi 695.2587, found 695.2598; IR (KBr) 3450, 3032, 2953, 2885, 2855, 1707, 1611, 1591, 1502, 1467, 1376, 1333, 1255, 1180, 1021, 838, 700, 600 cm^−1^.

(4-(Benzyloxy)-2-methoxyphenyl)(2-((*tert*-butyldimethylsilyloxy)methyl)-1′-tosyl-1′*H*-1,3′-bipyrrol-2′-yl)methanone (**11**). To a solution of **10** (3.67 g, 5.5 mmol) in dry DMSO (40 mL) was added IBX (3.06 g, 10.9 mmol) at room temperature. The mixture was allowed to warm up to 30 °C and stir for about 2 h. The mixture was quenched with water (60 mL) and extracted with EtOAc (25 mL × 3). The combined organic layers were dried over anhydrous sodium sulfate, filtered and concentrated in vacuum. The residue was purified by flash column chromatography (15% EtOAc/petroleum ether, *R*_f_ = 0.3) to give **11** (3.00 g, 82%) as a pale brown solid. mp 44.7–47.0 °C; ^1^H NMR (400 MHz, CDCl_3_) δ 0.004 (s, 3H), 0.01 (s, 3H), 0.86 (s, 9H), 2.40 (s, 3H), 3.73 (s, 3H), 4.67 (s, 2H), 5.10 (s, 2H), 6.21 (t, *J* = 3.6 Hz, 1H), 6.30 (d, *J* = 3.6 Hz, 1H), 6.52–6.54 (m, 2H), 6.69 (dd, *J* = 4.0, 1.6 Hz, 1H), 7.06 (s, 1H), 7.17 (d, *J* = 3.6 Hz, 1H), 7.26 (d, *J* = 7.2 Hz, 2H), 7.36–7.46 (m, 6H), 7.84 (d, *J* = 8.4 Hz, 2H) ppm; ^13^C NMR (CDCl_3_, 100 MHz) δ −5.53, −5.53, 18.50, 21.63, 25.96, 25.96, 25.96, 53.61, 55.65, 70.22, 99.68, 104.56, 108.92, 111.55, 121.34, 122.70, 127.03, 127.03, 127.17, 127.62, 127.62, 128.22, 128.69, 128.69, 129.79, 130.04, 130.04, 131.65, 131.65, 132.33, 132.90, 136.45, 136.80, 144.72, 159.28, 161.84, 183.51 ppm; HRMS (M + H^+^) calcd. for C_37_H_43_N_2_O_6_SSi 671.2611, found 671.2607; IR (KBr) 3737, 3144, 2953, 2929, 2855, 1639, 1603, 1499, 1413, 1375, 1274, 1179, 1158, 1026, 840 cm^−1^.

(4-(Benzyloxy)-2-methoxyphenyl)(2-(hydroxymethyl)-1′-tosyl-1′*H*-1,3′-bipyrrol-2′-yl)methanone (**12**). To a solution of **11** (2.92 g, 4.4 mmol) in dry THF (20 mL) was added TBAF (3.41 g, 13.1 mmol) at room temperature. After being stirred for about 5 h at room temperature, the mixture was quenched with water (25 mL) and extracted with EtOAc (15 mL × 3). The combined organic layers were dried over anhydrous sodium sulfate, filtered and concentrated in vacuum. The residue was purified by flash column chromatography (25% EtOAc/petroleum ether, *R*_f_ = 0.2) to give **12** (2.30 g, 95%) as a white solid. mp 55.7–58.3 °C; ^1^H NMR (400 MHz, CDCl_3_) δ 2.43 (s, 3H), 3.76 (s, 3H), 4.55 (s, 1H), 5.10 (s, 2H), 5.30 (s, 2H), 6.24 (dd, *J* = 4.0, 2.4 Hz, 1H), 6.31 (d, *J* = 3.6 Hz, 1H), 6.52–6.56 (m, 2H), 6.65 (dd, *J* = 4.0, 1.6 Hz, 1H), 7.00 (t, *J* = 2.4 Hz, 1H), 7.27 (d, *J* = 3.2 Hz, 1H), 7.30–7.45 (m, 8H), 7.84 (d, *J* = 8.4 Hz, 2H) ppm; ^13^C NMR (CDCl_3_, 100 MHz) δ 21.64, 53.17, 55.65, 70.18, 99.70, 104.57, 109.59, 110.96, 121.11, 122.58, 123.35, 127.20, 127.20, 127.55, 127.55, 128.20, 128.21, 128.66, 128.66, 128.90, 130.09, 130.09, 131.47, 132.47, 133.12, 135.81, 136.33, 145.34, 159.15, 161.85, 184.48 ppm; HRMS (M + H^+^) calcd. for C_31_H_29_N_2_O_6_S 557.1746, found 557.1743; IR (KBr) 3436, 2929, 2878, 1603, 1498, 1459, 1367, 1276, 1175, 1115, 1022, 867, 671 cm^−1^.

2′-(4-(Benzyloxy)-2-methoxybenzoyl)-1′-tosyl-1′*H*-1,3′-bipyrrole-2-carbaldehyde (**13**). To a solution of **12** (2.31 g, 4.2 mmol) in dry DMSO (30 mL) was added IBX (2.33 g, 8.3 mmol) at room temperature. The mixture was allowed to warm up to 50 °C and stir for about 3 h. The mixture was quenched with water (40 mL) and extracted with EtOAc (15 mL × 3). The combined organic layers were dried over anhydrous sodium sulfate, filtered and concentrated in vacuum. The residue was purified by flash column chromatography (20% EtOAc/petroleum ether, *R*_f _= 0.3) to give **13** (2.20 g, 96%) as a pale yellow solid. mp 132.7–135.0 °C; ^1^H NMR (400 MHz, CDCl_3_) δ 2.41 (s, 3H), 3.81 (s, 3H), 4.99 (s, 2H), 6.27–6.30 (m, 2H), 6.45 (d, *J* = 2.0 Hz, 1H), 6.49 (dd, *J* = 8.0, 2.0 Hz, 1H), 6.73 (dd, *J* = 3.6, 1.6 Hz, 1H), 6.98 (dd, *J* = 2.4, 1.6 Hz, 1H), 7.17–7.20 (m, 2H), 7.25–7.26 (m, 3H), 7.34 (d, *J* = 8.4 Hz, 2H), 7.38 (d, *J* = 8.4 Hz, 1H), 7.63 (d, *J* = 3.2 Hz, 1H), 7.88 (d, *J* = 8.4 Hz, 2H), 9.56 (s, 1H) ppm; ^13^C NMR (CDCl_3_, 100 MHz) δ 21.70, 55.65, 70.17, 99.64, 104.78, 110.13, 111.57, 121.95, 122.69, 125.22, 127.62, 127.62, 127.69, 128.04, 128.04, 128.21, 128.68, 128.68, 130.03, 130.03, 131.59, 132.02, 134.07, 134.78, 136.41, 139.25, 145.95, 159.37, 162.13, 177.08, 183.45 ppm; HRMS (M + H^+^) calcd. for C_31_H_27_N_2_O_6_S 555.1590, found 555.1600; IR (KBr) 3454, 3124, 3083, 2872, 2792, 1685, 1633, 1566, 1440, 1347, 1169, 1112, 1026, 763 cm^−1^.

(4-(Benzyloxy)-2-methoxyphenyl)(2-((4-(benzyloxy)-2-methoxyphenyl)(hydroxy)methyl)-1′-tosyl-1′*H*-1,3′-bipyrrol-2′-yl)methanone (**14**). To a solution of 4-(benzyloxy)-1-bromo-2-methoxybenzene (**9**) (198 mg, 0.68 mmol) in dry THF (5 mL) at −78 °C under N_2_ was slowly added *N-*BuLi (0.27 mL, 2.5 M in *N*-pentane, 0.68 mmol). After being stirred for 30 min, a solution of **13** (150 mg, 0.27 mmol) in dry THF (1.5 mL) was added slowly via a syringe. After the mixture was stirred at −78 °C for 2 h, the reaction was quenched by addition of a saturated aqueous solution of NH_4_Cl (10 mL) and extracted with EtOAc (10 mL × 3). The combined organic layers were dried over anhydrous sodium sulfate, filtered and concentrated in vacuum. The residue was purified quickly by flash column chromatography (20% EtOAc/petroleum ether, *R*_f_ = 0.2) to give unstable **14** (177 mg, 85%) as a light red solid and **14A** (10 mg, 5%) as a light yellow solid. mp for **14**, 76.5–77.4 °C; ^1^H NMR (600 MHz, CDCl_3_) δ 2.39 (s, 3H), 3.38 (br s, 3H), 3.45 (s, 3H), 4.90 (d, *J* = 11.4 Hz, 1H), 4.94 (d, *J* = 11.4 Hz, 1H), 5.01 (br s, 2H), 5.78 (br s, 1H), 6.15 (d, *J* = 8.4 Hz, 1H), 6.18 (s, 1H), 6.30 (s, 1H), 6.35–6.46 (m, 2H), 6.52 (s, 1H), 7.04 (s, 1H), 7.18 (br s, 1H), 7.28–7.42 (m, 16H), 7.87 (d, *J* = 7.8 Hz, 2H) ppm; ^13^C NMR (CDCl_3_, 150 MHz) δ 21.59, 54.85, 55.56, 61.78, 69.69, 70.00, 98.22, 99.63, 100.55, 104.06, 104.23, 108.66, 111.62, 120.16, 122.53, 123.22, 126.26, 127.26, 127.26, 127.48, 127.48, 127.52, 127.52, 127.78, 127.87, 128.20, 128.41, 128.41, 128.57, 128.57, 129.75, 129.75, 130.03, 131.52, 132.41, 135.98, 136.32, 137.07, 144.86, 156.26, 158.52, 158.81, 159.07, 161.46, 171.10 ppm; HRMS (M + Na^+^) calcd. for C_45_H_40_N_2_NaO_8_S 791.2403, found 791.2410; IR (KBr) 3423, 1608, 1502, 1454, 1411, 1372, 1278, 1198, 1175, 1123, 1034, 945, 738, 697, 593 cm^−1^.

4,4,6-Tris(4-(benzyloxy)-2-methoxyphenyl)-2-tosyl-4,6-dihydro-2*H*-dipyrrolo[2,1-c:3′,4′-e][1,4]oxazepine (**14A**). mp 105.6–106.4 °C; ^1^H NMR (400 MHz, CDCl_3_) δ 2.25 (s, 3H), 3.08 (s, 3H), 3.55 (s, 3H), 3.83 (s, 3H), 4.95 (s, 2H), 5.00 (s, 4H), 5.59 (s, 1H), 6.09 (d, *J* = 2.4 Hz, 1H), 6.16–6.18 (m, 2H), 6.38–6.42 (m, 4H), 6.50–6.51 (m, 2H), 6.67 (td, *J* = 8.8, 2.4 Hz, 2H), 7.00–7.02 (m, 6H), 7.30–7.44 (m, 15H), 7.72 (dd, *J* = 8.8, 2.4 Hz, 1H) ppm; ^13^C NMR (CDCl_3_, 100 MHz) δ 21.68, 54.24, 54.69, 55.14, 67.95, 68.37, 68.62, 78.68, 98.24, 98.55, 98.63, 98.86, 99.70, 100.29, 103.14, 103.63, 103.91, 103.97, 105.72, 107.05, 109.16, 117.71, 118.71, 119.37, 120.79, 122.51, 124.31, 125.42, 125.42, 125.52, 126.38, 126.38, 126.58, 126.58, 126.84, 126.99, 127.47, 127.47, 127.47, 127.47, 127.59, 127.59, 128.40, 128.40, 128.49, 128.59, 129.78, 131.74, 135.03, 135.96, 136.14, 136.84, 142.76, 155.71, 157.34, 157.80, 157.85, 158.09, 158.73 ppm; HRMS (M + Na^+^) calcd. for C_59_H_52_N_2_NaO_9_S 987.3291, found 987.3297; IR (KBr) 3429, 2925, 1609, 1585, 1502, 1453, 1416, 1262, 1193, 1139, 1037, 964, 806, 698, 590 cm^−1^. 

(1′-Tosyl-1′*H*-1,3′-bipyrrole-2,2′-diyl)bis((4-(benzyloxy)-2-methoxyphenyl)methanone) (**15**). To a solution of **14** (127 mg, 0.17 mmol) in dry DMSO (4 mL) was stepwise added IBX (116 mg, 0.41 mmol) at room temperature. The mixture was allowed to warm up to 50 °C and stir for about 3.5 h. The mixture was quenched with water (10 mL) and extracted with EtOAc (10 mL × 3). The combined organic layers were dried over anhydrous sodium sulfate, filtered, and concentrated in vacuum. The residue was purified by flash column chromatography (20% EtOAc/petroleum ether, *R*_f_ = 0.2) to give **15** (115 mg, 90%) as a pale brown solid. mp 147.9–152.7 °C; ^1^H NMR (400 MHz, CDCl_3_) δ 2.42 (s, 3H), 3.64 (s, 3H), 3.73 (s, 3H), 4.98 (s, 2H), 5.03 (s, 2H), 5.87 (t, *J* = 3.2 Hz, 1H), 6.28–6.33 (m, 3H), 6.42–6.45 (m, 2H), 6.54 (d, *J* = 2.0 Hz, 1H), 6.72 (t, *J* = 2.0 Hz, 1H), 7.09 (d, *J* = 8.4 Hz, 1H), 7.31–7.46 (m, 15H), 7.84 (d, *J* = 8.4 Hz, 1H) ppm; ^13^C NMR (CDCl_3_, 100 MHz) δ 21.55, 40.76, 55.49, 55.56, 69.75, 69.95, 98.89, 99.58, 103.93, 104.75, 108.56, 111.99, 120.90, 122.10, 122.65, 122.94, 127.18, 127.18, 127.38, 127.38, 128.03, 128.03, 128.08, 128.08, 128.48, 128.48, 128.50, 128.57, 129.47, 129.47, 131.54, 131.78, 132.10, 132.32, 134.01, 135.79, 136.01, 136.22, 144.86, 159.22, 160.38, 161.52, 163.17, 182.48, 183.62 ppm; HRMS (M + H^+^) calcd. for C_45_H_39_N_2_O_8_S 767.2427, found 767.2414; IR (KBr) 3433, 3113, 2937, 2874, 1634, 1599, 1499, 1450, 1265, 1170, 1029, 746, 667, 585 cm^−1^.

(1′-Tosyl-1′*H*-1,3′-bipyrrole-2,2′-diyl)bis((4-hydroxy-2-methoxyphenyl)methanone) (**16**). To a solution of **15** (1.00 g, 1.30 mmol) in a 3:1 mixture of MeOH/EtOAc (10 mL) was added Pd/C (0.54 g, 0.13 mmol, purity: 5%) under 1 atm H_2_. The mixture was stirred for about 12 h at room temperature. The suspension was filtered and the filtrate was washed with acetone (100 mL). The combined organic layers were concentrated in vacuum and the residue was purified by flash column chromatography (20% acetone/petroleum ether, *R*_f_ = 0.3) to give **16** (0.65 g, 85%) as a gray solid. mp 157.3–160.3 °C; ^1^H NMR (400 MHz, CDCl_3_) δ 2.31 (s, 3H), 3.35 (s, 3H), 3.38 (s, 3H), 5.90 (s, 1H), 5.99 (d, *J* = 8.4 Hz, 1H), 6.05 (s, 1H), 6.20 (d, *J* = 8.0 Hz, 1H), 6.24 (s, 1H), 6.39 (d, *J* = 2.4 Hz, 1H), 6.42 (d, *J* = 2.8 Hz, 1H), 6.74 (s, 1H), 6.94 (d, *J* = 8.0 Hz, 1H), 7.20–7.24 (m, 3H), 7.49 (d, *J* = 3.2 Hz, 1H), 7.85 (d, *J* = 7.6 Hz, 2H), 8.29 (br s, 1H), 8.88 (br s, 1H) ppm; ^13^C NMR (acetone-*d*_6_, 100 MHz) δ 21.52, 55.70, 55.79, 99.45, 100.04, 106.99, 107.75, 109.35, 112.76, 120.50, 122.35, 122.46, 123.34, 128.81, 128.81, 130.01, 130.46, 130.46, 131.23, 132.07, 132.20, 134.03, 134.68, 137.20, 146.06, 160.09, 161.31, 162.12, 163.86, 183.31, 183.35 ppm; HRMS (M + Na^+^) calcd. for C_31_H_26_N_2_NaO_8_S 609.1308, found 609.1313; IR (KBr) 3422, 2934, 2853, 1606, 1466, 1436, 1313, 1268, 1174, 936, 671 cm^−1^.

1′*H*-1,3′-Bipyrrole-2,2′-diylbis((4-hydroxy-2-methoxyphenyl)methanone) (**17**). To a solution of **16** (100 mg, 0.17 mmol) in a 1:1 mixture of MeOH/THF (5 mL) was added KOH (39 mg, 0.69 mmol) at room temperature. After being stirred for 2 h, the mixture was adjusted to pH 7.0 with 0.5 N HCl and extracted with EtOAc (10 mL × 3). The combined organic layers were dried over anhydrous sodium sulfate, filtered and concentrated in vacuum. The residue was purified by flash column chromatography (50% EtOAc/petroleum ether, *R*_f_ = 0.3) to give **17** (66 mg, 90%) as a brown solid. mp 83.0–85.7 °C; ^1^H NMR (400 MHz, CD_3_OD) δ 3.55 (s, 3H), 3.66 (s, 3H), 6.00 (t, *J* = 2.4 Hz, 1H), 6.09 (dd, *J* = 8.4, 2.0 Hz, 1H), 6.17 (d, *J* = 2.0 Hz, 1H), 6.22 (d, *J* = 2.0 Hz, 1H), 6.33 (dd, *J* = 8.4, 2.0 Hz, 1H), 6.36 (dd, *J* = 4.0, 2.0 Hz, 1H), 6.41 (d, *J* = 2.0 Hz, 1H), 6.83 (s, 1H), 6.99 (d, *J* = 8.4 Hz, 1H), 7.02 (t, *J* = 1.6 Hz, 1H), 7.04 (d, *J* = 8.4 Hz, 1H) ppm; ^13^C NMR (CD_3_OD, 100 MHz) δ 55.69, 55.85, 99.75, 100.10, 106.98, 107.78, 109.96, 110.64, 120.78, 121.46, 124.03, 124.43, 127.17, 132.42, 132.57, 133.67, 133.84, 133.96, 160.55, 161.11, 162.41, 162.56, 185.53, 185.61 ppm; HRMS (M + H^+^) calcd. for C_24_H_21_N_2_O_6_ 433.1400, found 433.1379; IR (KBr) 3293, 2938, 1697, 1610, 1465, 1407, 1308, 1269, 1201, 1163, 1121, 1031, 959, 868, 748 cm^−1^.

(4,4′,5,5′-Tetrachloro-1′*H*-1,3′-bipyrrole-2,2′-diyl)bis((5-chloro-4-hydroxy-2-methoxyphenyl) methanone) (**18**). To a solution of **17** (20 mg, 0.05 mmol) in dry MeCN (2 mL) at room temperature was gradually added NCS (37 mg, 0.28 mmol). After being stirred for about 6 h at room temperature, the mixture was quenched with water (15 mL) and extracted with EtOAc (15 mL × 3). The combined organic layers were dried over anhydrous sodium sulfate, filtered, and concentrated in vacuum. The residue was purified by flash column chromatography (33% EtOAc/petroleum ether, *R*_f_ = 0.2) to give **18** (11 mg, 40%) as a yellow solid. mp 102.7–104.7 °C; ^1^H NMR (400 MHz, acetone-*d*_6_) δ 3.65 (s, 3H), 3.71 (s, 3H), 6.52 (s, 1H), 6.55 (s, 1H), 6.71 (s, 1H), 7.10 (s, 1H), 7.23 (s, 1H) ppm; ^13^C NMR (CD_3_OD + CDCl_3_, 100 MHz) δ 55.99, 56.03, 100.22, 100.86, 110.21, 111.77, 111.83, 112.37, 119.31, 120.11, 120.80, 120.86, 124.20, 124.960, 125.98, 130.88, 131.42, 132.46, 157.01, 157.51, 158.39, 159.10, 181.27, 181.86 ppm; HRMS (M + H^+^) calcd. for C_24_H_15_Cl_6_N_2_O_6_ 636.9061, found 636.9073; IR (KBr) 3441, 3230, 3130, 2936, 2855, 1723, 1628, 1602, 1403, 1298, 1271, 1215, 1037, 994, 745, 666 cm^−1^.

(4,4′,5,5′-Tetrachloro-1′*H*-1,3′-bipyrrole-2,2′-diyl)bis((5-chloro-2,4-dihydroxyphenyl)methanone) (**19**). To a solution of **18** (14 mg, 0.02 mmol) in dry CH_2_Cl_2_ (4 mL) was slowly added a solution of BBr_3_ (19 mg, 0.08 mmol) in dry CH_2_Cl_2_ (1 mL) via a syringe under N_2_ at −78 °C. After being stirred for 0.5 h, the mixture was quenched by addition of water (10 mL) and extracted with CH_2_Cl_2_ (10 mL × 3). The combined organic layers were dried over anhydrous sodium sulfate, filtered, and concentrated in vacuum. The residue was purified by flash column chromatography (25% EtOAc/petroleum ether, *R*_f_ = 0.3) to give **19** (11 mg, 85%) as a pale brown solid. mp 145.7–147.7 °C; ^1^H NMR (400 MHz, CD_3_OD) δ 6.27 (s, 1H), 6.32 (s, 1H), 7.31 (s, 1H), 7.41 (s, 1H), 7.96 (s, 1H) ppm; ^13^C NMR (CD_3_OD, 100 MHz) δ 101.49, 105.13, 105.25, 110.00, 112.90, 113.27, 114.67, 119.42, 120.24, 123.00, 123.87, 125.98, 130.91, 132.64, 132.66, 133.99, 134.07, 163.85, 164.48, 165.02, 184.46, 185.64 ppm; HRMS (M + Na^+^) calcd. for C_22_H_10_Cl_6_N_2_NaO_6_ 630.8568, found 630.8581; IR (KBr) 3425, 2961, 2924, 2854, 1654, 1622, 1414, 1384, 1358, 1258, 1024, 800 cm^−1^. HPLC purity, 95.4% (Flow rate: 1.0 mL/min; Column: Phenomenex C6-phenyl, 5 μm, 150 × 4.6 mm; Wavelength: UV 254 nm; Temperature: 25 °C; Mobile phase: MeOH:H_2_O = 80:20; *t*_R_ = 5.1 min).

(1′-Tosyl-1′*H*-1,3′-bipyrrole-2,2′-diyl)bis(((2-methoxy-4-hydroxytrifluoromethanesulfonate)phenyl) methanone) (**20**). To a solution of **16** (0.50 g, 0.85 mmol) in dry MeCN (10 mL) at −30 °C under N_2_ was slowly added DIEA (0.44 g, 3.4 mmol). After being stirred for 5 min, Tf_2_O (0.72 g, 2.60 mmol) was added slowly via a syringe. The mixture was stirred for about 3 h at room temperature and quenched with water (10 mL) and extracted with EtOAc (10 mL × 3). The combined organic layers were dried over anhydrous sodium sulfate, filtered and concentrated in vacuum. The residue was purified by flash column chromatography (15% EtOAc/petroleum ether, *R*_f_ = 0.3) to give **20** (0.62 g, 86%) as a pale brown solid. mp 116.3–120.0 °C; ^1^H NMR (400 MHz, CDCl_3_) δ 2.46 (s, 3H), 3.71 (s, 3H), 3.79 (s, 3H), 5.90 (t, *J* = 2.8 Hz, 1H), 6.31 (d, *J* = 2.8 Hz, 1H), 6.38 (d, *J* = 3.2 Hz, 1H), 6.53 (d, *J* = 1.6 Hz, 1H), 6.63 (dd, *J* = 8.4, 1.6 Hz, 1H), 6.70 (s, 1H), 6.80 (s, 1H), 6.83 (d, *J* = 8.4 Hz, 1H), 7.22 (d, *J* = 8.4 Hz, 1H), 7.39 (d, *J* = 8.4 Hz, 3H), 7.67 (d, *J* = 3.6 Hz, 1H), 7.99 (d, *J* = 8.0 Hz, 2H) ppm; ^13^C NMR (CDCl_3_, 100 MHz) δ 21.59, 56.01, 56.10, 104.81, 105.22, 109.65, 111.29, 112.18, 112.23, 117.08, 120.28, 123.86, 126.14, 127.57, 128.07, 128.46, 128.46, 128.97, 129.68, 129.68, 130.45, 131.78, 132.18, 132.70, 134.11, 135.64, 145.44, 150.99, 151.83, 158.38, 159.05, 181.57, 181.98 ppm; HRMS (M + H^+^) calcd. for C_33_H_25_F_6_N_2_O_12_S_3_ 851.0474, found 851.0480; IR (KBr) 3444, 3121, 2950, 2871, 1642, 1600, 1493, 1426, 1269, 1243, 1141, 948, 827, 581 cm^−1^.

(4,4′,5,5′-Tetrachloro-1′-tosyl-1′*H*-1,3′-bipyrrole-2,2′-diyl)bis(((2-methoxy-4-hydroxytrifluoromethanesulfonate)phenyl)methanone) (**21**). To a solution of **20** (0.50 g, 0.59 mmol) in dry DMF (10 mL) at room temperature was gradually added NCS (0.51 g, 3.8 mmol). After being stirred for about 3 h at room temperature, the mixture was quenched with water (15 mL), and extracted with EtOAc (15 mL × 3). The combined organic layers were dried over anhydrous sodium sulfate, filtered and concentrated in vacuum. The residue was purified by flash column chromatography (12% EtOAc/petroleum ether, *R*_f_ = 0.2) to give **21** (0.20 g, 35%) as a yellow solid. mp 81.7–83.3 °C; ^1^H NMR (400 MHz, CD_3_OD) δ 2.49 (s, 3H), 3.50 (s, 3H), 3.61 (s, 3H), 6.41 (s, 1H), 6.57 (d, *J* = 8.0 Hz, 1H), 6.82 (d, *J* = 2.0 Hz, 1H), 6.90 (d, *J* = 2.0 Hz, 1H), 6.95 (dd, *J* = 8.8, 2.4 Hz, 1H), 7.32 (d, *J* = 8.4 Hz, 1H), 7.51 (d, *J* = 12.0 Hz, 2H), 7.64 (d, *J* = 8.4 Hz, 1H), 7.92 (d, *J* = 8.4 Hz, 2H) ppm; ^13^C NMR (CDCl_3_, 100 MHz) δ 21.61, 55.90, 56.13, 104.61, 105.00, 112.01, 112.54, 113.03, 113.61, 116.93, 119.59, 120.12, 120.80, 125.09, 127.11, 128.01, 128.40, 128.40, 129.32, 129.94, 129.94, 131.06, 131.30, 132.63, 132.86, 133.68, 146.57, 151.94, 152.94, 158.53, 159.68, 180.21, 181.56 ppm; HRMS (M + H^+^) calcd. for C_33_H_21_Cl_4_F_6_N_2_O_12_S_3_ 986.8915, found 986.8926; IR (KBr) 3446, 2923, 2853, 1653, 1603, 1491, 1428, 1270, 1216, 1140, 996, 950, 831, 585 cm^−1^.

(4,4′,5,5′-Tetrachloro-1′-tosyl-1′*H*-1,3′-bipyrrole-2,2′-diyl)bis((2-hydroxy-4-hydroxytrifluoromethanesulfonate)phenyl)methanone) (**22**). To a solution of **21** (37 mg, 0.03 mmol) in dry CH_2_Cl_2_ (5 mL) was slowly added a solution of BBr_3_ (56 mg, 0.22 mmol) in dry CH_2_Cl_2_ (1 mL) via a syringe under N_2_ at −78 °C. After being stirred for 0.5 h, the mixture was quenched by addition of water (10 mL), and extracted with CH_2_Cl_2_ (10 mL × 3). The combined organic layers were dried over anhydrous sodium sulfate, filtered and concentrated in vacuum. The residue was purified by flash column chromatography (10% EtOAc/petroleum ether, *R*_f_ = 0.3) to give **22** (28 mg, 78%) as a yellow solid. mp 71.3–73.0 °C; ^1^H NMR (400 MHz, CD_3_OD) δ 2.48 (s, 3H), 6.41 (s, 1H), 6.72–6.95 (m, 4H), 7.35 (d, *J* = 8.0 Hz, 2H), 7.50 (d, *J* = 8.8 Hz, 2H), 7.79 (d, *J* = 8.0 Hz, 2H), 11.28 (br s, 1H) ppm; ^13^C NMR (CDCl_3_, 100 MHz) δ 21.76, 110.86, 111.12, 111.12, 111.12, 112.21, 112.40, 112.87, 113.93, 116.93, 116.94, 118.33, 118.35, 120.15, 122.32, 125.44, 128.30, 128.30, 130.18, 130.18, 132.90, 134.18, 135.40, 135.42, 135.43, 147.32, 154.40, 163.51, 163.78, 186.18, 188.56 ppm; HRMS (M + H^+^) calcd. for C_31_H_17_Cl_4_F_6_N_2_O_12_S_3_ 958.8602, found 958.8610; IR (KBr) 3445, 3134, 2920, 2851, 1742, 1631, 1598, 1430, 1385, 1430, 1216, 1140, 970, 842, 583 cm^−1^.

(4,4′,5,5′-Tetrachloro-1′*H*-1,3′-bipyrrole-2,2′-diyl)bis(((2-hydroxy-4-hydroxytrifluoromethanesulfonate)phenyl)methanone) (**23**). To a solution of **22** (165 mg, 0.17 mmol) in a 1:1 mixture of MeOH/THF (3 mL) was added KOH (39 mg, 0.69 mmol) at room temperature. After being stirred for 15 min, the mixture was adjusted to pH 7.0 with 0.5 N HCl and extracted with EtOAc (10 mL × 3). The combined organic layers were dried over anhydrous sodium sulfate, filtered, and concentrated in vacuum. The residue was purified by flash column chromatography (33% EtOAc/petroleum ether, *R*_f_ = 0.3) to give **23** (125 mg, 90.6%) as a light yellow solid. mp 65.7–67.7 °C; ^1^H NMR (400 MHz, CDCl_3_) δ 6.17 (s, 1H), 6.53 (dd, *J* = 7.6, 2.0 Hz, 1H), 6.81 (dd, *J* = 8.8, 2.0 Hz, 1H), 6.87 (d, *J* = 2.4 Hz, 1H), 6.93 (d, *J* = 2.4 Hz, 1H), 7.40 (br s, 1H), 7.57 (d, *J* = 8.8 Hz, 1H), 9.61 (br s, 1H), 10.67 (s, 1H), 11.49 (s, 1H) ppm; ^13^C NMR (CDCl_3_, 100 MHz) δ 109.34, 111.21, 111.33, 111.61, 112.44, 115.90, 117.01, 118.50, 118.60, 118.89, 122.60, 123.61, 124.41, 124.92, 132.11, 135.49, 151.32, 153.72, 154.72, 161.95, 162.77, 164.22, 184.70, 186.90 ppm; HRMS (M + H^+^) calcd. for C_24_H_11_Cl_4_F_6_N_2_O_10_S_2_ 804.8513, found 804.8529; IR (KBr) 3380, 3264, 1627, 1597, 1497, 1429, 1217, 1139, 1107, 970, 942, 605 cm^−1^. HPLC purity, 96.0% (Flow rate: 1.0 mL/min; Column: Phenomenex C6-phenyl, 5 μm, 150 × 4.6 mm; Wavelength: UV 254 nm; Temperature: 25 °C; Mobile phase: MeOH:H_2_O = 75:25; *t*_R_ = 17.9 min).

(4,4′,5,5′-Tetrachloro-1′*H*-1,3′-bipyrrole-2,2′-diyl)bis((2,4-dihydroxyphenyl)methanone) (**24**). To a solution of **23** (5.0 mg, 0.006 mmol) in DMSO (1 mL) was added a solution of KF (1.1 mg, 0.018 mmol) in water (0.1 mL) at room temperature. After being stirred for about 3 h, the mixture was added with water (5 mL) and extracted with EtOAc (5 mL × 3). The combined organic layers were dried over anhydrous sodium sulfate, filtered, and concentrated in vacuum. The residue was purified by flash column chromatography (40% EtOAc/petroleum ether, *R*_f_ = 0.3) to give **24** (2.6 mg, 75%) as a pale yellow solid. mp 103.3–105.3 °C; ^1^H NMR (400 MHz, acetone-*d*_6_) δ 6.10 (s, 1H), 6.19 (dd, *J* = 8.8, 2.4 Hz, 1H), 6.22–6.23 (m, 2H), 6.28 (d, *J* = 8.0 Hz, 1H), 7.62 (d, *J* = 8.8 Hz, 1H), 8.09 (br s, 1H), 12.03 (br s, 1H) ppm; ^13^C NMR (CDCl_3_, 100 MHz) δ 103.19, 103.49, 107.73, 108.14, 108.30, 108.35, 109.60, 111.44, 113.20, 113.77, 114.26, 115.07, 120.82, 123.89, 126.11, 134.16, 137.03, 162.89, 165.67, 166.61, 167.02, 185.94 ppm; HRMS (M + H^+^) calcd. for C_22_H_13_Cl_4_N_2_O_6_ 540.9528, found 540.9536; IR (KBr) 3400, 3282, 2958, 2922, 2851, 1626, 1596, 1447, 1333, 1266, 1177, 978, 796 cm^−1^. HPLC purity, 98.2% (Flow rate: 1.0 mL/min; Column: Waters C8, 5 μm, 150 × 4.6 mm; Wavelength: UV 254 nm; Temperature: 25 °C; Mobile phase: MeOH:H_2_O = 65:35; *t*_R_ = 6.2 min).

(2-((*tert*-Butyldimethylsilyloxy)methyl)-1′-tosyl-1′*H*-1,3′-bipyrrol-2′-yl)(2-methoxy-4-(trifluoromethyl)phenyl)methanol (**25**). To a solution of 1-bromo-2-methoxy-4-(trifluoromethyl)benzene (69.0 mg, 0.27 mmol) in dry THF (4 mL) at −78 °C under N_2_ was slowly added *N-*BuLi (0.11 mL, 2.5 M in *N*-pentane, 0.27 mmol). After being stirred for 30 min, a solution of **8** (50 mg, 0.11 mmol) in dry THF (1 mL) was added slowly via a syringe. The mixture was stirred for about 2 h and quenched by addition of a saturated aqueous NH_4_Cl (15 mL) solution, and extracted with EtOAc (10 mL × 3). The combined organic layers were dried over anhydrous sodium sulfate, filtered and concentrated in vacuum. The residue was purified by flash column chromatography (15% EtOAc/petroleum ether, *R*_f_ = 0.2) to give **25** (56 mg, 81%) as a pale brown solid. mp 34.7–36.7 °C; ^1^H NMR (400 MHz, CDCl_3_) δ 0.001 (s, 3H), 0.07 (s, 3H), 0.83 (s, 9H), 2.41 (s, 3H), 2.94 (d, *J* = 4.0 Hz, 1H), 3.73 (s, 3H), 4.43 (d, *J* = 12.0 Hz, 1H), 4.75 (d, *J* = 12.0 Hz, 1H), 5.81–5.84 (m, 2H), 6.10 (t, *J* = 3.2 Hz, 1H), 6.34 (d, *J* = 3.6 Hz, 1H), 6.66 (dd, *J* = 3.6, 2.0 Hz, 1H), 6.99 (s, 1H), 7.18 (d, *J* = 8.0 Hz, 1H), 7.24 (d, *J* = 3.6 Hz, 1H), 7.30 (d, *J* = 8.0 Hz, 2H), 7.55 (d, *J* = 8.0 Hz, 1H), 7.85 (d, *J* = 8.4 Hz, 2H) ppm; ^13^C NMR (CDCl_3_, 100 MHz) δ −5.71, −5.67, 18.53, 21.56, 25.92, 25.92, 25.92, 53.27, 55.53, 62.79, 106.87, 106.91, 108.05, 108.43, 111.37, 117.21, 117.25, 121.56, 123.91, 126.96, 127.01, 127.35, 128.40, 129.44, 129.87, 134.75, 134.76, 135.81, 136.29, 145.17, 156.02 ppm; HRMS (M + Na^+^) calcd. for C_31_H_37_F_3_N_2_NaO_5_SSi 657.2042, found 657.2040; IR (KBr) 3383, 3146, 2956, 2929, 2857, 1734, 1594, 1465, 1415, 1377, 1329, 1241, 1175, 1123, 1032, 841, 778, 670 cm^−1^.

(2-((*tert*-Butyldimethylsilyloxy)methyl)-1′-tosyl-1′*H*-1,3′-bipyrrol-2′-yl)(2-methoxy-4-(trifluoromethyl)phenyl)methanone (**26**). To a solution of **25** (463 mg, 0.73 mmol) in dry DMSO (10 mL) was added IBX (408 mg, 1.46 mmol) at room temperature. The mixture was allowed to warm up to 50 °C and stir additionally for about 3.5 h. The mixture was quenched with water (15 mL) and extracted with EtOAc (10 mL × 3). The combined organic layers were dried over anhydrous sodium sulfate, filtered, and concentrated in vacuum. The residue was purified by flash column chromatography (15% EtOAc/petroleum ether, *R*_f_ = 0.2) to give **26** (403 mg, 85%) as a pale brown solid. mp 38.0–40.3 °C; ^1^H NMR (400 MHz, CDCl_3_) δ −0.006 (s, 6H), 0.85 (s, 9H), 2.40 (s, 3H), 3.82 (s, 3H), 4.68 (s, 3H), 6.23 (dd, *J* = 4.0, 2.4 Hz, 1H), 6.33 (d, *J* = 3.6 Hz, 1H), 6.64 (dd, *J* = 4.0, 1.6 Hz, 1H), 7.09 (t, *J* = 2.0 Hz, 1H), 7.21–7.23 (m, 2H), 7.28 (d, *J* = 8.0 Hz, 2H), 7.40 (d, *J* = 8.0 Hz, 1H), 7.85 (d, *J* = 8.4 Hz, 2H) ppm; ^13^C NMR (CDCl_3_, 100 MHz) δ −5.65, −5.65, 18.42, 21.55, 25.65, 25.86, 25.86, 53.48, 55.86, 108.06, 108.09, 109.50, 111.33, 116.83, 116.87, 121.33, 123.91, 127.00, 127.66, 129.27, 129.38, 129.77, 131.85, 132.60, 132.93, 132.99, 133.48, 136.64, 144.82, 157.04, 182.61 ppm; HRMS (M + Na^+^) calcd. for C_31_H_35_F_3_N_2_NaO_5_SSi 655.1886, found 655.1893; IR (KBr) 3145, 2955, 2929, 2856, 1737, 1647, 1598, 1499, 1463, 1411, 1376, 1328, 1245, 1176, 1130, 1077, 894, 838, 670 cm^−1^.

(2-(Hydroxymethyl)-1′-tosyl-1′*H*-1,3′-bipyrrol-2′-yl)(2-methoxy-4-(trifluoromethyl)phenyl)methanone (**27**). To a solution of **26** (400 mg, 0.63 mmol) in dry THF (10 mL) was added TBAF (495 mg, 1.90 mmol) at room temperature. After being stirred for about 5 h at room temperature, the mixture was quenched with water (10 mL) and extracted with EtOAc (10 mL × 3). The combined organic layers were dried over anhydrous sodium sulfate, filtered, and concentrated in vacuum. The residue was purified by flash column chromatography (20% EtOAc/petroleum ether, *R*_f_ = 0.3) to give **27** (290 mg, 87%) as a light yellow solid. mp 53.3–56.0 °C; ^1^H NMR (400 MHz, CDCl_3_) δ 2.35 (s, 3H), 2.98 (t, *J* = 6.4 Hz, 1H), 3.76 (s, 3H), 4.54 (d, *J* = 6.8 Hz, 2H), 6.24 (t, *J* = 3.6 Hz, 1H), 6.34 (d, *J* = 6.8 Hz, 1H), 6.61 (dd, *J* = 3.6, 1.2 Hz, 1H), 7.04 (s, 1H), 7.14 (s, 1H), 7.22 (d, *J* = 3.6 Hz, 1H), 7.26–7.30 (m, 3H), 7.38 (d, *J* = 8.0 Hz, 1H), 7.84 (d, *J* = 8.4 Hz, 2H) ppm; ^13^C NMR (CDCl_3_, 100 MHz) δ 21.56, 53.09, 55.92, 108.14, 108.17, 110.27, 110.92, 116.94, 116.97, 122.31, 124.59, 127.16, 128.46, 128.55, 129.15, 130.11, 132.03, 132.68, 132.89, 133.00, 133.74, 135.81, 145.48, 157.03, 183.56 ppm; HRMS (M + Na^+^) calcd. for C_25_H_21_F_3_N_2_NaO_5_S 541.1021, found 541.1027; IR (KBr) 3425, 3119, 2956, 2925, 1642, 1596, 1500, 1460, 1411, 1328, 1175, 1133, 1078, 893, 673, 602 cm^−1^.

2′-(2-Methoxy-4-(trifluoromethyl)benzoyl)-1′-tosyl-1′*H*-1,3′-bipyrrole-2-carbaldehyde (**28**). To a solution of **27** (286 mg, 0.55 mmol) in dry DMSO (10 mL) was added IBX (309 mg, 1.10 mmol) at room temperature. After being stirred for about 3.5 h, the mixture was quenched with water (15 mL) and extracted with EtOAc (10 mL × 3). The combined organic layers were dried over anhydrous sodium sulfate, filtered, and concentrated in vacuum. The residue was purified by flash column chromatography (20% EtOAc/petroleum ether, *R*_f_ = 0.3) to give **28** (242 mg, 85%) as a light brown solid. mp 114.3–117.0 °C; ^1^H NMR (400 MHz, CDCl_3_) δ 2.43 (s, 3H), 3.80 (s, 3H), 6.29 (t, *J* = 3.2 Hz, 1H), 6.48 (d, *J* = 3.6 Hz, 1H), 6.63 (d, *J* = 2.4 Hz, 1H), 7.03 (s, 1H), 7.13 (s, 1H), 7.24 (d, *J* = 7.6 Hz, 1H), 7.35 (d, *J* = 8.0 Hz, 2H), 7.46 (d, *J* = 7.6 Hz, 1H), 7.69 (d, *J* = 3.2 Hz, 1H), 7.89 (d, *J* = 8.4 Hz, 2H), 9.70 (s, 1H) ppm; ^13^C NMR (CDCl_3_, 100 MHz) δ 21.63, 55.89, 108.12, 108.15, 110.55, 111.54, 116.88, 116.92, 123.99, 125.49, 127.53, 127.90, 127.90, 129.62, 130.08, 130.08, 132.34, 132.63, 132.81, 133.15, 134.75, 138.21, 146.08, 177.23, 182.75 ppm; HRMS (M + Na^+^) calcd. for C_25_H_19_F_3_N_2_NaO_5_S 539.0864, found 539.0856; IR (KBr) 3433, 3141, 3089, 2927, 2855, 1679, 1641, 1562, 1408, 1327, 1173, 1128, 1023, 901, 812, 670 cm^−1^.

(2-(Hydroxy(2-methoxy-4-(trifluoromethyl)phenyl)methyl)-1′-tosyl-1′*H*-1,3′-bipyrrol-2′-yl)(2-methoxy-4-(trifluoromethyl)phenyl)methanone (**29**). To a solution of 1-bromo-2-methoxy-4-(trifluoromethyl)benzene (200 mg, 0.78 mmol) in dry THF (8 mL) at −78 °C under N_2_ was slowly added *N-*BuLi (0.31 mL, 2.5 M in *N*-pentane, 0.78 mmol). After being stirred for 30 min, a solution of **28** (200 mg, 0.39 mmol) in dry THF (2 mL) was added slowly via a syringe. After the mixture was stirred at −78 °C for 2 h, the reaction was quenched by addition of a saturated aqueous solution of NH_4_Cl (15 mL) and extracted with EtOAc (10 mL × 3). The combined organic layers were dried over anhydrous sodium sulfate, filtered and concentrated in vacuum. The residue was purified by flash column chromatography (15% EtOAc/petroleum ether, *R*_f_ = 0.2) to give **29** (150 mg, 56%) as a light yellow solid, **29A** (42 mg, 15%) as a light yellow solid and recovered **28** (48 mg, 24%). mp for **29**, 85.1–86.3 °C; ^1^H NMR (400 MHz, CDCl_3_) δ 2.44 (s, 3H), 3.59 (br s, 3H), 3.84 (s, 3H), 4.12 (br s, 1H), 5.77 (br s, 1H), 6.17–6.32 (m, 3H), 6.49 (br s, 1H), 6.70 (br s, 1H), 6.83 (d, *J* = 7.6 Hz, 1H), 7.13 (br s, 1H), 7.21 (br s, 1H), 7.35–7.37 (m, 4H), 7.42 (d, *J* = 2.8 Hz, 1H), 7.92 (d, *J* = 8.0 Hz, 2H) ppm; ^13^C NMR (CDCl_3_, 100 MHz) δ 21.67, 55.75, 55.88, 67.96, 105.60, 105.64, 108.13, 108.03, 111.28, 116.78, 116.78, 116.78, 120.85, 120.85, 124.97, 126.20, 127.10, 127.30, 127.63, 127.63, 129.14, 129.14, 129.98, 129.98, 132.50, 132.56, 133.21, 133.45, 135.92, 145.38, 155.48, 157.01, 180.50 ppm; HRMS (M + Na^+^) calcd. for C_33_H_26_F_6_N_2_NaO_6_S 715.1313, found 715.1287; IR (KBr) 3444, 3137, 2923, 1668, 1562, 1447, 1361, 1180, 1014, 752, 668 cm^−1^.

(2-(Hydroxy(2-methoxy-4-(trifluoromethyl)phenyl)methyl)-1′-tosyl-1′*H*-1,3′-bipyrrol-2′-yl)bis(2-methoxy-4-(trifluoromethyl)phenyl)methanol (**29A**). mp 103.7–104.5 °C; ^1^H NMR (600 MHz, CDCl_3_) δ 2.46 (s, 3H), 3.30 (s, 3H), 3.64 (s, 3H), 3.74 (br s, 3H), 5.30–5.42 (m, 5H), 5.78 (d, *J* = 8.4 Hz, 1H), 6.47 (d, *J* = 8.4 Hz, 2H), 6.75 (s, 1H), 6.81 (d, *J* = 7.2 Hz, 1H), 7.03–7.15 (m, 8H), 7.29 (d, *J* = 7.2 Hz, 1H), 7.34 (d, *J* = 7.2 Hz, 2H), 7.87 (d, *J* = 7.2 Hz, 2H) ppm; ^13^C NMR (CDCl_3_, 150 MHz) δ 21.66, 55.08, 55.37, 56.00, 61.74, 105.41, 106.67, 108.30, 109.09, 111.52, 112.15, 116.80, 116.89, 117.42, 119.68, 122.76, 122.90, 123.30, 124.57, 124.71, 125.11, 125.28, 125.78, 127.82, 128.68, 129.17, 129.38, 129.68, 129.95, 131.08, 131.36, 131.56, 132.38, 134.02, 136.38, 136.67, 144.93, 155.51, 155.51, 156.10, 157.72 ppm; HRMS (M + Na^+^) calcd. for C_41_H_33_F_9_N_2_NaO_7_S 891.1762, found 891.1716; IR (KBr) 3408, 1587, 1503, 1461, 1415, 1378, 1331, 1239, 1175, 1123, 1082, 1027, 922, 894, 860, 718, 672, 595 cm^−1^.

(1′-Tosyl-1′*H*-1,3′-bipyrrole-2,2′-diyl)bis((2-methoxy-4-(trifluoromethyl)phenyl)methanone) (**30**). To a solution of **29** (800 mg, 1.16 mmol) in dry DMSO (20 mL) was gradually added IBX (810 mg, 2.89 mmol) at room temperature. After being stirred for about 1 h, the mixture was quenched with water (30 mL) and extracted with EtOAc (15 mL × 3). The combined organic layers were dried over anhydrous sodium sulfate, filtered, and concentrated in vacuum. The residue was purified by flash column chromatography (20% EtOAc/petroleum ether, *R*_f_ = 0.2) to give **30** (670 mg, 84%) as a light yellow solid. mp 81.3–83.3 °C; ^1^H NMR (400 MHz, CDCl_3_) δ 2.45 (s, 3H), 3.78 (s, 3H), 3.80 (s, 3H), 5.89 (t, *J* = 2.8 Hz, 1H), 6.26 (d, *J* = 2.8 Hz, 1H), 6.41 (d, *J* = 3.6 Hz, 1H), 6.74 (s, 1H), 6.85 (s, 1H), 6.98 (d, *J* = 8.0 Hz, 1H), 7.12–7.19 (m, 3H), 7.37 (d, *J* = 7.6 Hz, 3H), 7.65 (d, *J* = 3.2 Hz, 1H), 7.96 (d, *J* = 8.0 Hz, 2H) ppm; ^13^C NMR (CDCl_3_, 100 MHz) δ 21.68, 55.81, 55.85, 107.74, 108.17, 109.43, 111.60, 116.43, 116.63, 123.90, 125.74, 128.13, 128.36, 128.36, 129.29, 129.66, 129.66, 130.63, 131.01, 131.61, 132.13, 132.77, 133.06, 133.83, 134.05, 134.15, 135.56, 145.37, 145.37, 157.06, 157.69, 182.04, 183.06 ppm; HRMS (M + H^+^) calcd. for C_33_H_25_F_6_N_2_O_6_S 691.1338, found 691.1336; IR (KBr) 3633, 3433, 3148, 2940, 1650, 1586, 1461, 1413, 1330, 1175, 1129, 1028, 671 cm^−1^.

1′*H*-1,3′-Bipyrrole-2,2′-diylbis((2-methoxy-4-(trifluoromethyl)phenyl)methanone) (**31**). To a solution of **30** (670 mg, 0.97 mmol) in a 1:1 mixture of MeOH/THF (10 mL) was added KOH (218 mg, 3.88 mmol) at room temperature. After being stirred for 15 min, the mixture was adjusted to pH 7.0 with 0.5 N HCl and extracted with EtOAc (10 mL × 3). The combined organic layers were dried over anhydrous sodium sulfate, filtered, and concentrated in vacuum. The residue was purified by flash column chromatography (33% EtOAc/petroleum ether, *R*_f_ = 0.3) to give **31** (494 mg, 95%) as a light yellow solid. mp 75.0–77.0 °C; ^1^H NMR (400 MHz, CDCl_3_) δ 3.76 (s, 3H), 3.84 (s, 3H), 5.84 (t, *J* = 4.0 Hz, 1H), 6.29–6.32 (m, 2H), 6.65 (s, 1H), 6.87 (s, 1H), 6.95 (d, *J* = 7.6 Hz, 1H), 7.11 (t, *J* = 3.2 Hz, 1H), 7.14 (s, 1H), 7.19–7.21 (m, 2H), 7.25–7.26 (m, 1H), 9.51 (br s, 1H) ppm; ^13^C NMR (CDCl_3_, 100 MHz) δ 55.61, 55.85, 107.31, 108.26, 109.08, 110.68, 116.64, 116.87, 116.91, 123.92, 124.21, 125.88, 128.88, 129.38, 131.32, 131.63, 131.75, 132.26, 132.50, 132.58, 132.65, 132.92, 156.39, 157.11, 182.18, 182.49 ppm; HRMS (M + H^+^) calcd. for C_26_H_19_F_6_N_2_O_4_ 537.1249, found 537.1238; IR (KBr) 3295, 2943, 1636, 1462, 1413, 1331, 1126, 1076, 928, 829, 742 cm^−1^.

1′*H*-1,3′-Bipyrrole-2,2′-diylbis((2-hydroxy-4-(trifluoromethyl)phenyl)methanone) (**32**). To a solution of **31** (100 mg, 0.19 mmol) in dry CH_2_Cl_2_ (5 mL) was slowly added a solution of BBr_3_ (233 mg, 0.93 mmol) in dry CH_2_Cl_2_ (1 mL) via a syringe under N_2_ at −78 °C. After being stirred for 0.5 h, the mixture was quenched by addition of water (10 mL) and extracted with CH_2_Cl_2_ (10 mL × 3). The combined organic layers were dried over anhydrous sodium sulfate, filtered, and concentrated in vacuum. The residue was purified by flash column chromatography (30% EtOAc/petroleum ether, *R*_f_ = 0.3) to give **32** (78 mg, 82%) as a yellow solid. mp 148.0–149.0 °C; ^1^H NMR (400 MHz, CDCl_3_) δ 6.26 (dd, *J* = 4.0, 2.8 Hz, 1H), 6.39 (s, 1H), 6.69 (d, *J* = 8.0 Hz, 1H), 6.75 (dd, *J* = 4.0, 1.2 Hz, 1H), 6.98 (t, *J* = 2.0 Hz, 1H), 7.01 (d, *J* = 8.4 Hz, 1H), 7.03 (s, 1H), 7.21 (d, *J* = 5.6 Hz, 2H), 7.37 (d, *J* = 8.0 Hz, 1H), 8.40 (d, *J* = 8.0 Hz, 1H), 9.46 (br s, 1H), 10.85 (br s, 1H), 11.42 (br s, 1H) ppm; ^13^C NMR (CDCl_3_, 100 MHz) δ 109.89, 111.00, 114.80, 114.83, 114.98, 115.02, 115.42, 115.45, 121.36, 121.80, 123.28, 124.15, 124.47, 129.99, 130.53, 130.92, 132.19, 132.41, 136.17, 136.59, 161.01, 162.11, 186.42, 187.09 ppm; HRMS (M + H^+^) calcd. for C_24_H_15_F_6_N_2_O_4_ 509.0936, found 509.0934; IR (KBr) 3334, 3148, 3080, 1636, 1591, 1562, 1412, 1337, 1231, 1130, 1068, 944, 875, 786, 748, 605 cm^−1^.

(4,4′,5,5′-Tetrachloro-1′*H*-1,3′-bipyrrole-2,2′-diyl)bis((2-hydroxy-4-(trifluoromethyl)phenyl)methanone) (**33**). To a solution of **32** (50 mg, 0.10 mmol) in dry MeCN (10 mL) at room temperature was stepwise added NCS (72 mg, 0.54 mmol). After being stirred for about 3.5 h at room temperature, the mixture was quenched with water (10 mL) and extracted with EtOAc (10 mL × 3). The combined organic layers were dried over anhydrous sodium sulfate, filtered, and concentrated in vacuum. The residue was purified by flash column chromatography (12% EtOAc/petroleum ether, *R*_f_ = 0.2) to give **33** (12 mg, 20%) as a yellow solid. mp 70.7–72.3 °C; ^1^H NMR (400 MHz, CDCl_3_) δ 6.74 (d, *J* = 8.3 Hz, 1H), 6.80 (s, 1H), 7.17 (d, *J* = 8.3 Hz, 1H), 7.20 (s, 1H), 7.31 (s, 1H), 7.58 (d, *J* = 8.2 Hz, 1H), 7.77 (d, *J* = 8.3 Hz, 1H), 10.06 (s, 1H), 10.22 (s, 1H), 11.17 (s, 1H) ppm; ^13^C NMR (CDCl_3_, 100 MHz) δ ppm 110.94, 113.57, 114.93, 115.14, 115.54, 115.94, 120.82, 121.07, 121.21, 121.49, 123.41, 123.45, 125.00, 128.25, 130.59, 132.36, 136.70, 137.03, 137.20, 137.53, 160.53, 162.37, 184.81, 186.10; HRMS (M + H^+^) calcd. for C_24_H_11_Cl_4_F_6_N_2_O_4_ 644.9372, found 644.9372; IR (KBr) 3789, 3661, 3577, 2917, 2847, 1726, 1636, 1601, 1489, 1407, 1332, 1218, 1133, 946, 832 cm^−1^. HPLC purity, 97.2% (Flow rate: 1.0 mL/min; Column: Waters C8, 5 μm, 150 × 4.6 mm; Wavelength: UV 254 nm; Temperature: 25 °C; Mobile phase: MeOH:H_2_O = 68:32; *t*_R_ = 9.2 min).

### 3.2. *In Vitro* Antibacterial Assays

A panel of multiple resistance Gram-positive and Gram-negative pathogens listed in Figure 2 and Figure 3 was used to evaluate the antibacterial activity of marinopyrrole derivatives with vancomycin as a positive control. Except for those marked with WHO, all pathogens were isolated between 2008 and 2010 in hospitals located in Beijing, Guangzhou, Sichuan, Shandong, and Jiangsu Province. The minimum inhibitory concentration (MIC) was determined as the lowest concentration of antibiotic that inhibited visible bacterial growth by the guidelines of the Clinical and Laboratory Standards Institute [25]. The concentrations of a marinopyrrole derivative are 128, 64, 32, 16, 8, 4, 2, 1, 0.5, 0.25, 0.125, 0.06, 0.03, 0.015, and 0.008 μg/mL. 

## 4. Conclusions

This article describes optimization of general synthetic routes to access novel symmetrical marinopyrrole derivatives and evaluation of their *in vitro* antibacterial activity against a panel of Gram-positive pathogens including MRSA. The efforts were focused on improving antibacterial potency with chemistry focused on synthetic strategy and route optimization. The optimized methods paved the way towards diverse sets of both symmetrical and potentially non-symmetrical marinopyrroles. The new route circumvented the low yields due to the formation of byproduct oxazepine **5** encountered from our first total synthesis [12]. The parent compound, (±)-marinopyrrole A, not only showed potent activity comparable to that of vancomycin against MRSA and MRSE, but also exhibited higher potency (64–128 fold) than vancomycin against moderately resistant VRE (Figure 2). SAR studies of derivatives have clearly demonstrated that the tetrachloro substituents on the pyrrole rings, hydroxyl groups in the *ortho* position and an electron-withdrawing group in the *meta* or *para* position on the phenyl rings are important for achieving potent antibacterial activity against the Gram-positive pathogens tested. Of particular interest, the best compound **33** showed 63–125 fold, eight-fold and four-fold more potent than vancomycin, in addition to 31 fold, four-fold and four-fold more potent than the parent marinopyrrole A (**1**), against MRSE, MSSA and MRSA, respectively (Figure 3). These results provide useful information for further optimization in the search for new-generation antibiotics against MRSA, MRSE and other pathogens. In summary, we have designed and optimized synthetic routes to access novel marinopyrrole derivatives. The SAR studies led to **33**—a compound with superior antibiotic activity to that of vancomycin against a broad spectrum of Gram-positive pathogens. Design and synthesis of novel non-symmetrical and symmetrical marinopyrroles are actively ongoing with four patent applications being filed. Their activity, selectivity, and ADME/tox data will be published in due course.

## Abbreviations

ADME: absorption, distribution, metabolism and excretion
DCM: dichloromethane
DIEA: diisopropylethylamine
DMF: dimethylformamide
DMSO: dimethyl sulfoxide
EtOAc: ethyl acetate
ESI: electrospray ionization
IBX: 2-iodoxybenzoic acid
KBr: potassium bromide
KF: potassium fluoride
MeCN: acetonitrile
MeOH: methyl alcohol
NCS: *N*-chlorosuccinimide
TBAF: tetrabutylammonium fluoride
TBDMS: *t*-butyldimethylsilyl
TBDMSCl: *t*-butyldimethylsilyl chloride
Tf: trifluoromethanesulfonyl
THF: tetrahydrofuran
WHO: World Health Organization

## Supplementary Files

Supplementary File 1 Supplementary Information (PDF, 7254 KB)
