# Supplementary material for: Marinopyrrole Derivatives as Potential Antibiotic Agents against Methicillin-Resistant Staphylococcus aureus (II)
Source: Mar Drugs. 2013 Aug 15;11(8):2927–48. doi: 10.3390/md11082927 (PMC3766874; doi:10.3390/md11082927)
Supplement: Supplementary File 1 — Supplementary Information (PDF, 7254 KB) [file marinedrugs-11-02927-s001.pdf]

Supplementary Information

Figure S1. ^{1}H NMR spectrum of **7**.

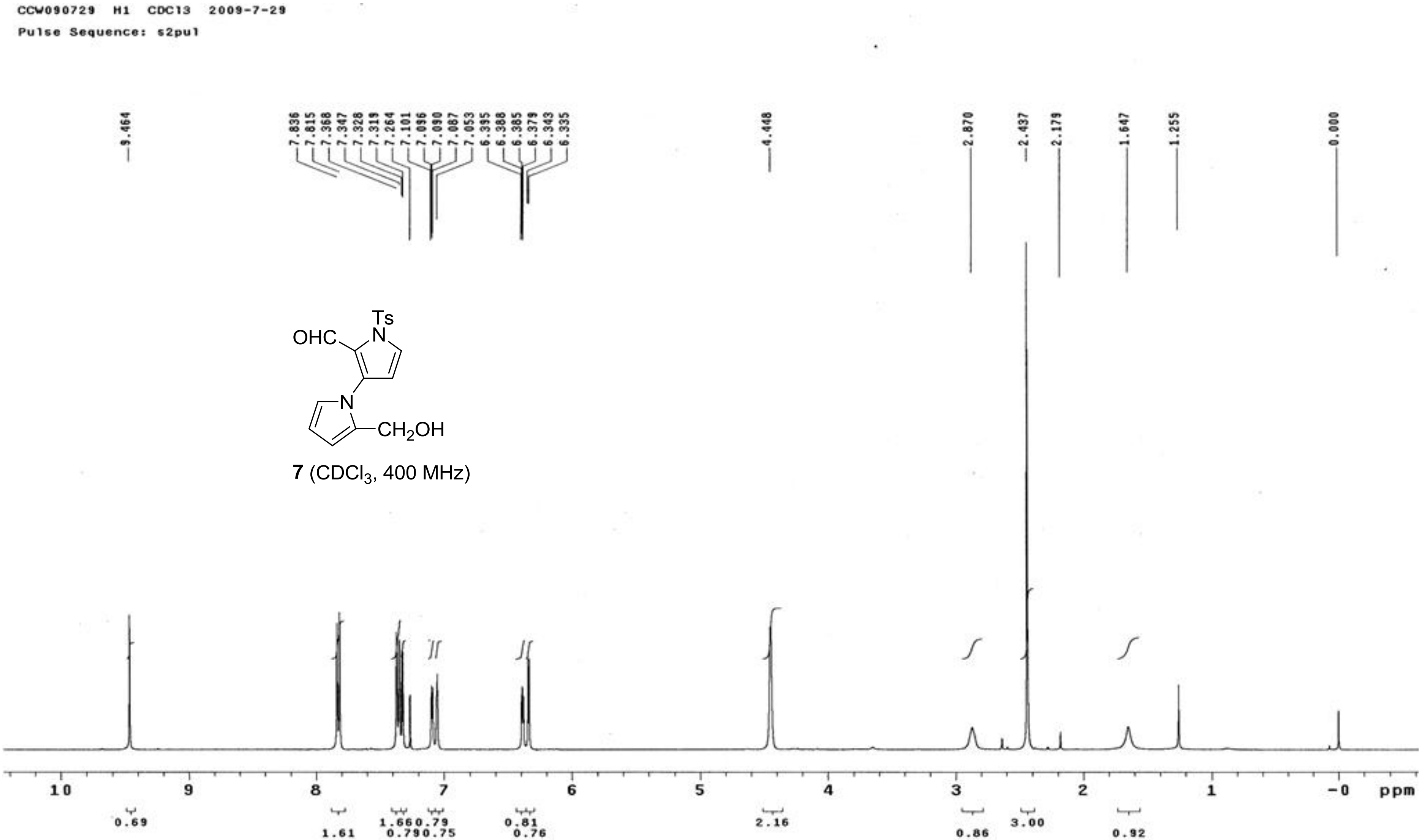

Figure S2. ^{13}C NMR spectrum of **7**.

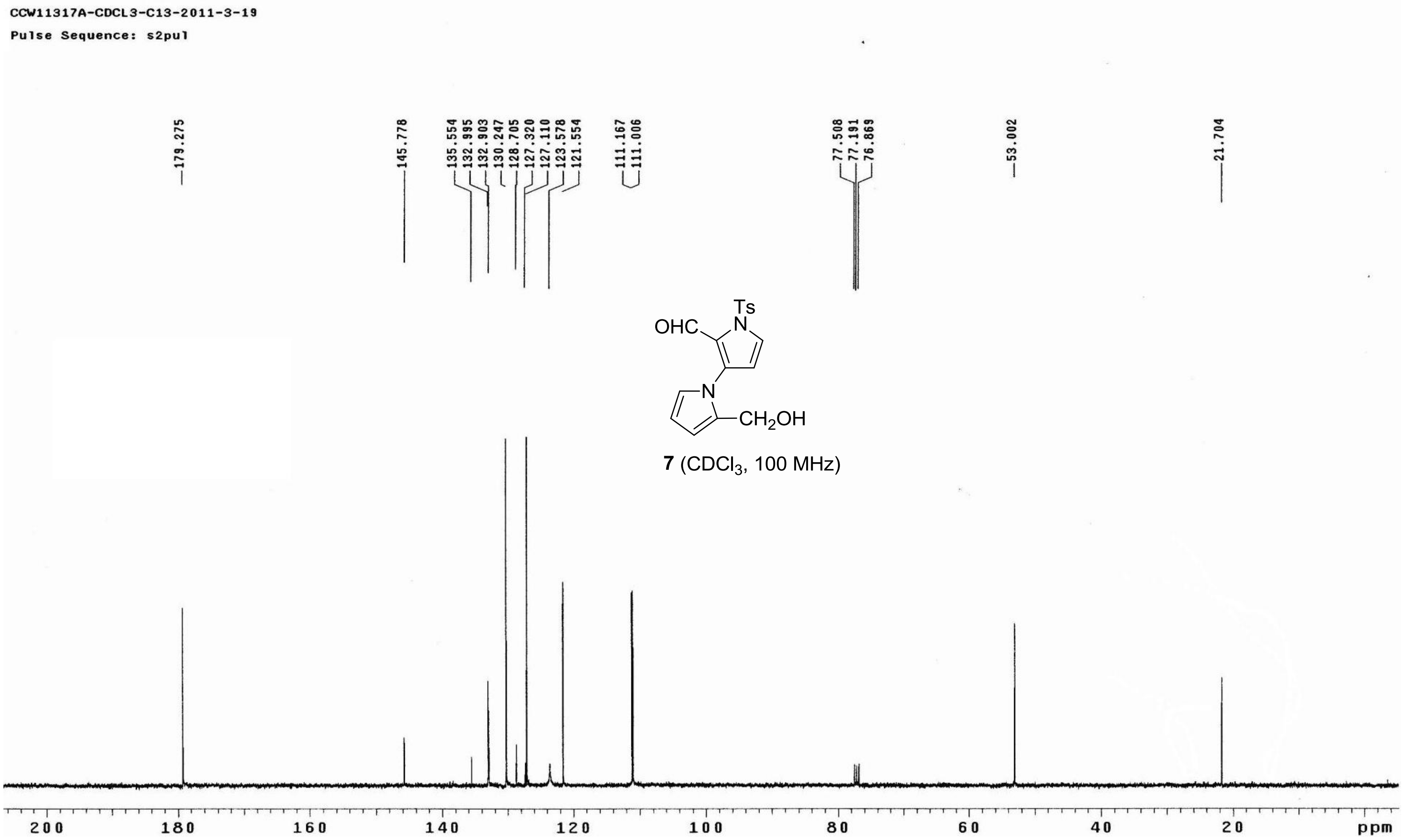

Figure S3. ^{1}H NMR spectrum of **8**.

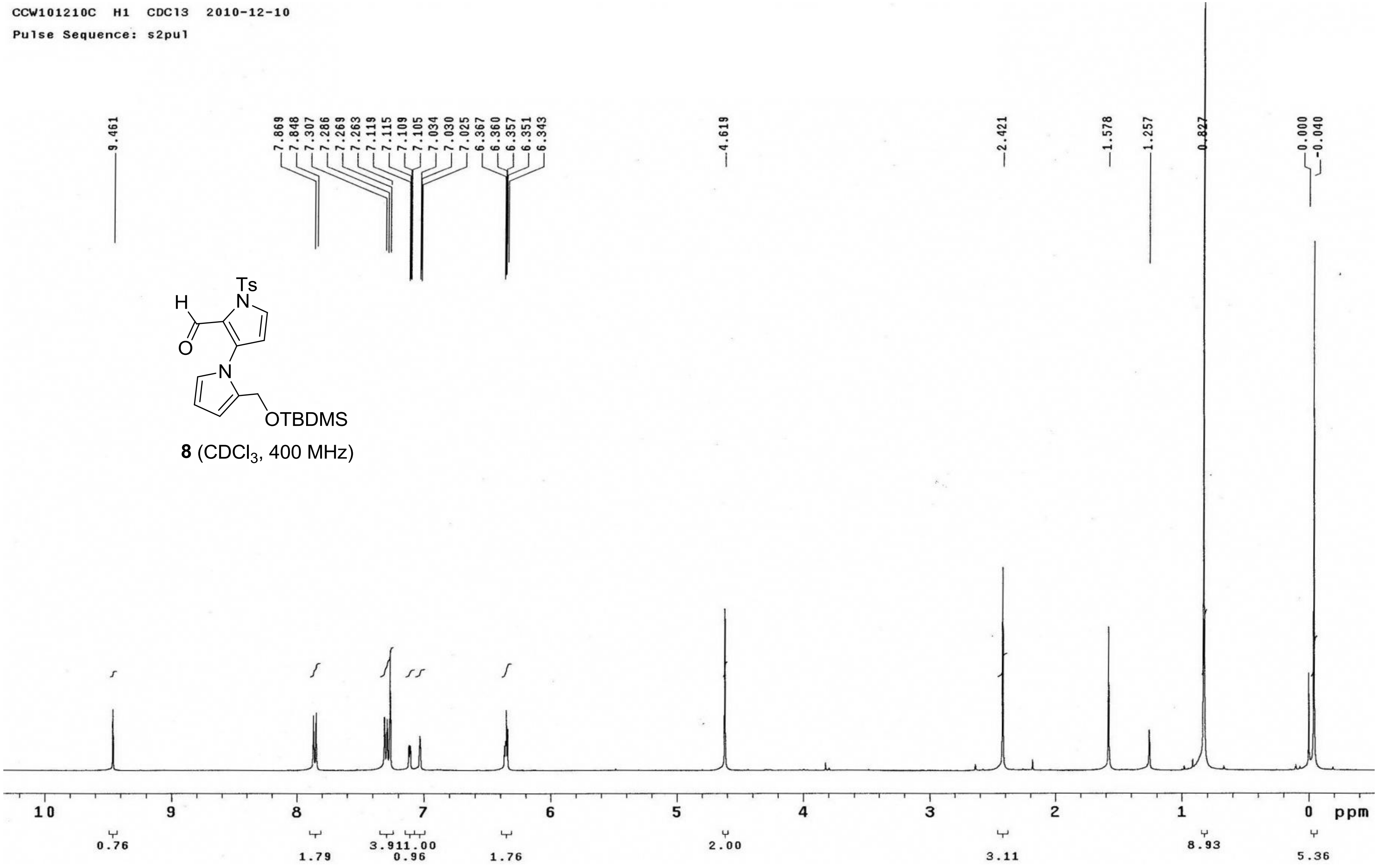

Figure S4. ^{13}C NMR spectrum of **8**.

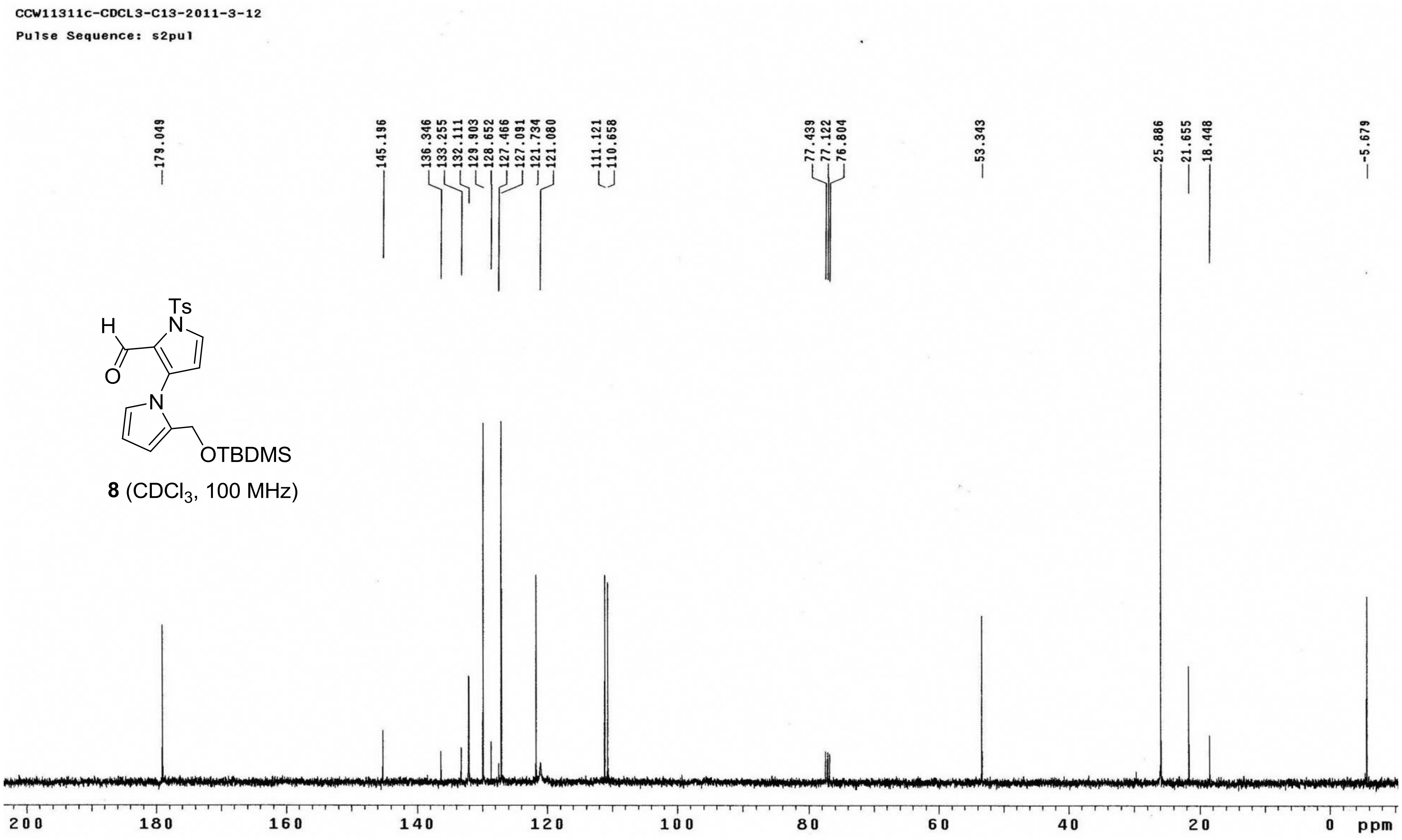

Figure S5. ^{1}H NMR spectrum of **10**.

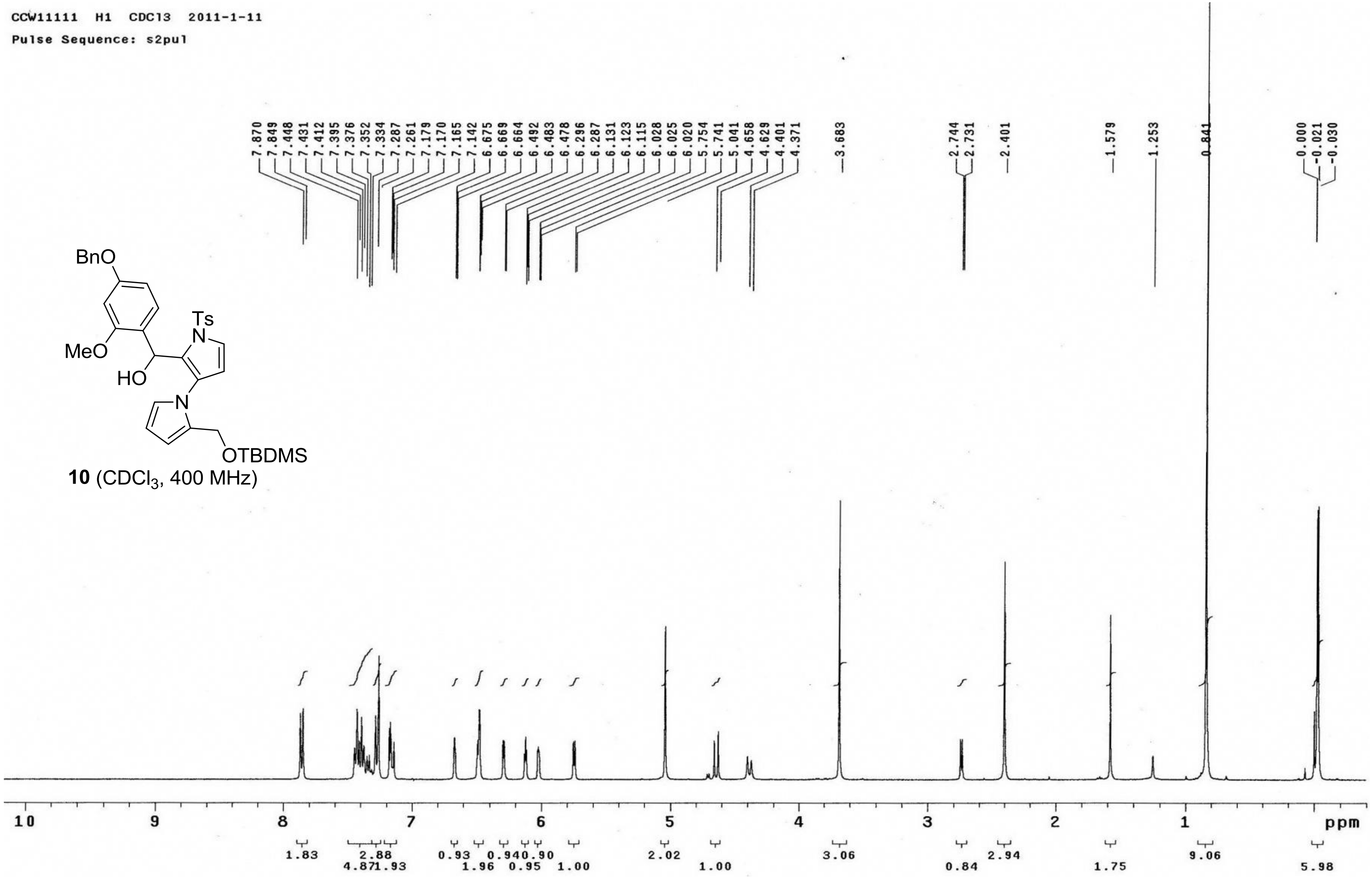

Figure S6. ^{13}C NMR spectrum of **10**.

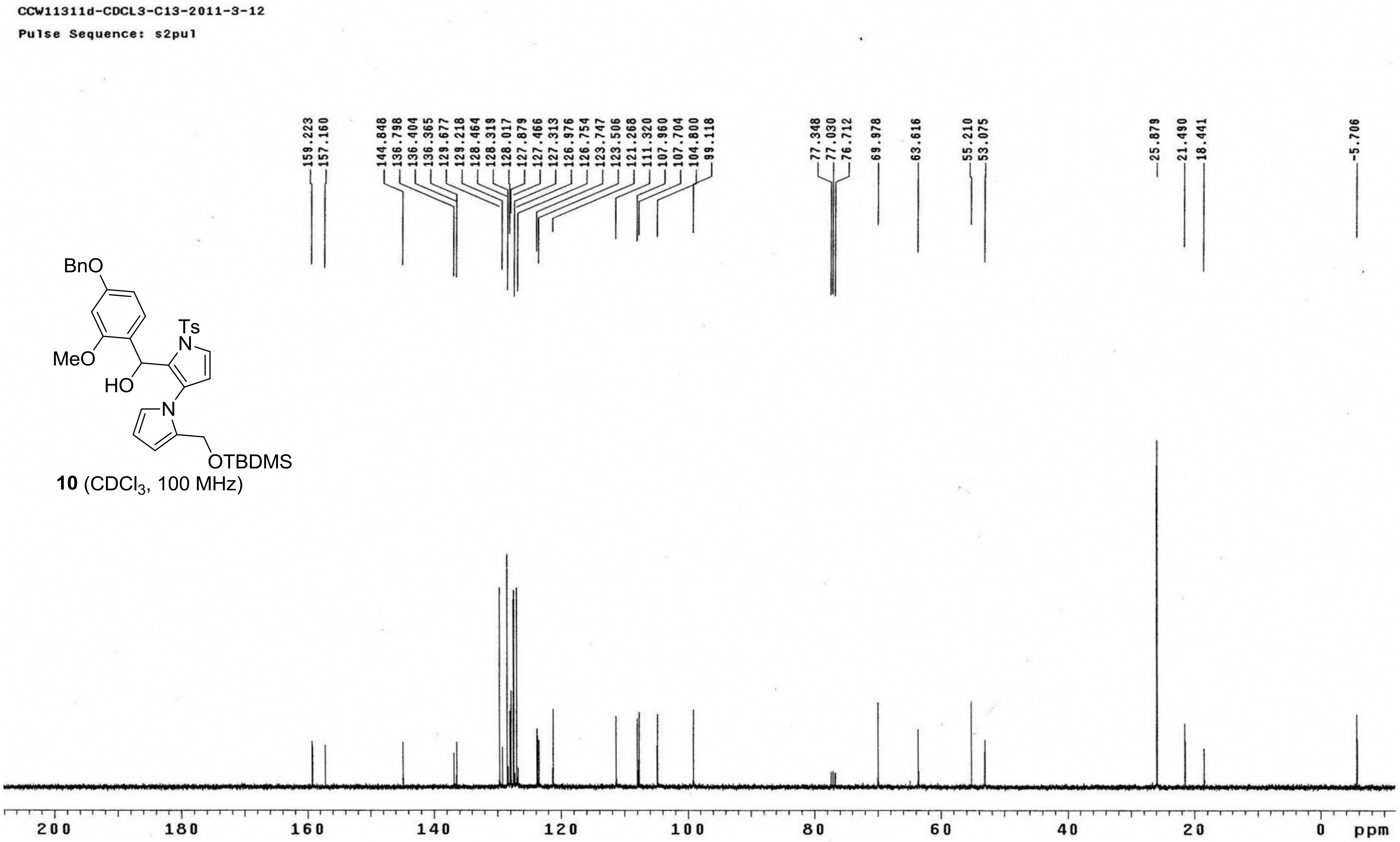

10 ($CDCl_3$, 100 MHz)

Figure S7. ^{1}H NMR spectrum of **11**.

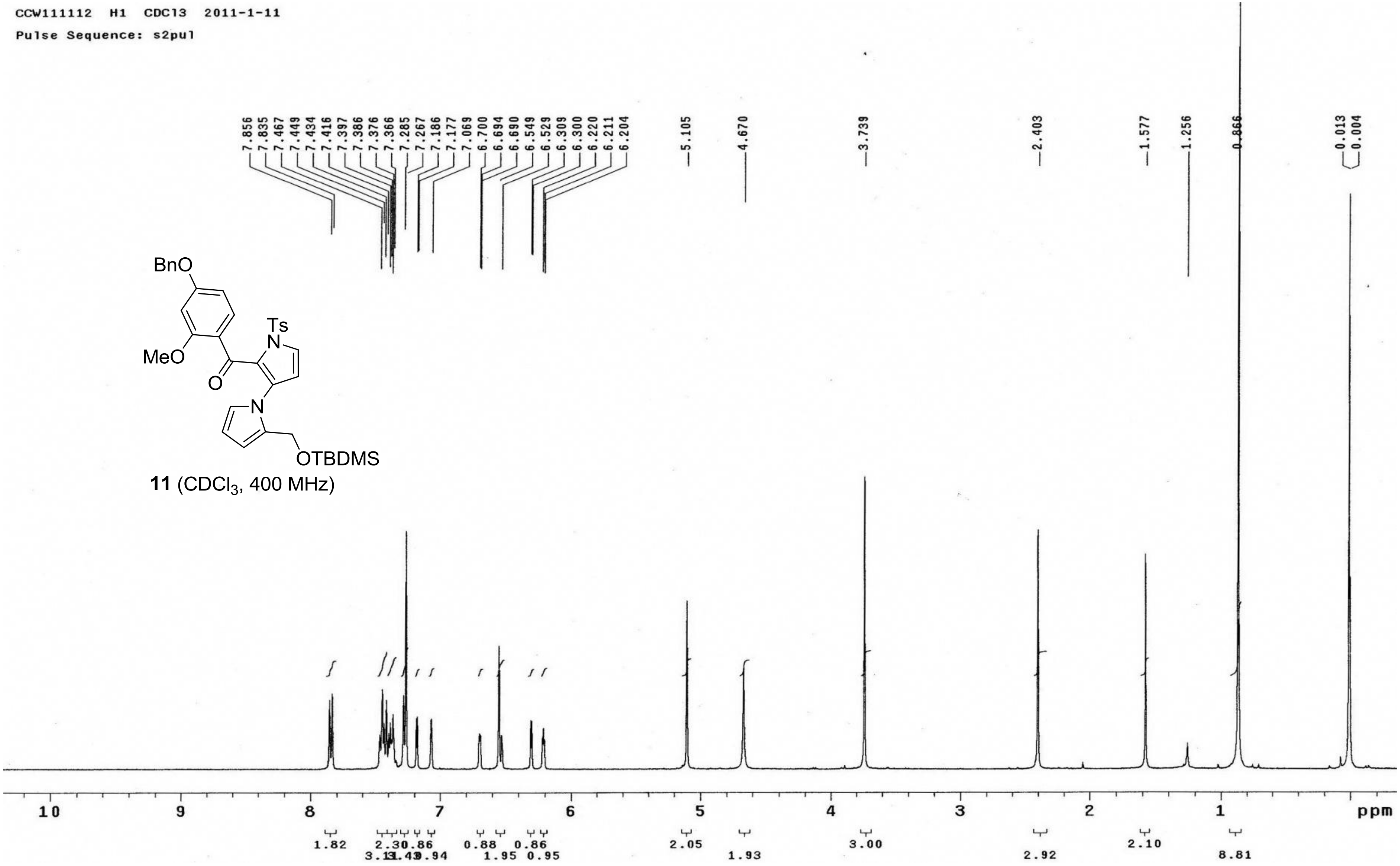

Figure S8. ^{13}C NMR spectrum of **11**.

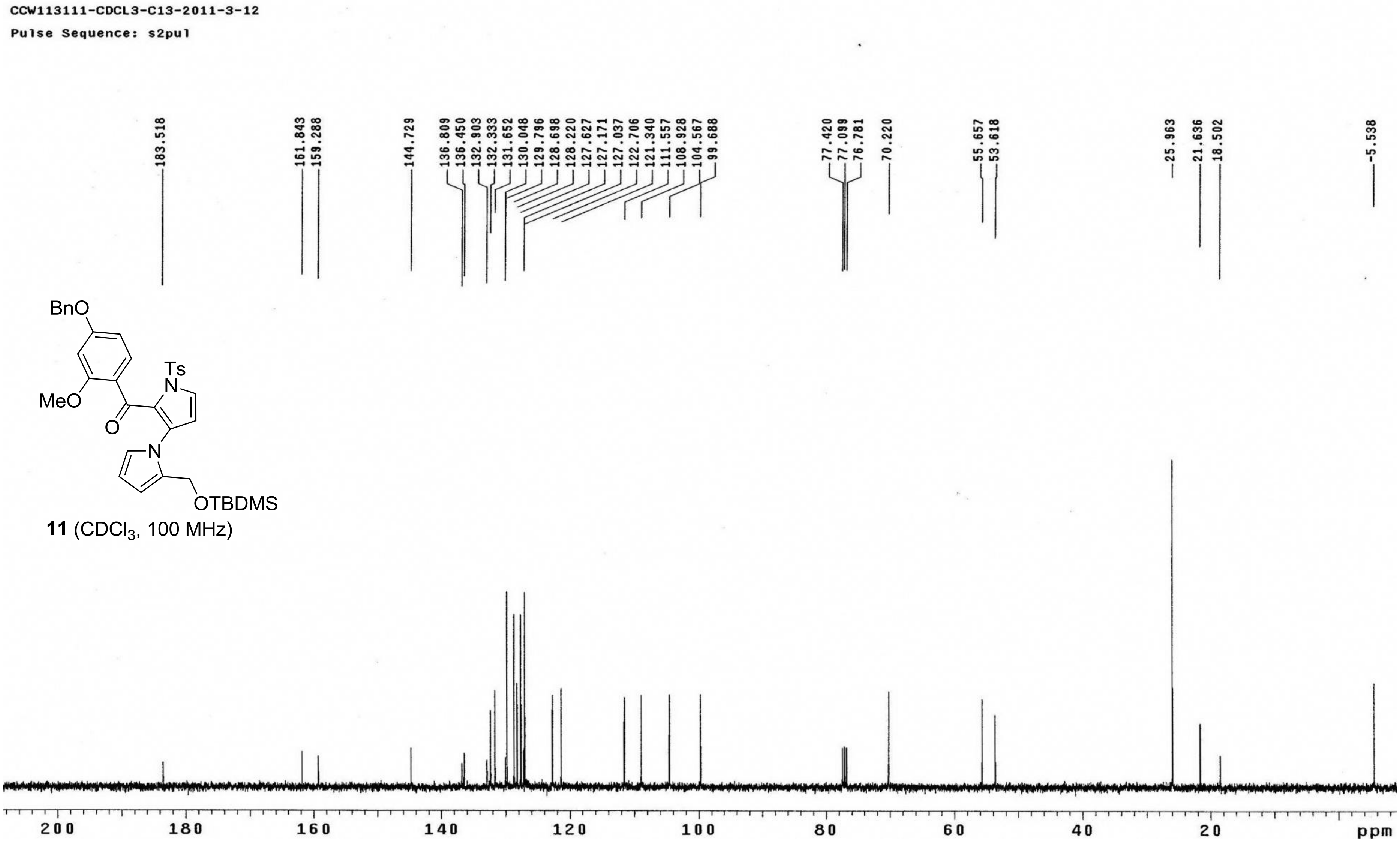

Figure S9. ^{1}H NMR spectrum of **12**.

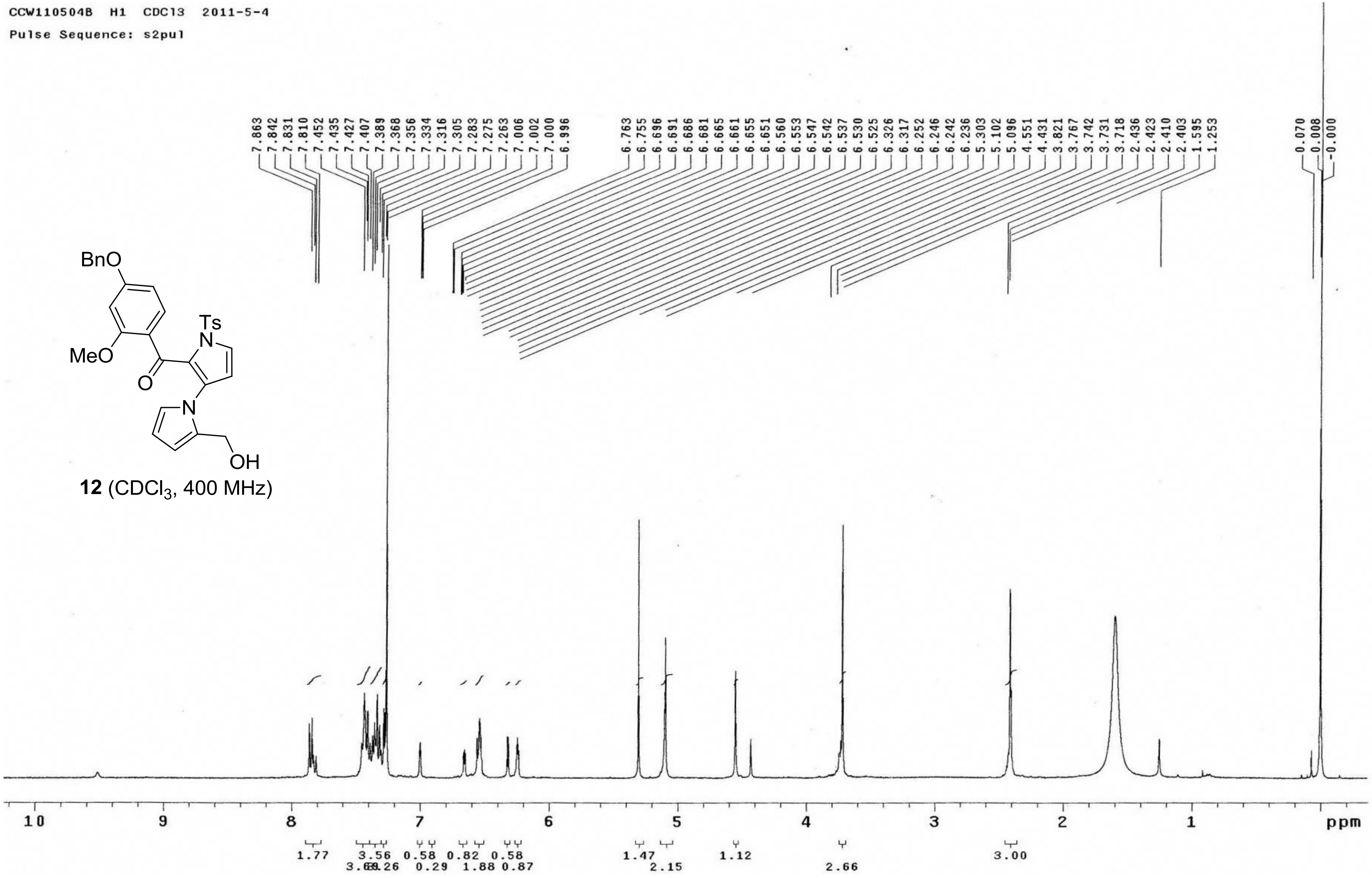

Figure S10. ^{13}C NMR spectrum of **12**.

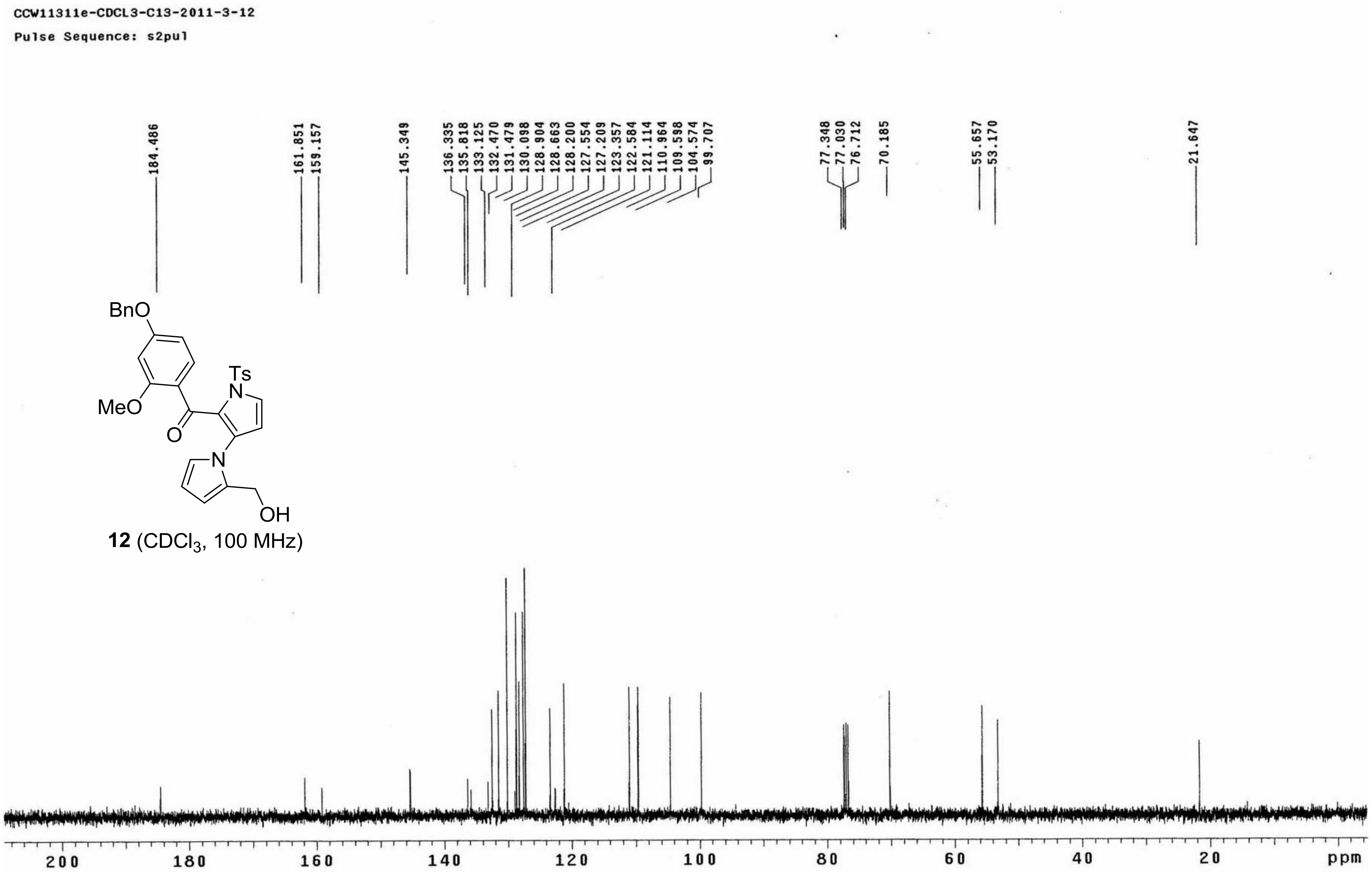

Figure S11. ^{1}H NMR spectrum of **13**.

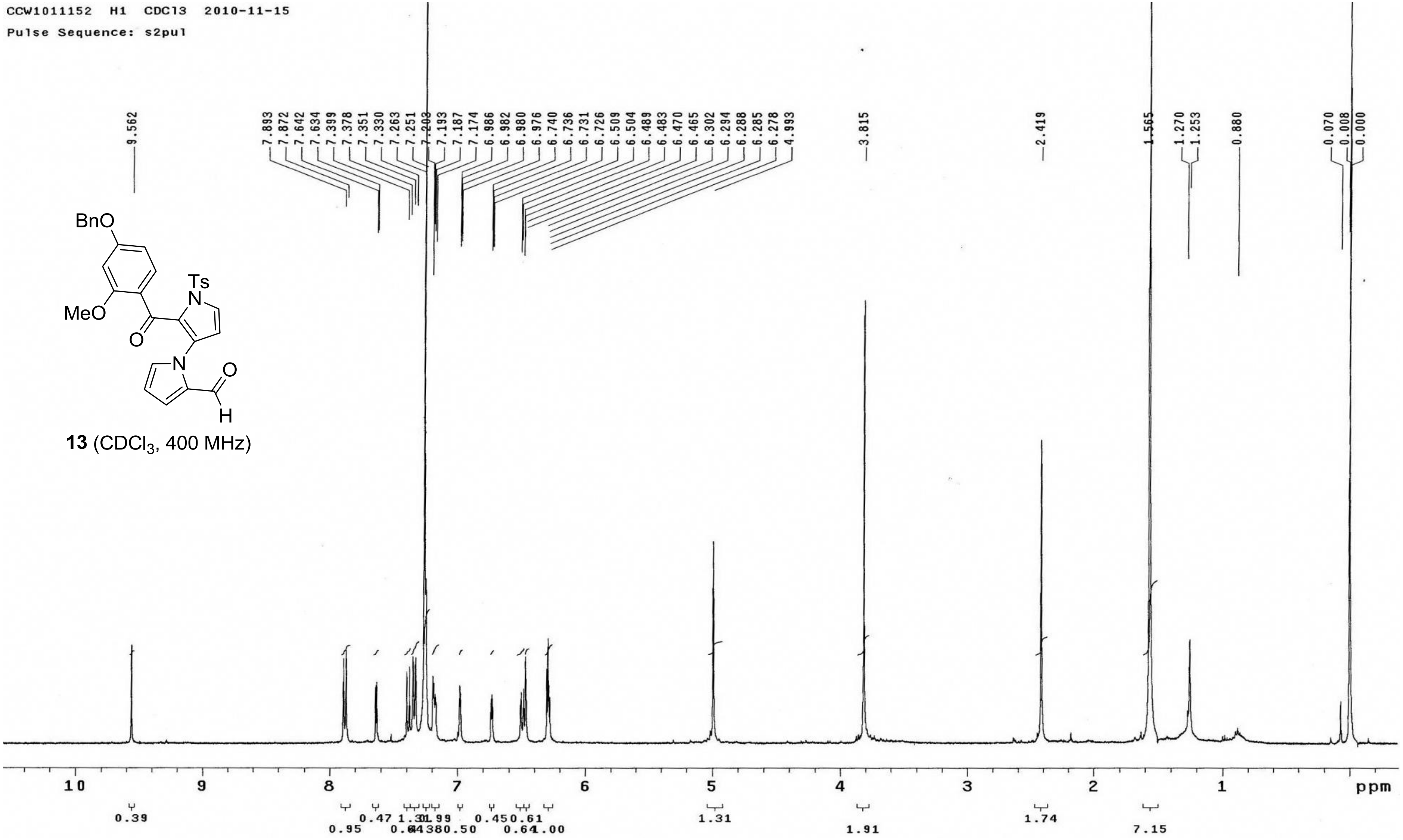

Figure S12. ^{13}C NMR spectrum of **13**.

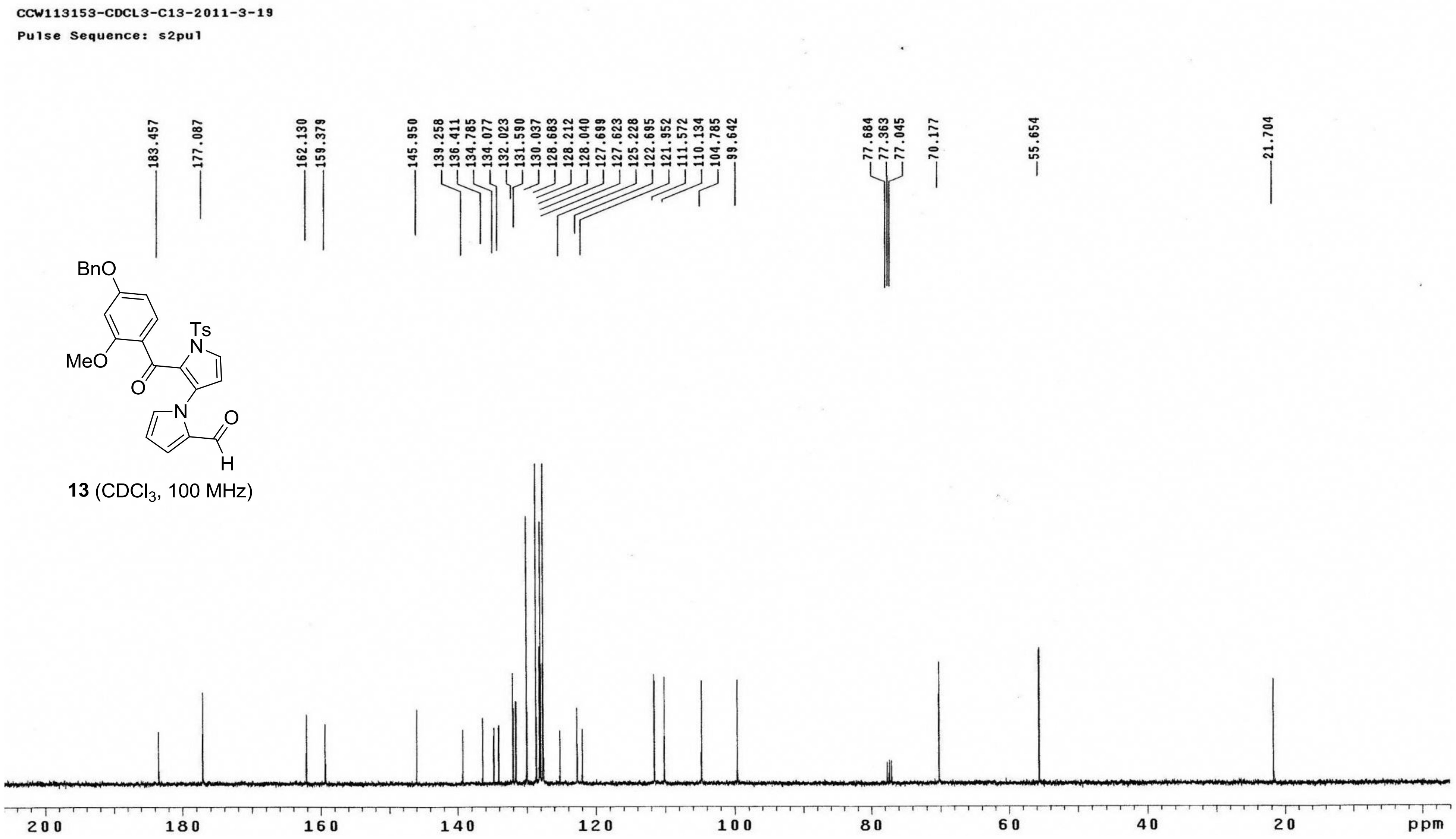

Figure S13. ^{1}H NMR spectrum of **14**.

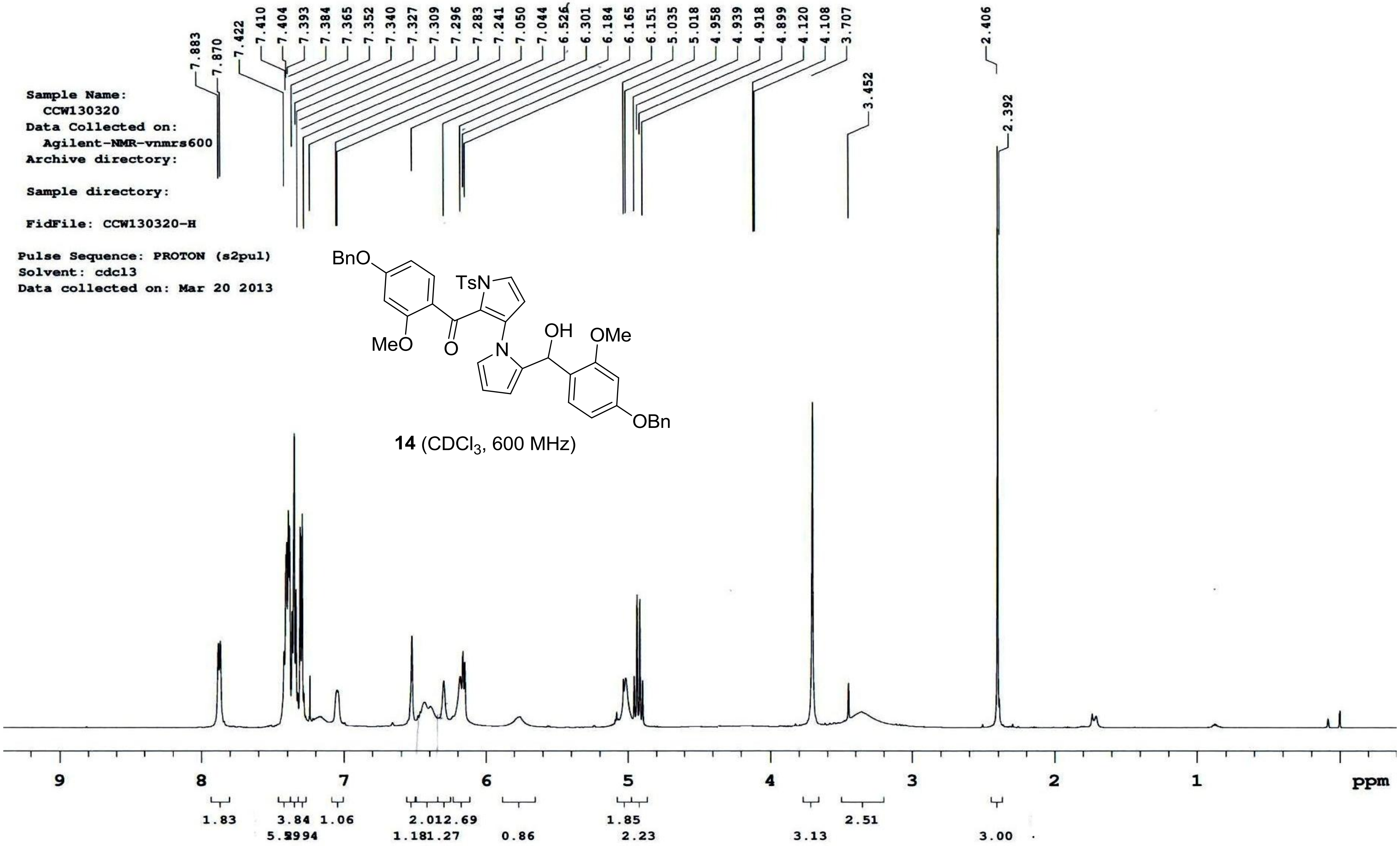

Figure S14. ^{13}C NMR spectrum of **14**.

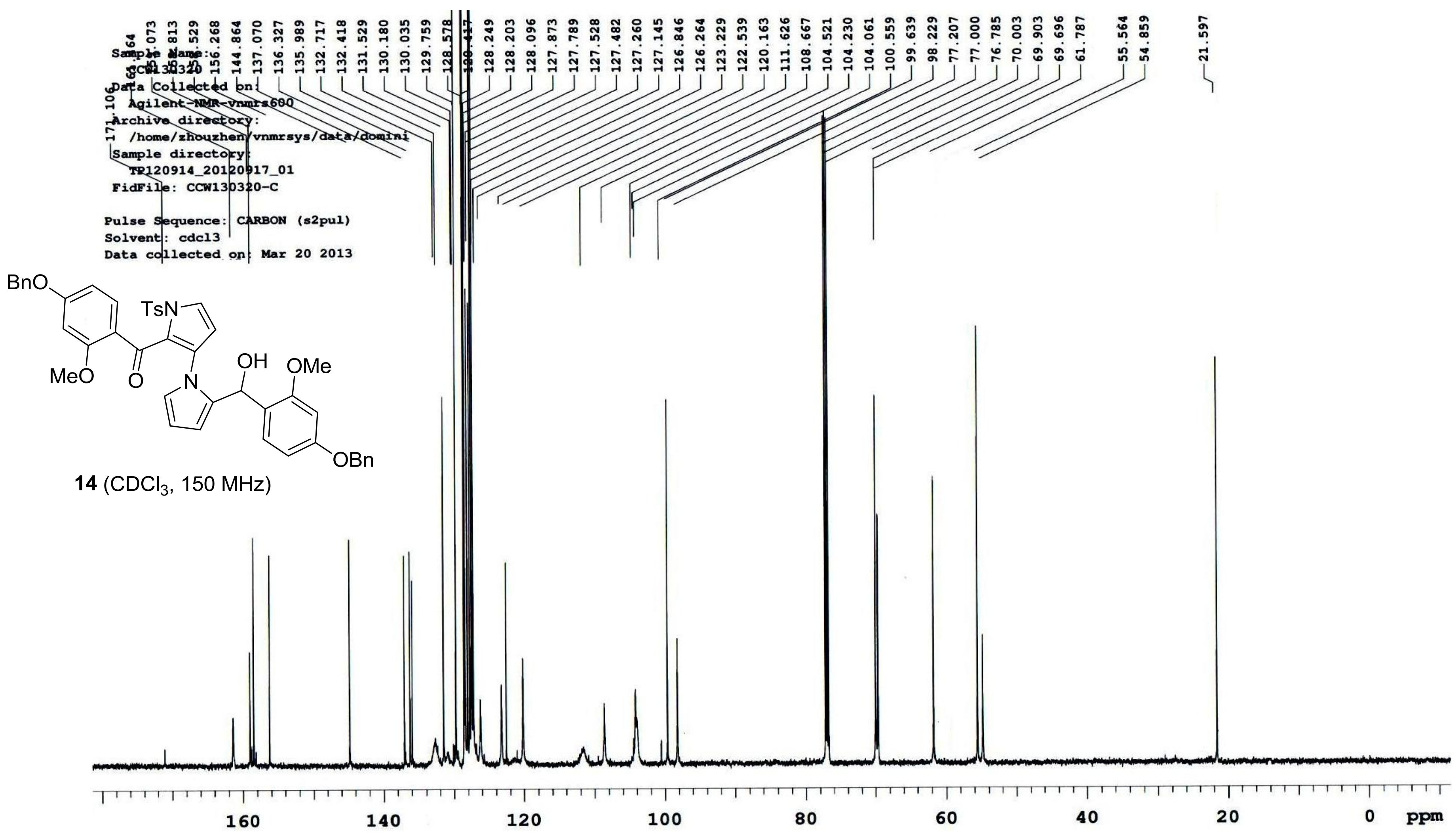

Figure S15. ^{1}H NMR spectrum of **14A**.

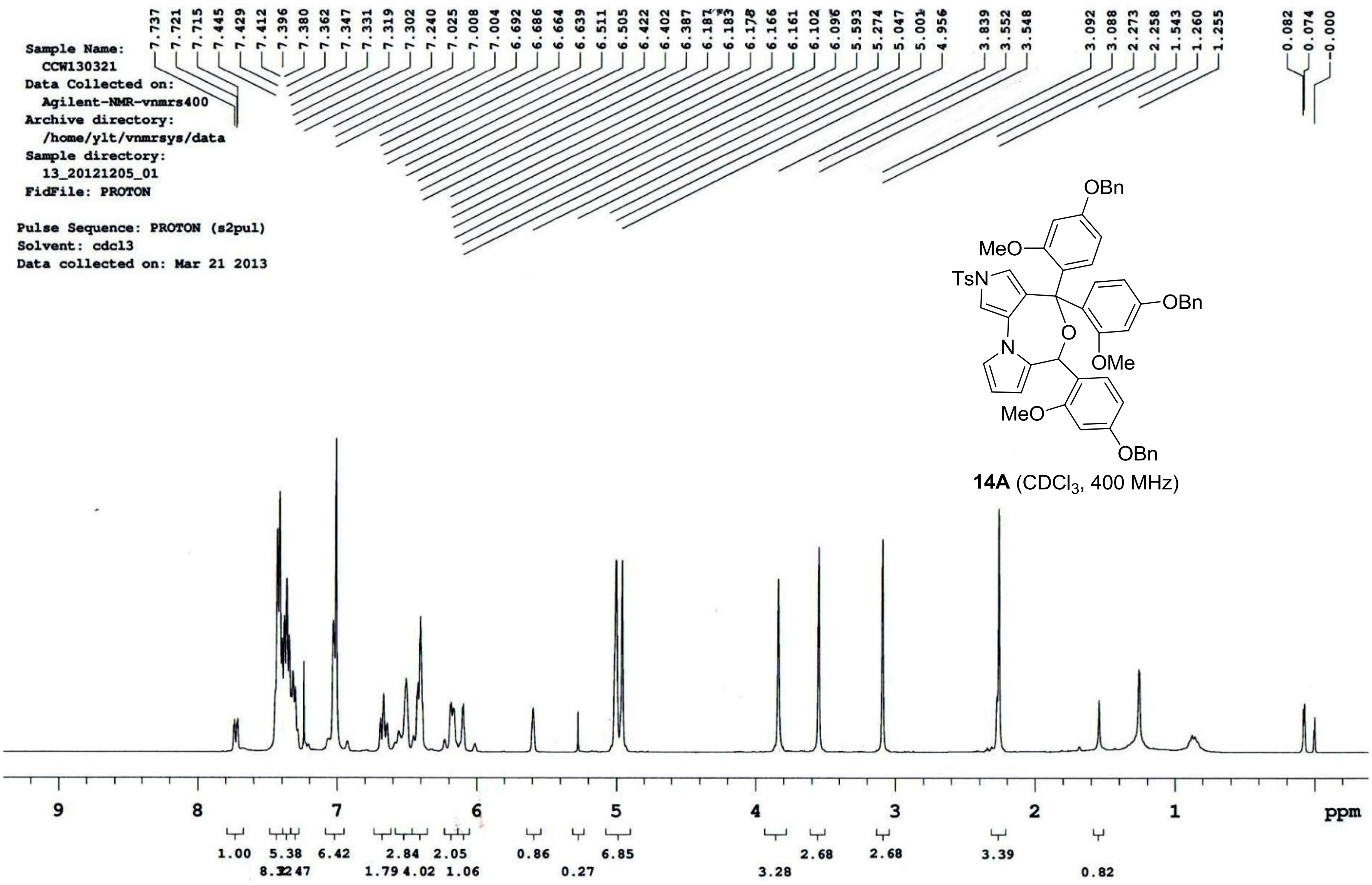

Figure S16. ^{13}C NMR spectrum of **14A**.

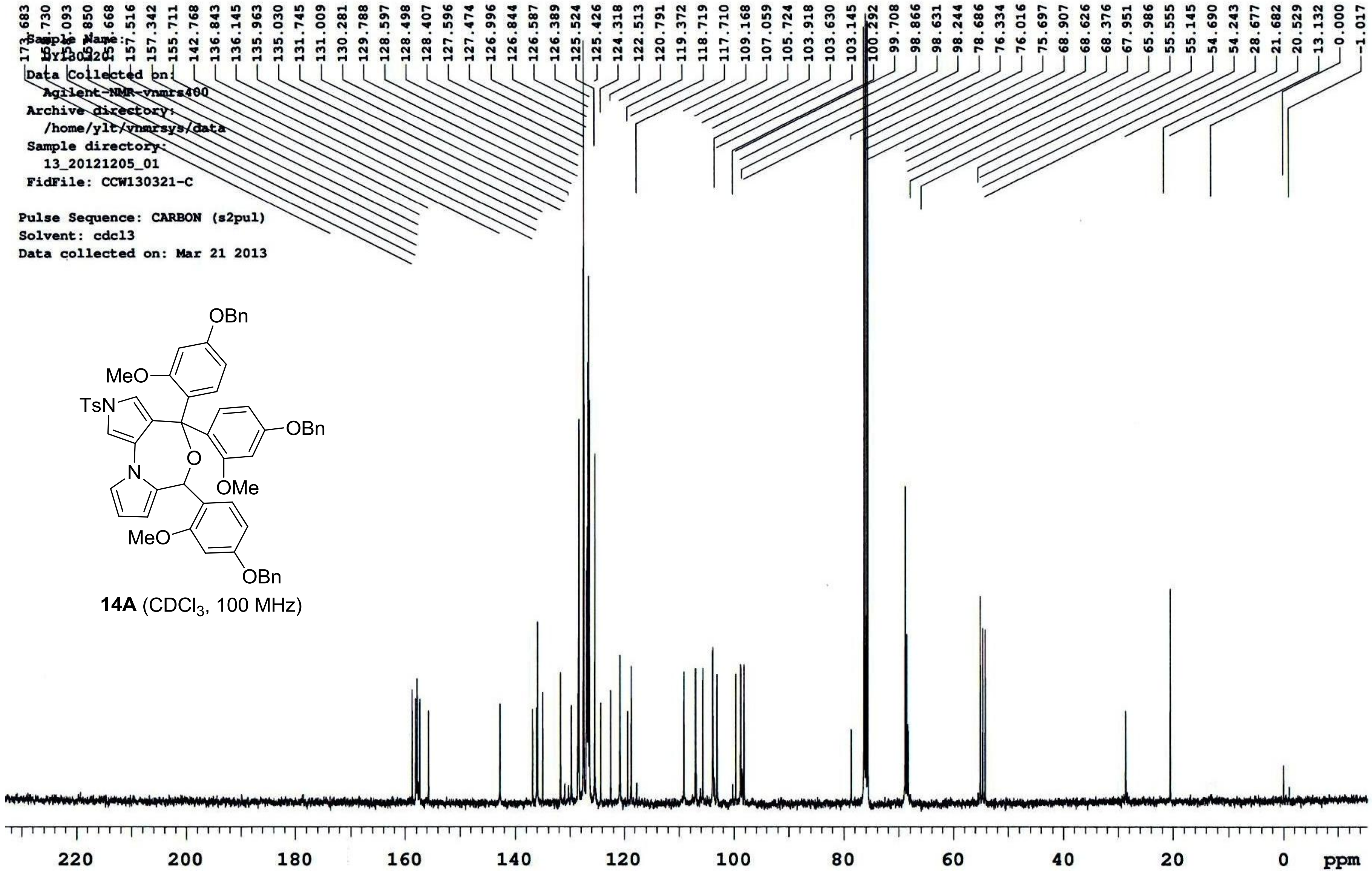

Figure S17. ^{1}H NMR spectrum of **15**.

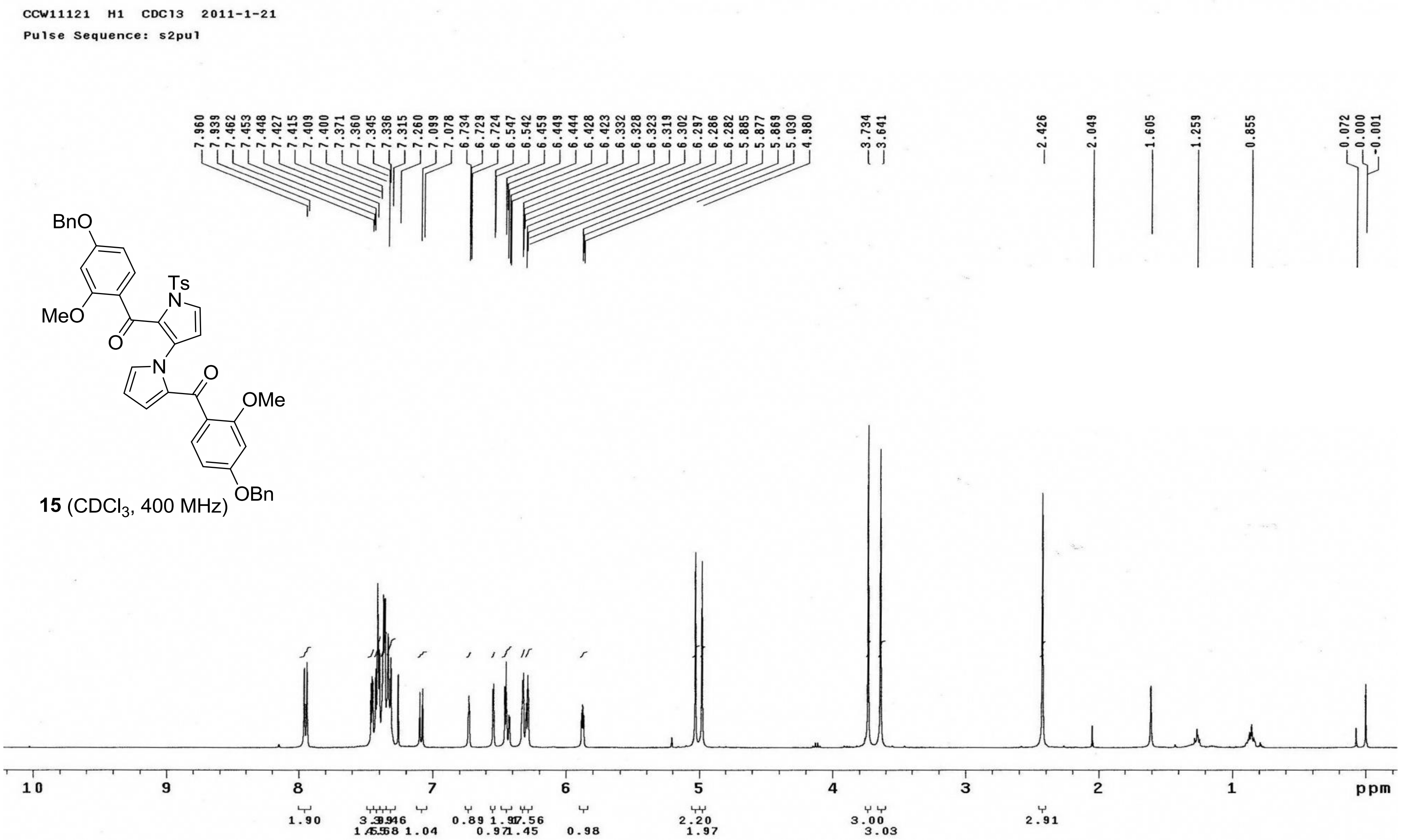

Figure S18. ^{13}C NMR spectrum of **15**.

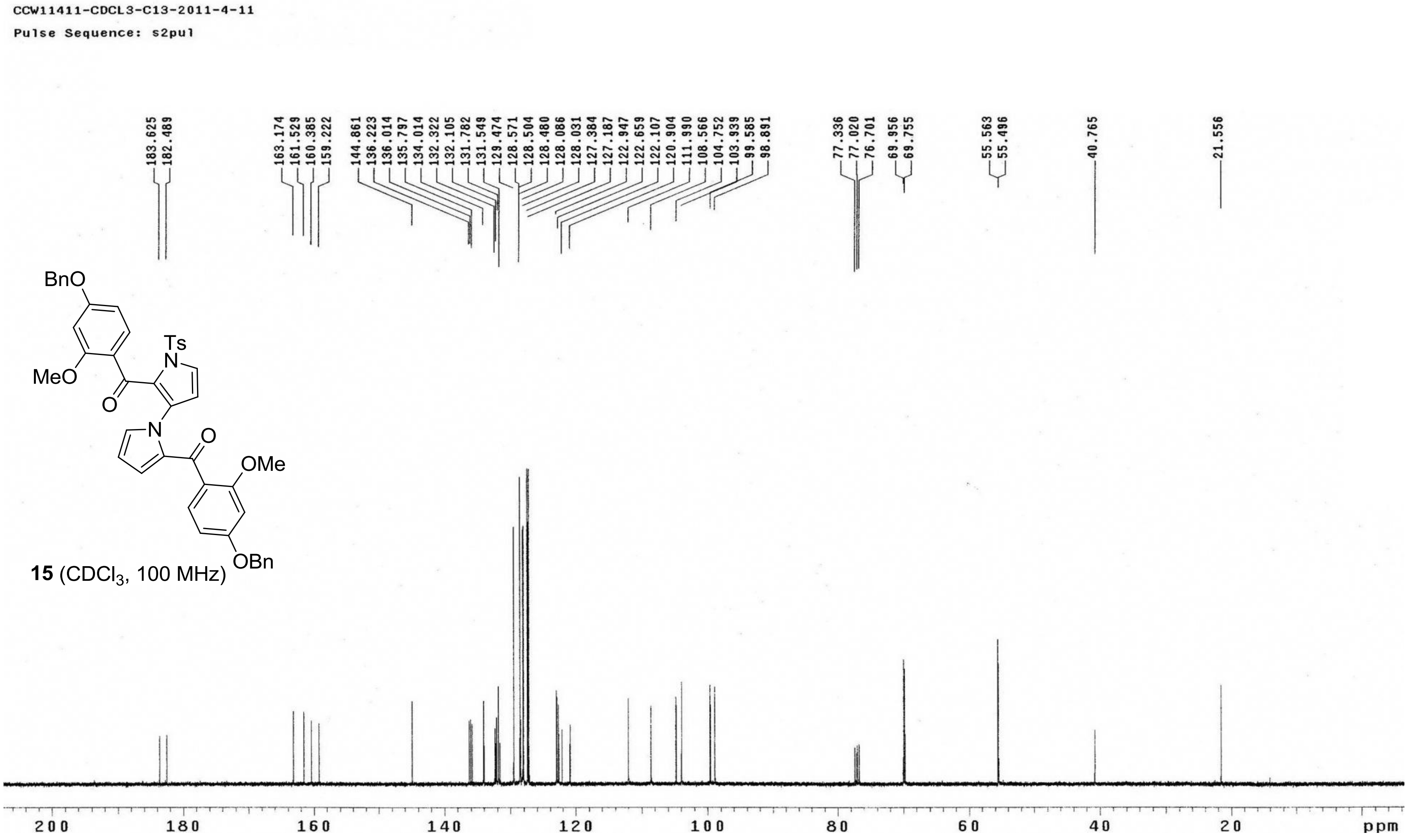

Figure S19. ^{1}H NMR spectrum of **16**.

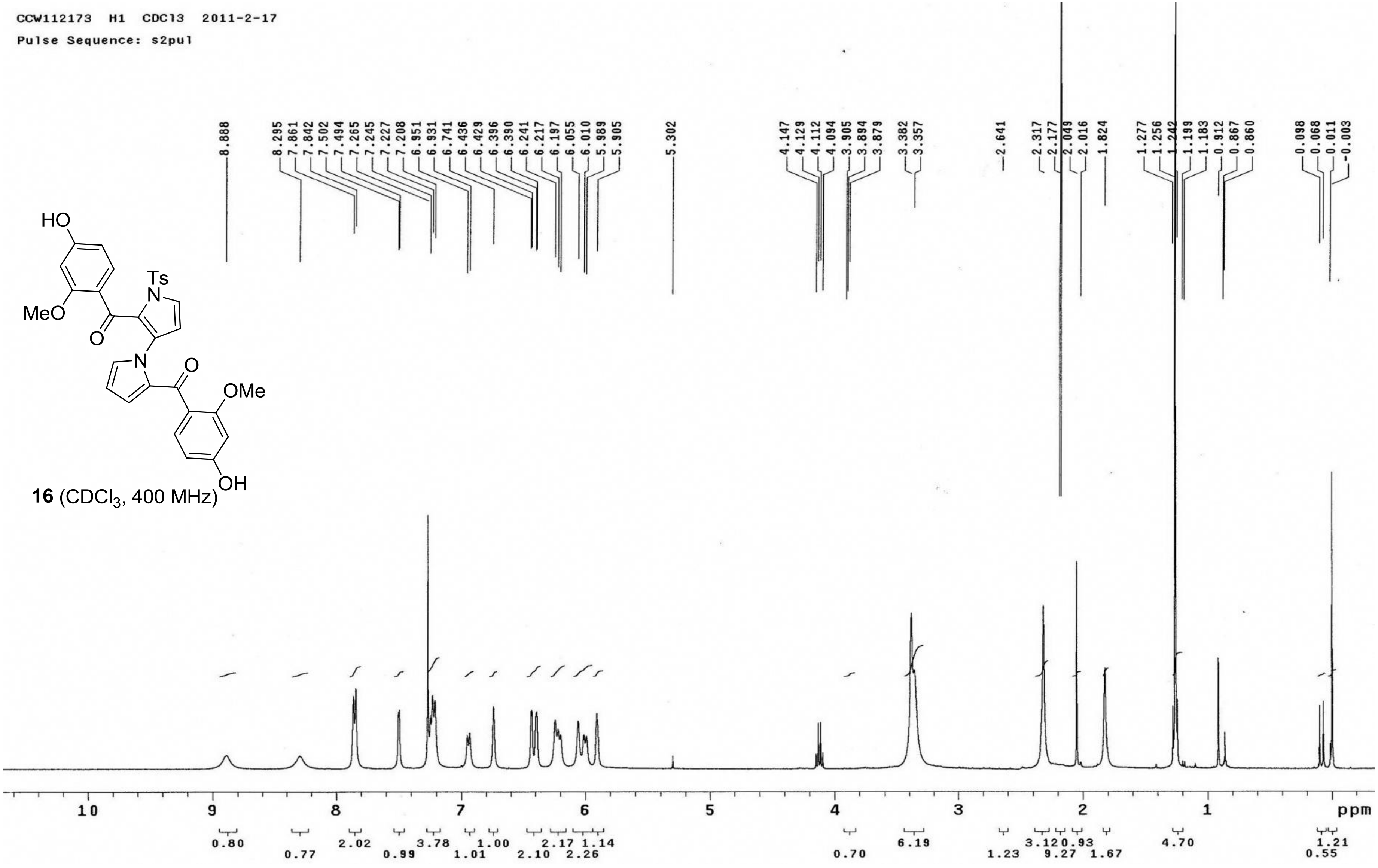

Figure S20. ^{13}C NMR spectrum of **16**.

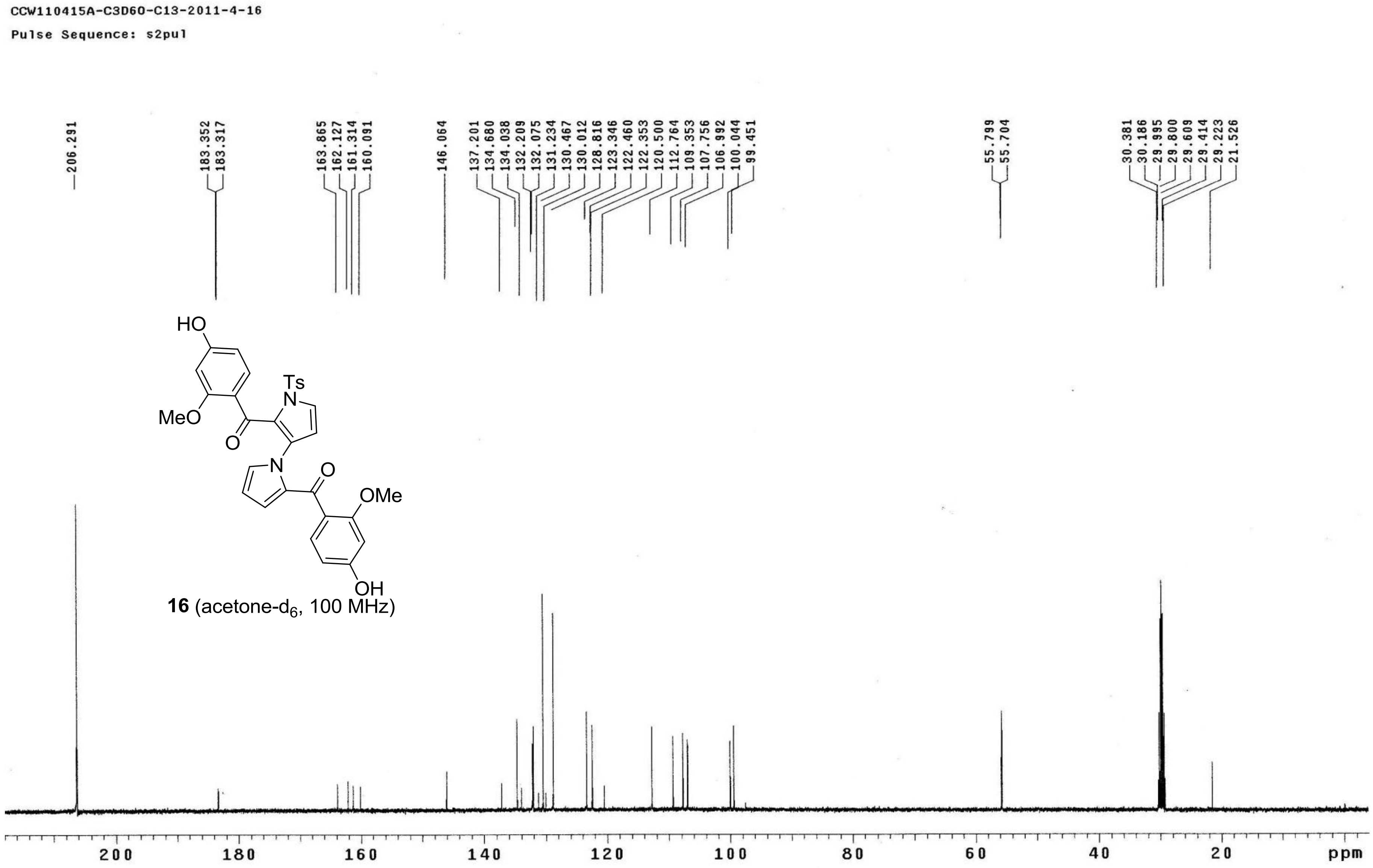

Figure S21. ^{1}H NMR spectrum of **17**.

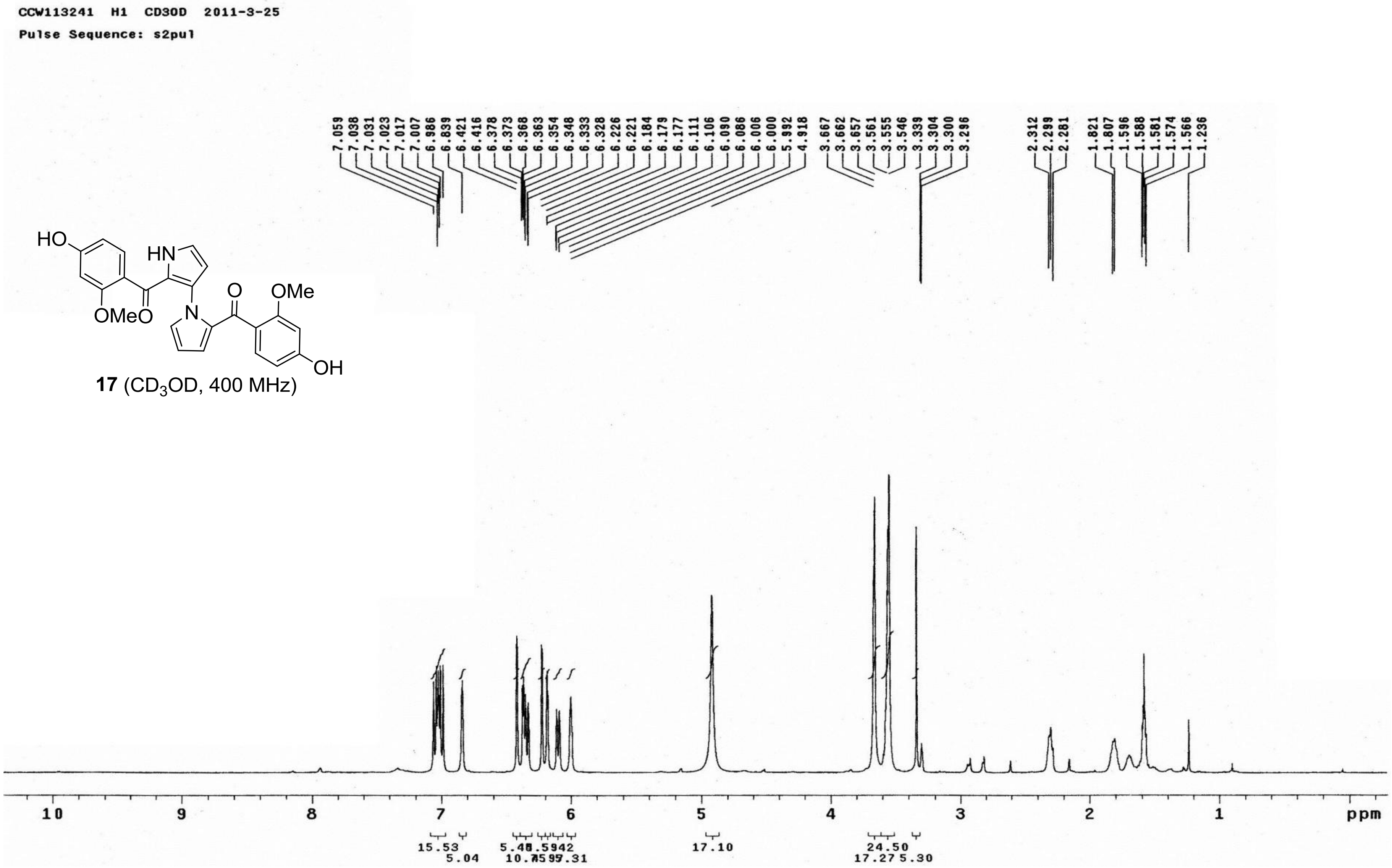

Figure S22. ^{13}C NMR spectrum of **17**.

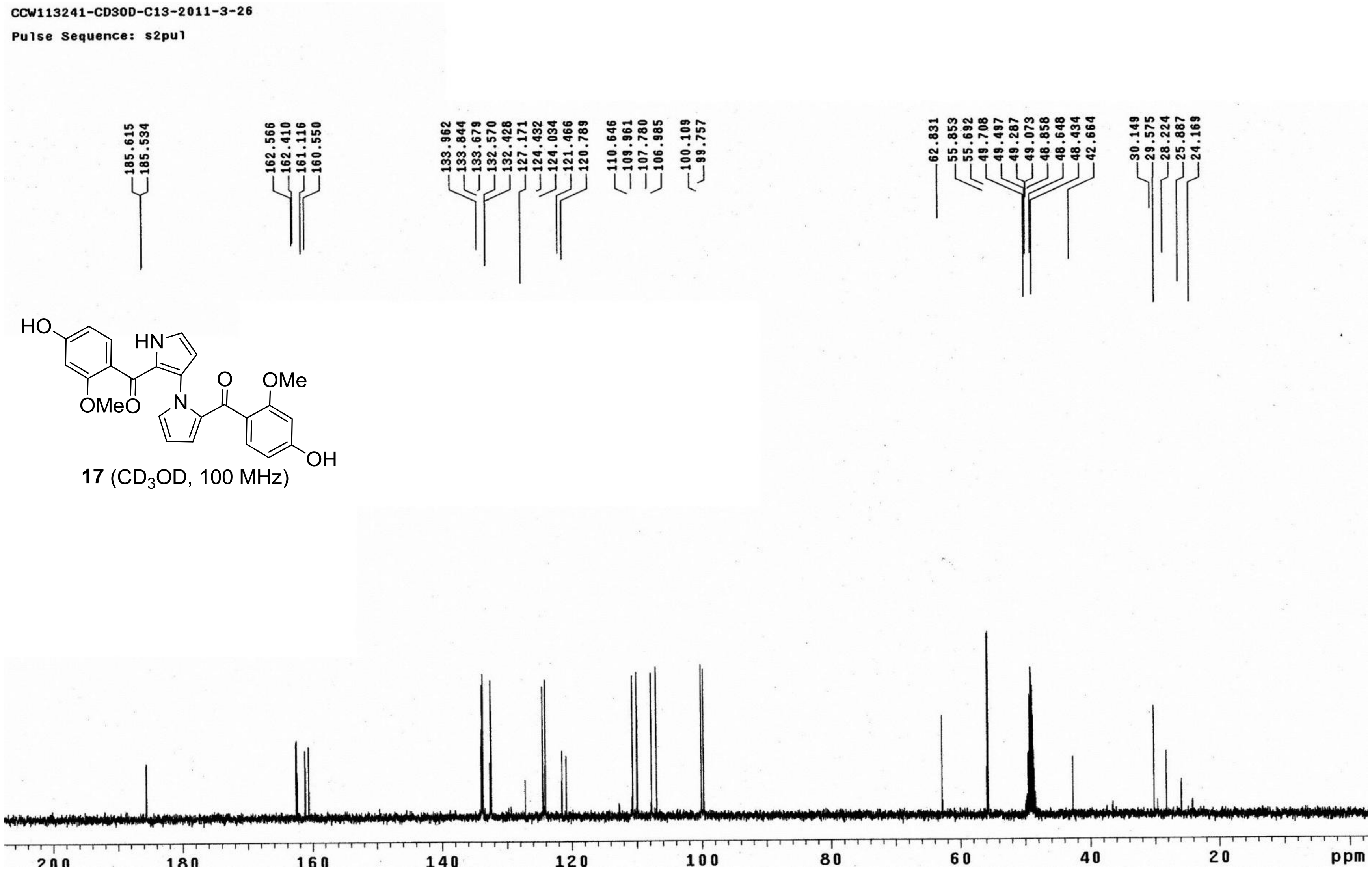

Figure S23. ^{1}H NMR spectrum of **18**.

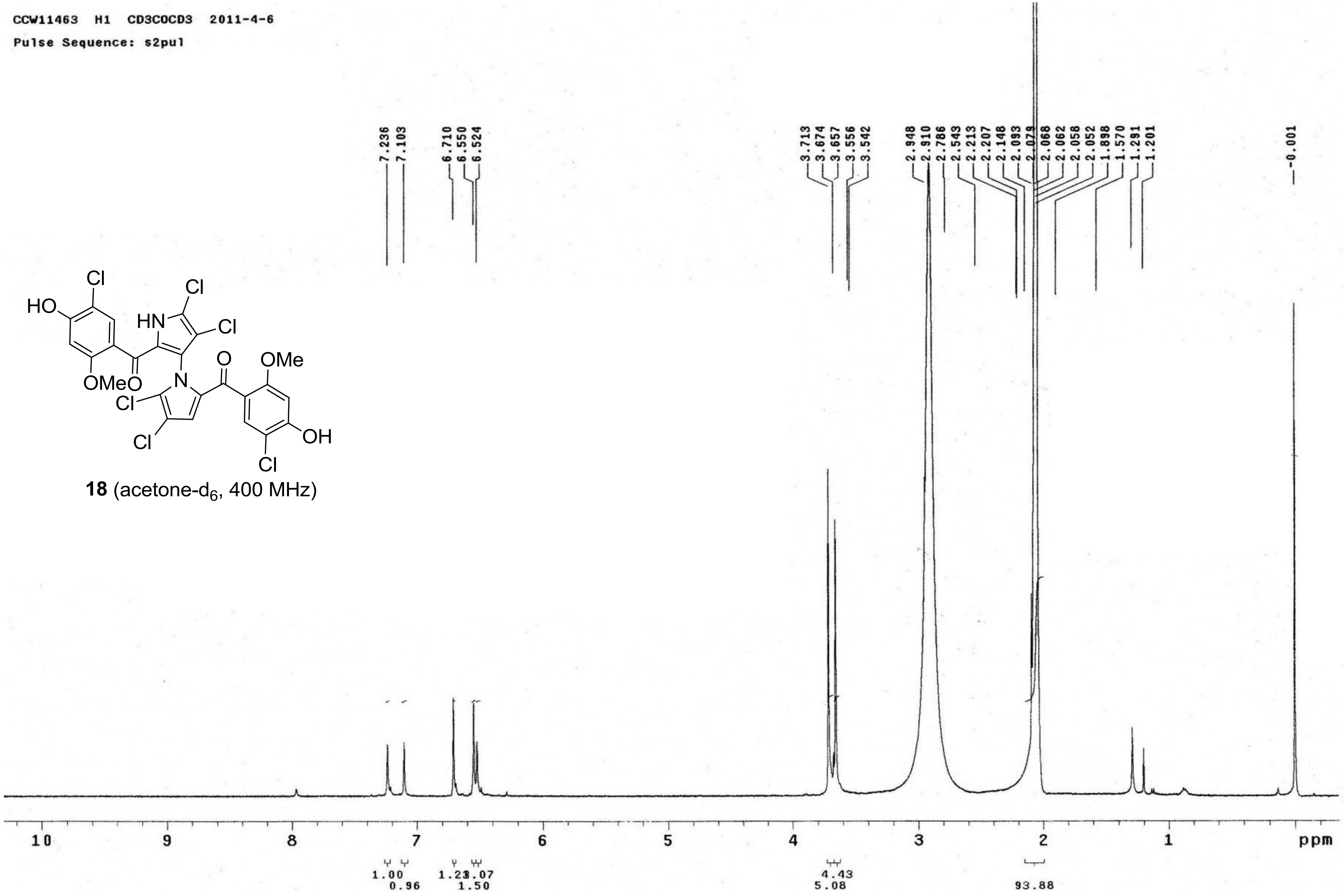

Figure S24. ^{13}C NMR spectrum of **18**.

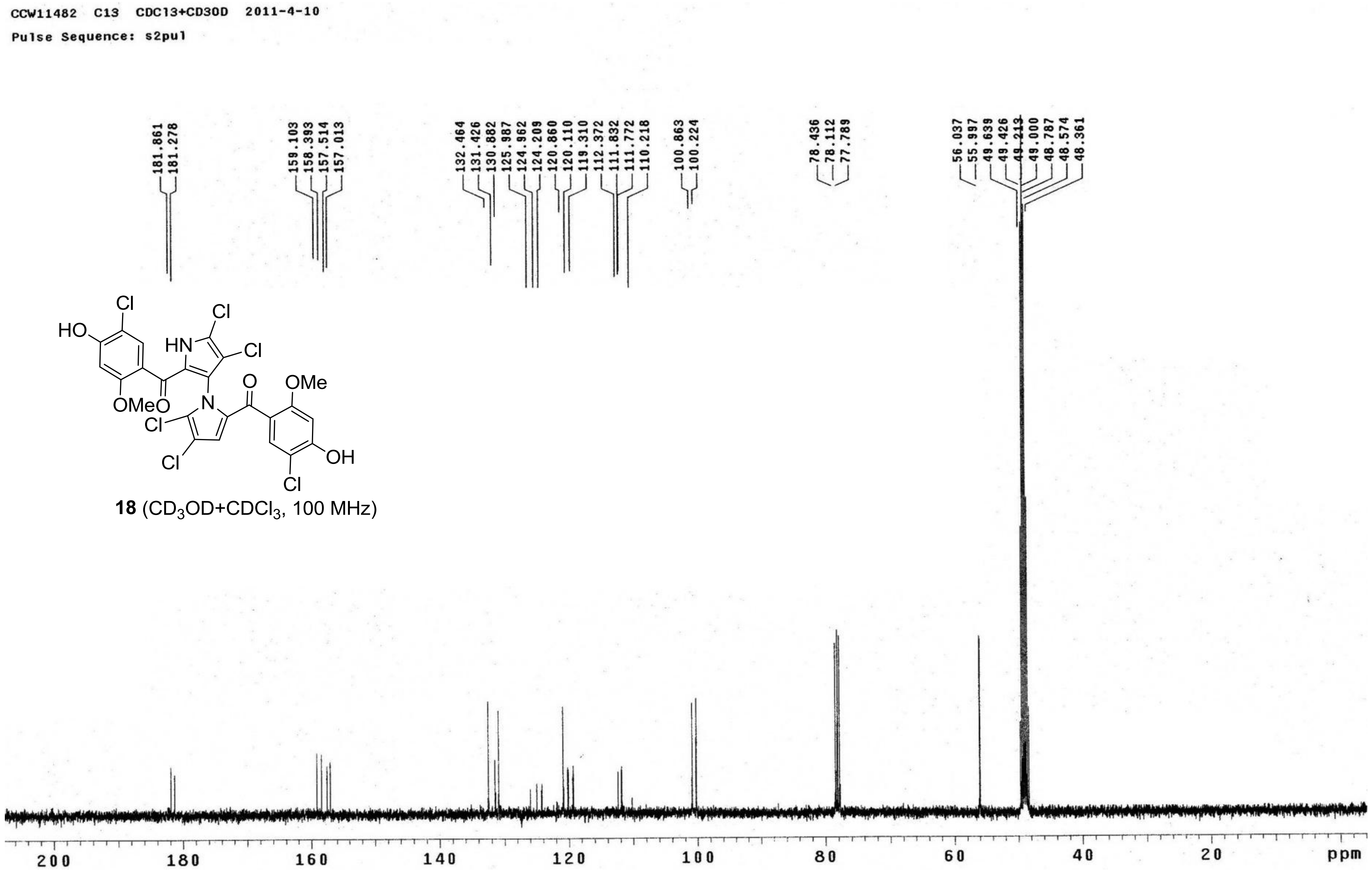

Figure S25. ^{1}H NMR spectrum of **19**.

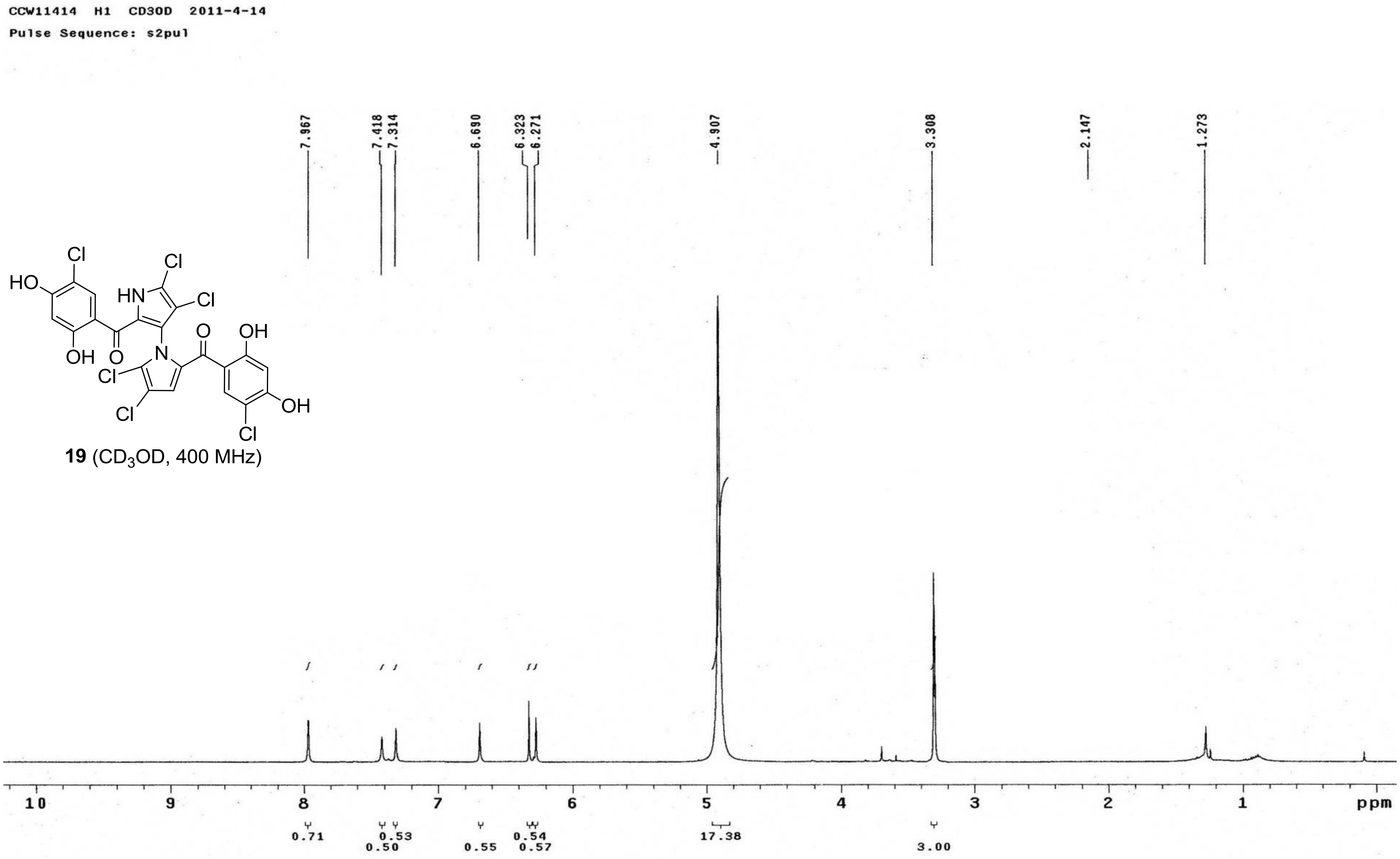

Figure S26. ^{13}C NMR spectrum of **19**.

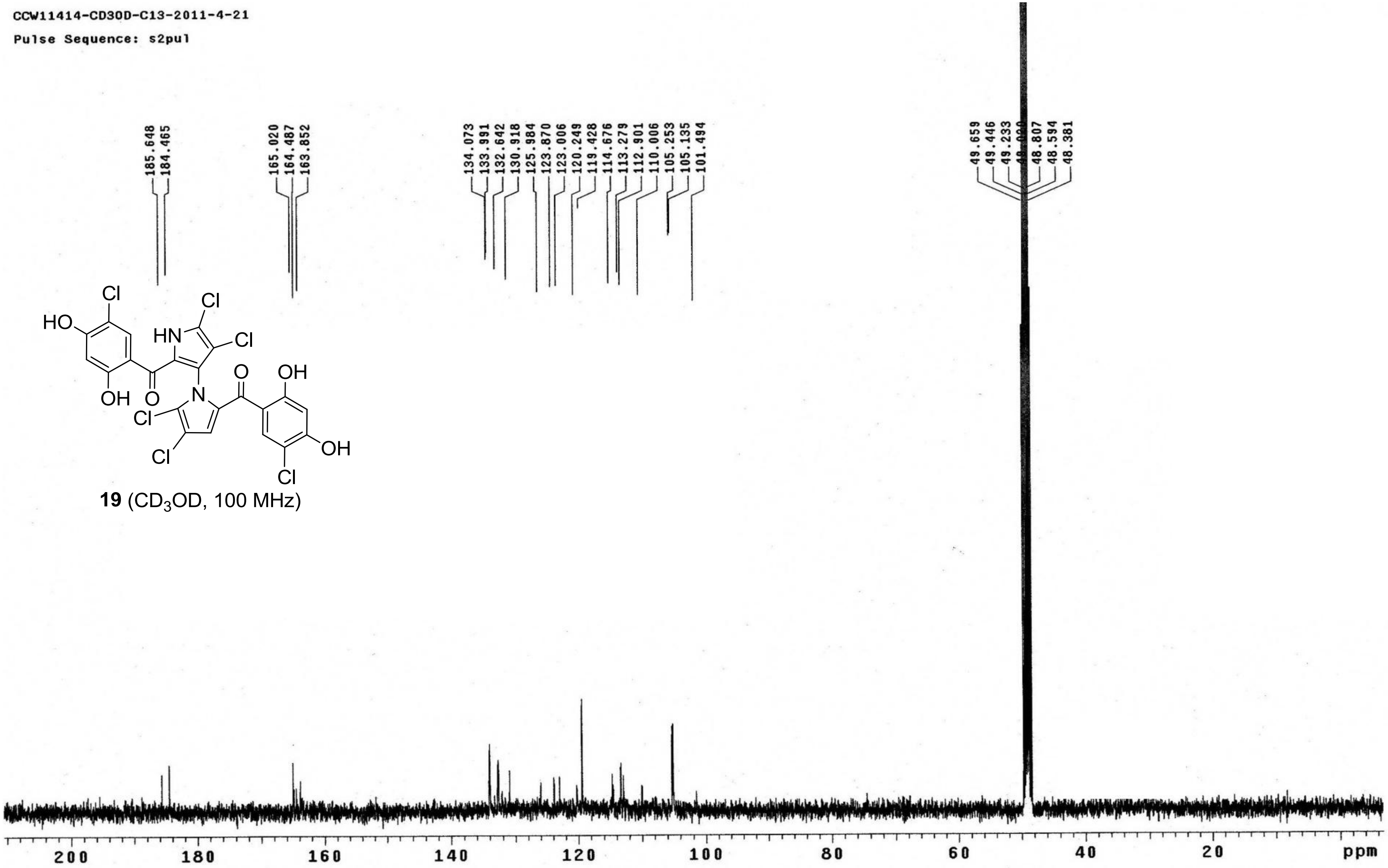

Figure S27. ^{1}H NMR spectrum of **20**.

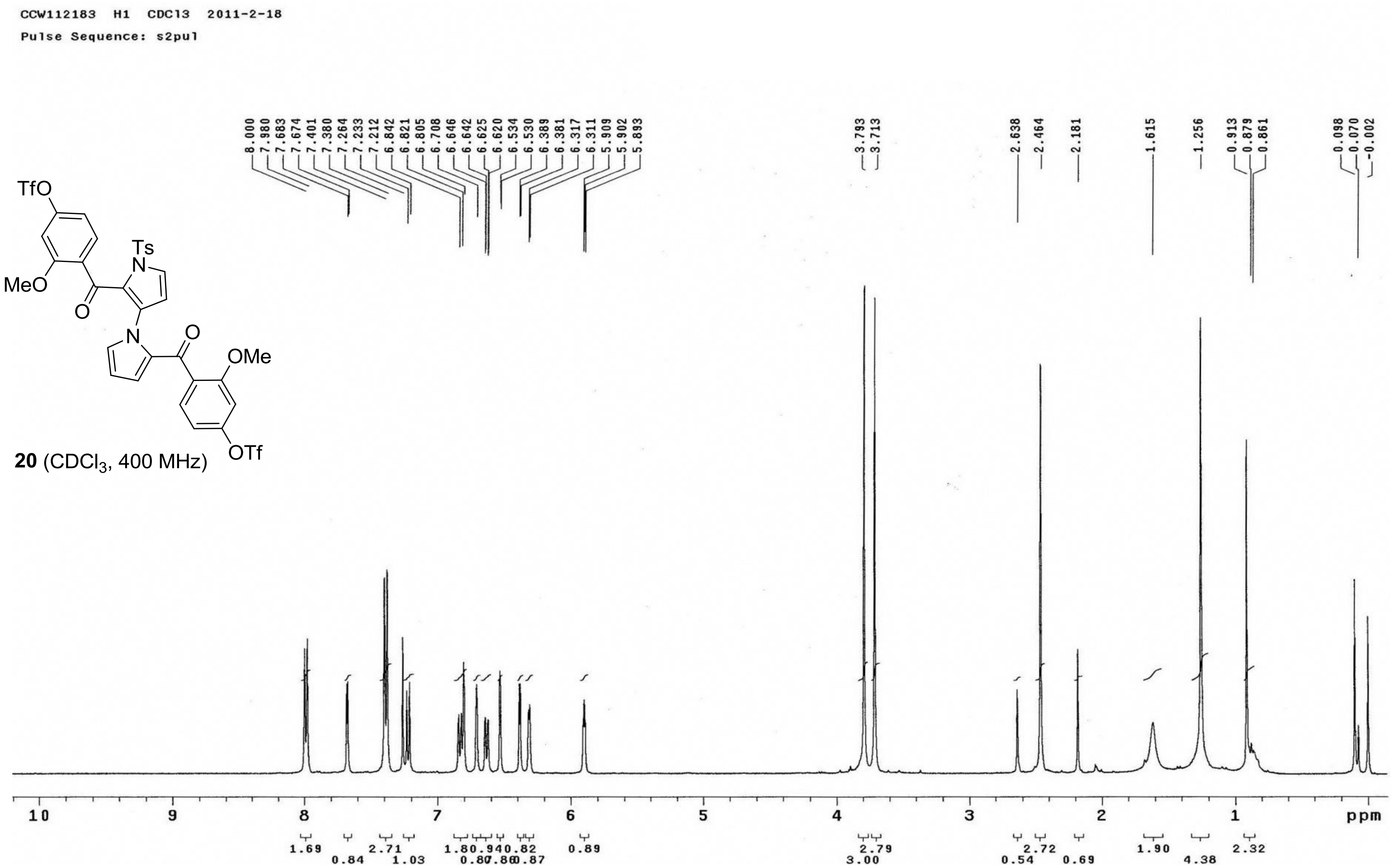

Figure S28. ^{13}C NMR spectrum of **20**.

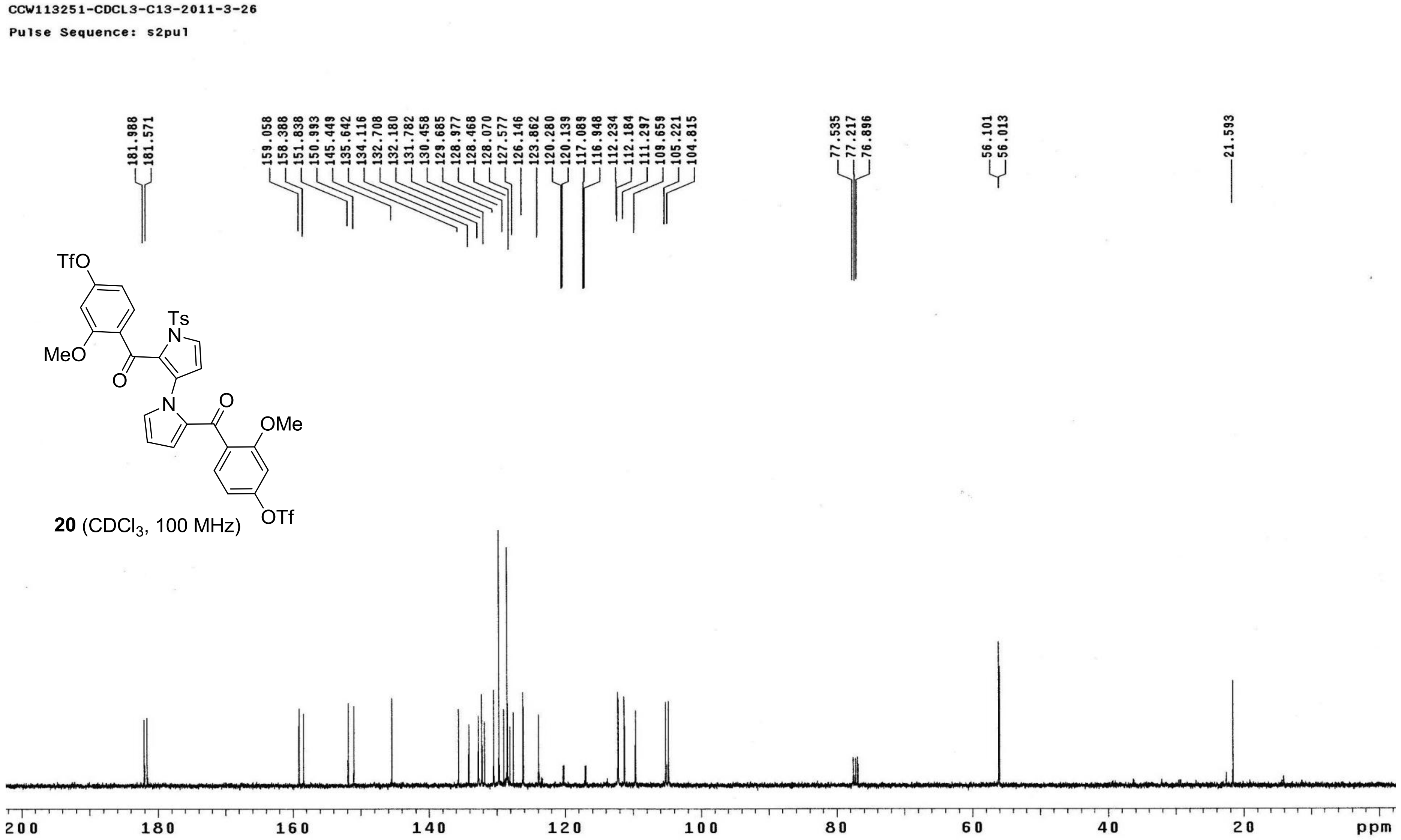

Figure S29. ^{1}H NMR spectrum of **21**.

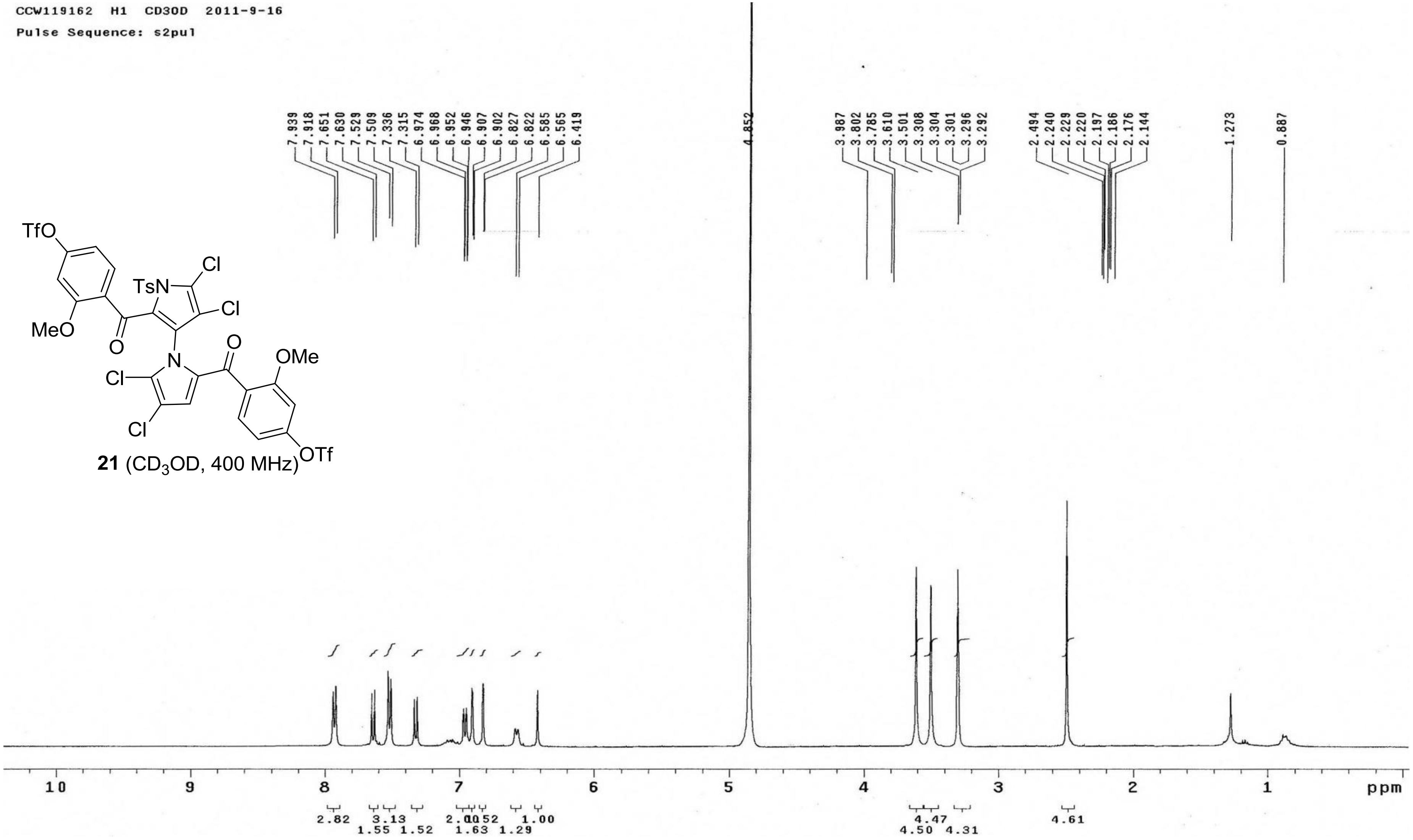

Figure S30. ^{13}C NMR spectrum of **21**.

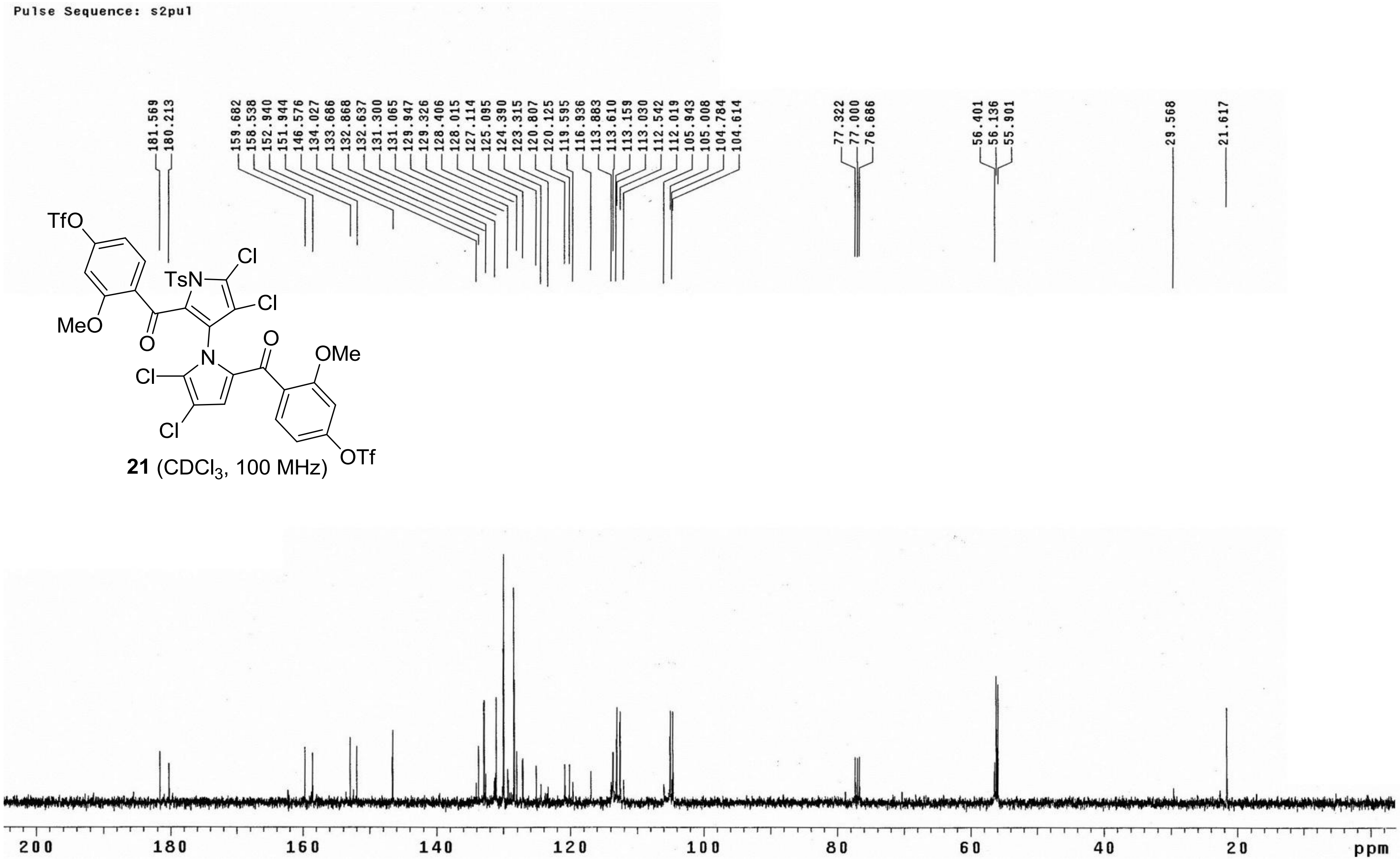

Figure S31. ¹H NMR spectrum of **22**.

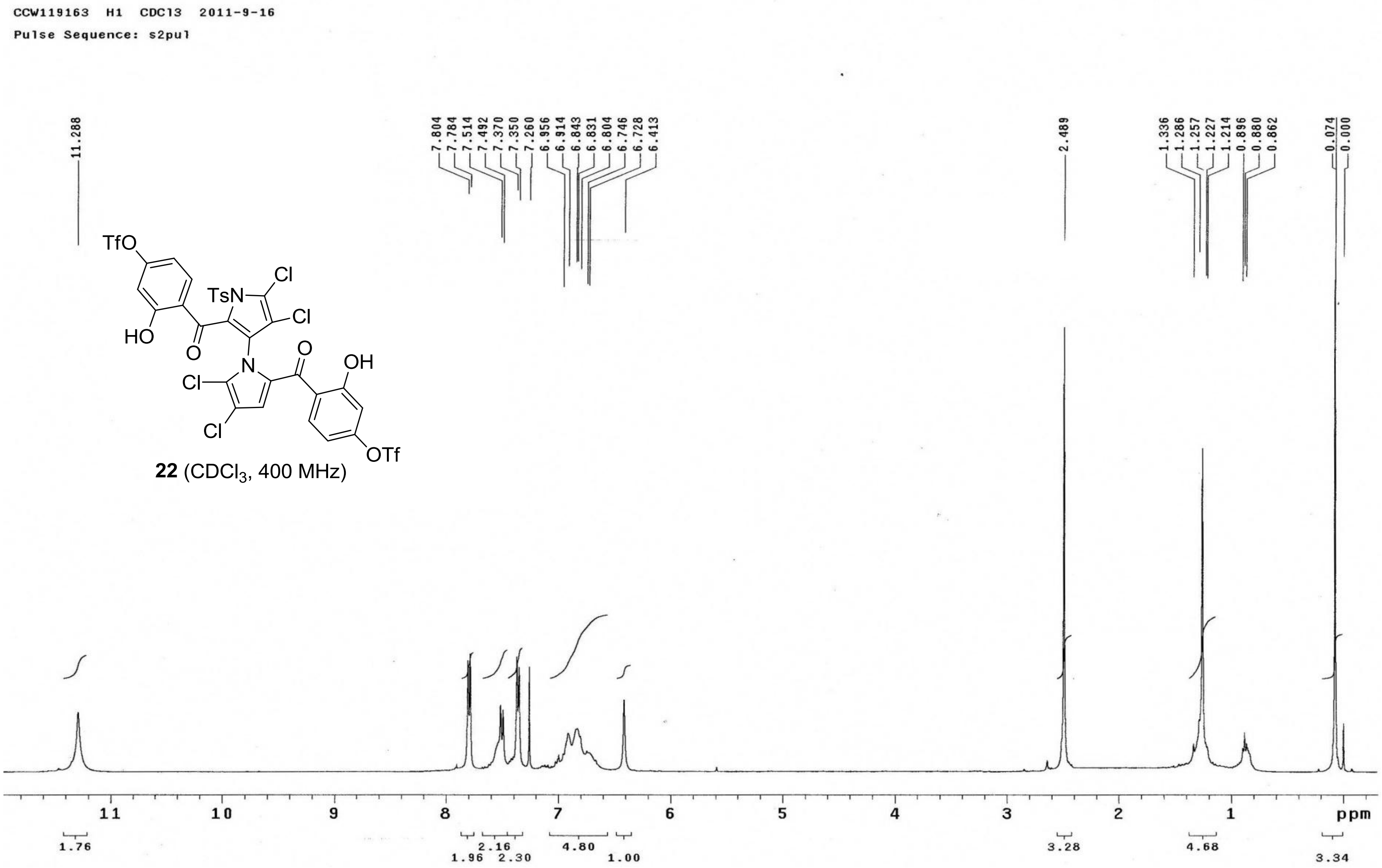

Figure S32. ^{13}C NMR spectrum of **22**.

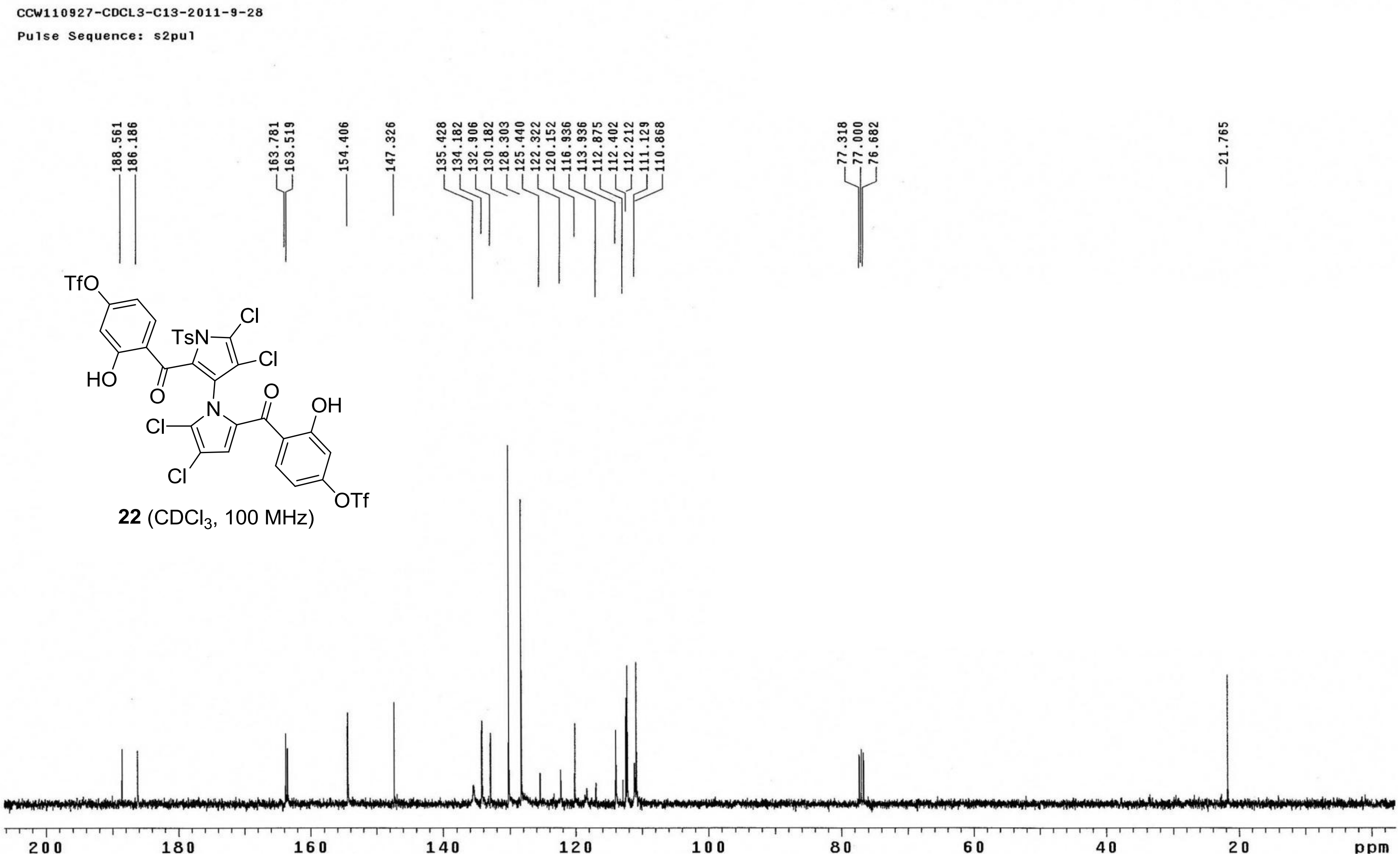

Figure S33. ^{1}H NMR spectrum of **23**.

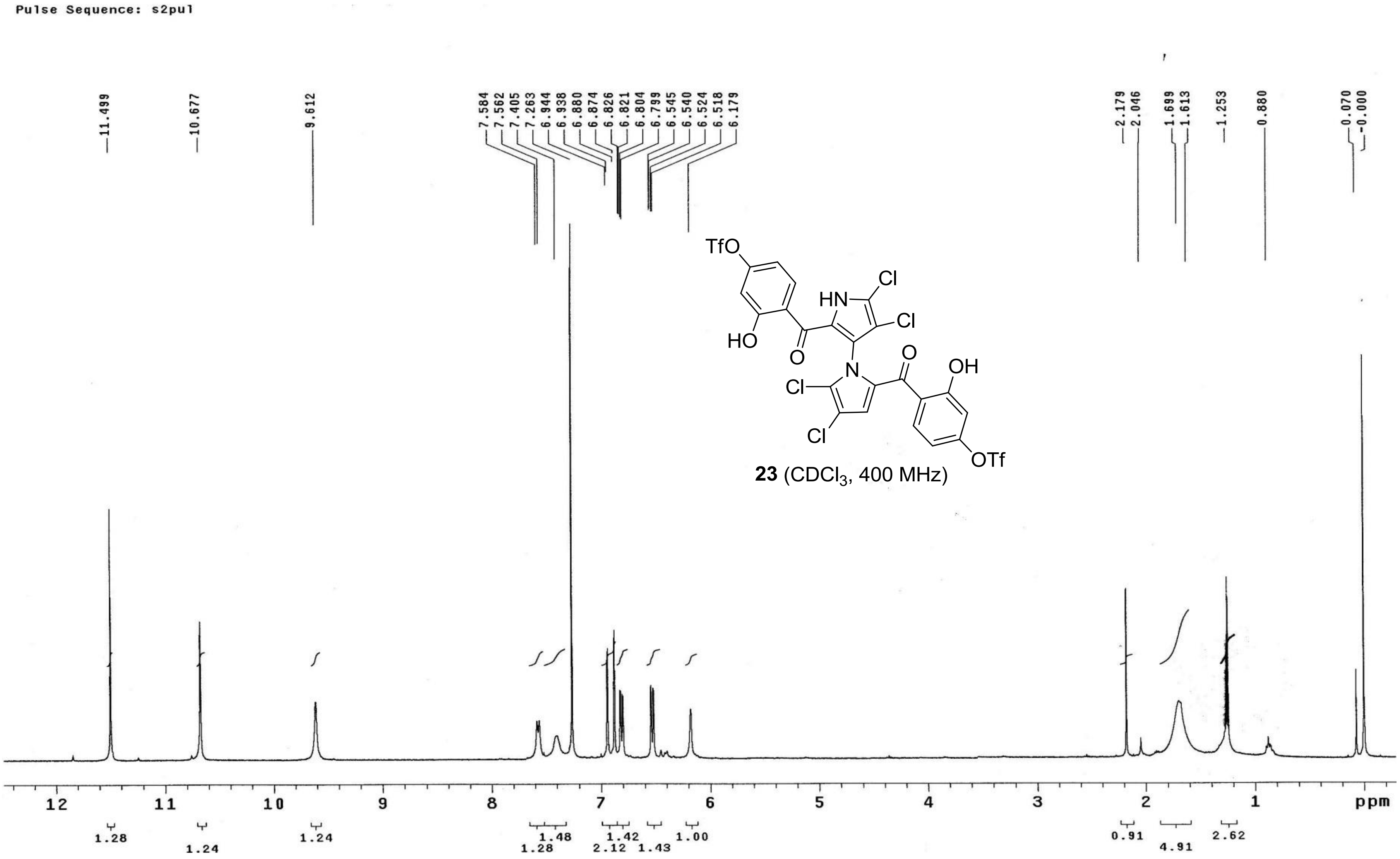

Figure S34. ^{13}C NMR spectrum of **23**.

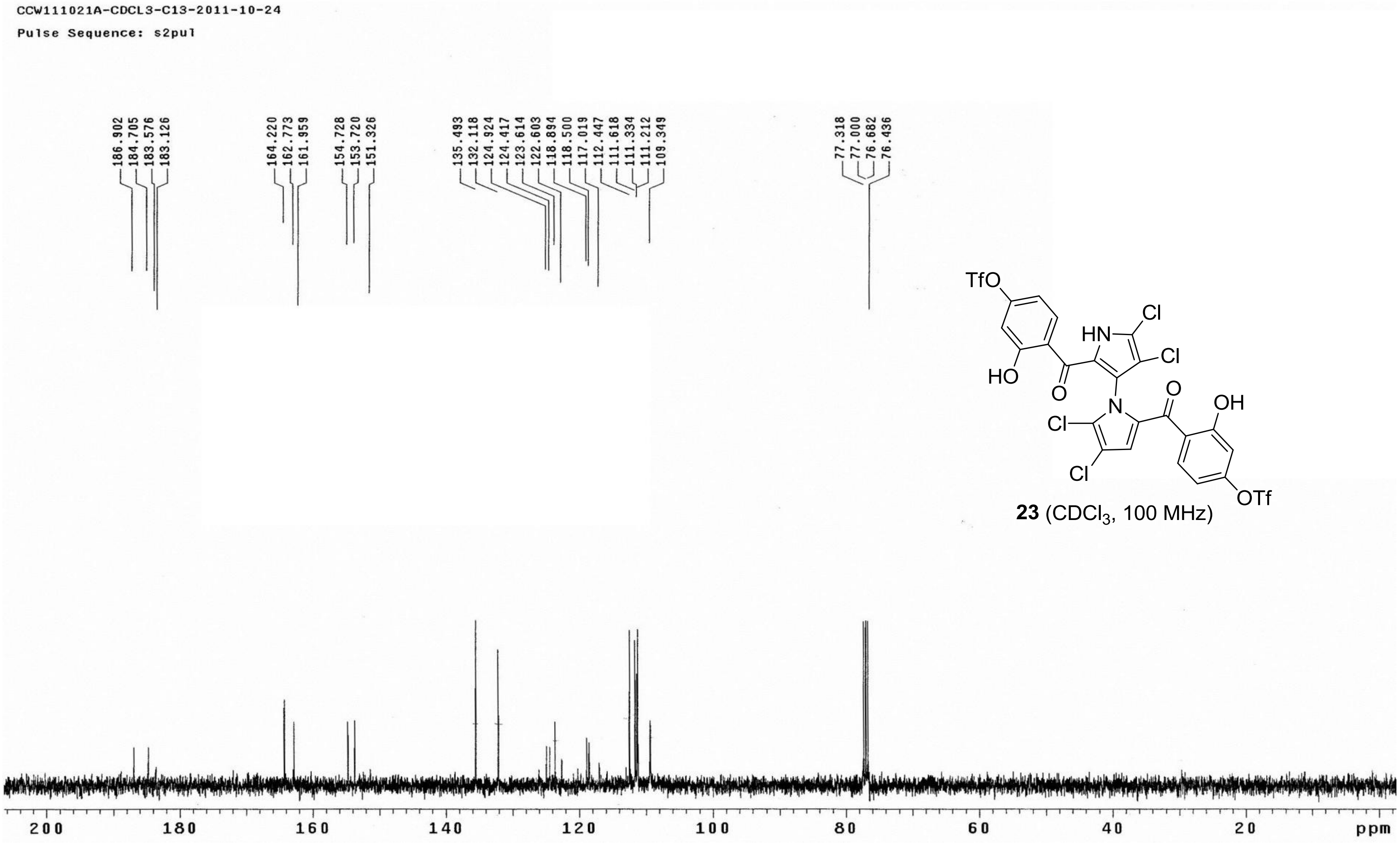

Figure S35. ^{1}H NMR spectrum of **24**.

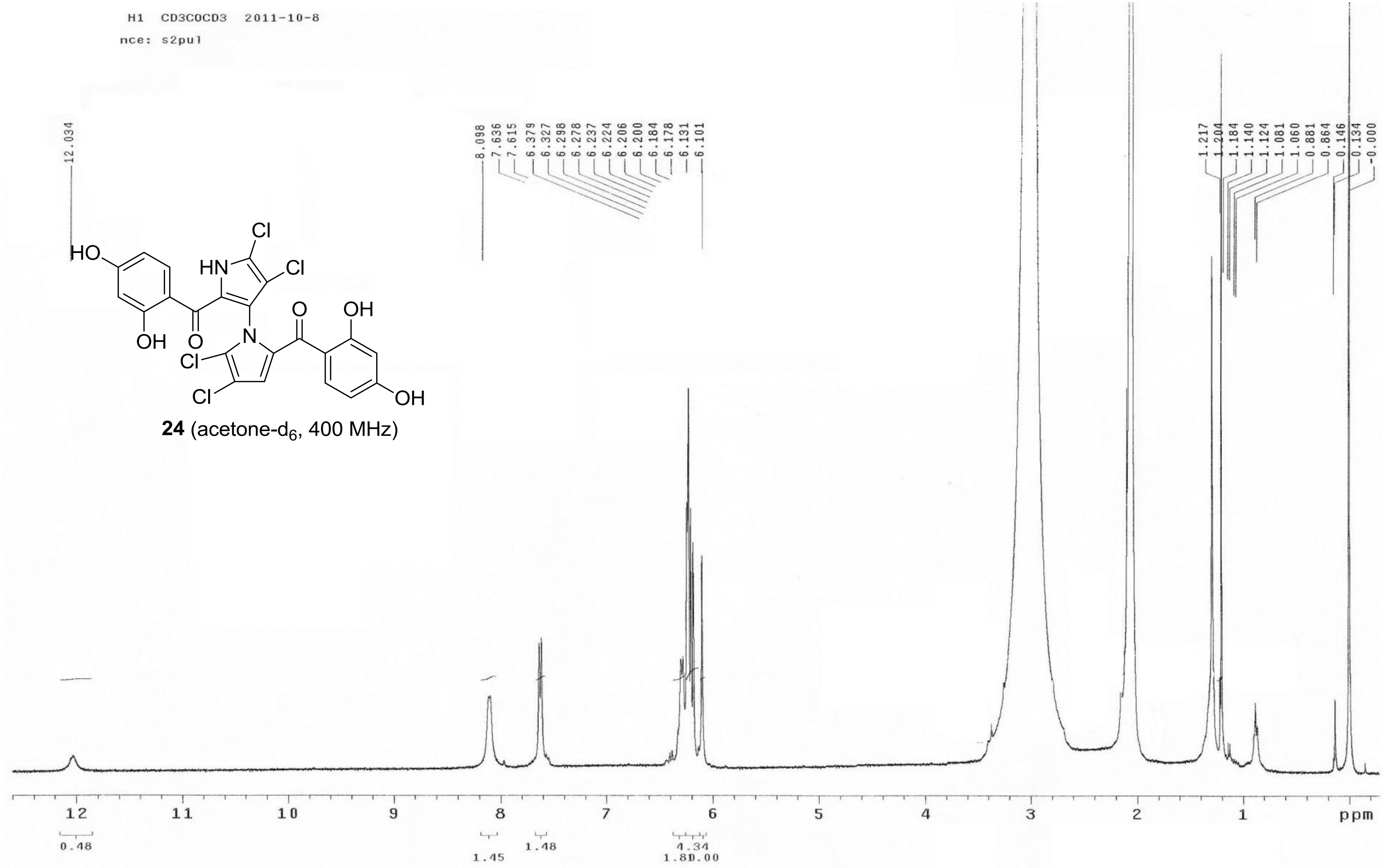

Figure S36. ^{13}C NMR spectrum of **24**.

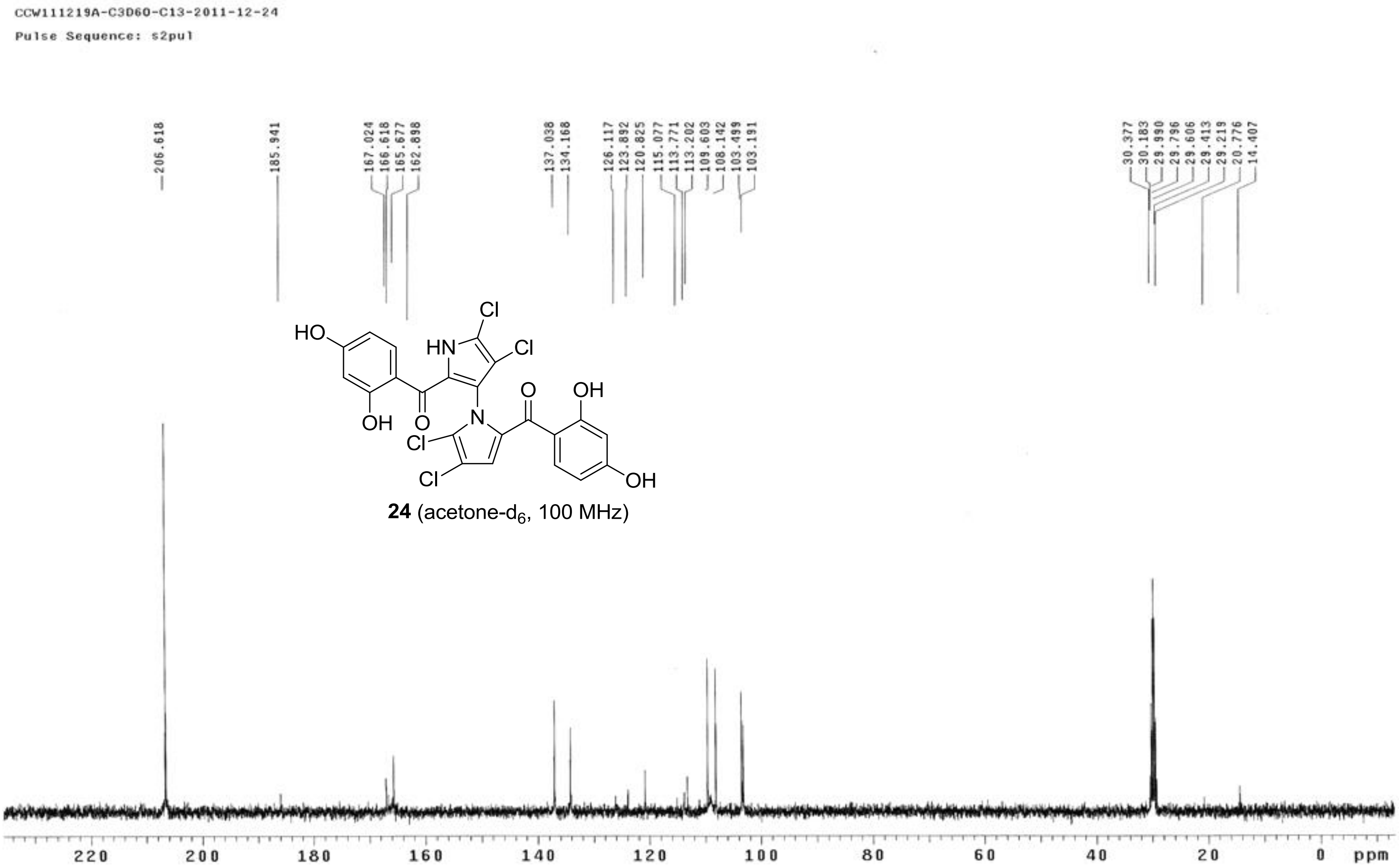

Figure S37. ^{1}H NMR spectrum of **25**.

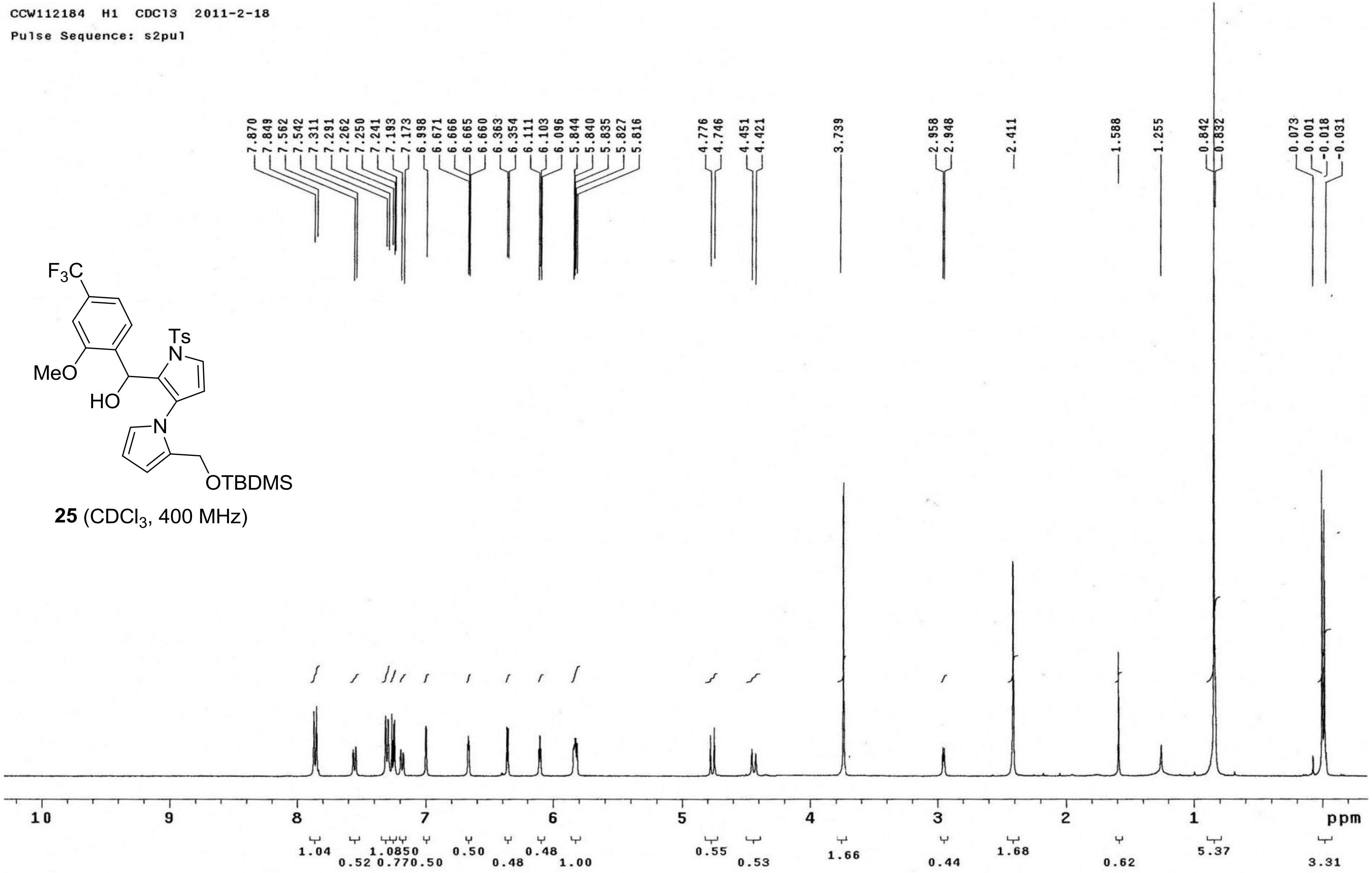

Figure S38. ^{13}C NMR spectrum of **25**.

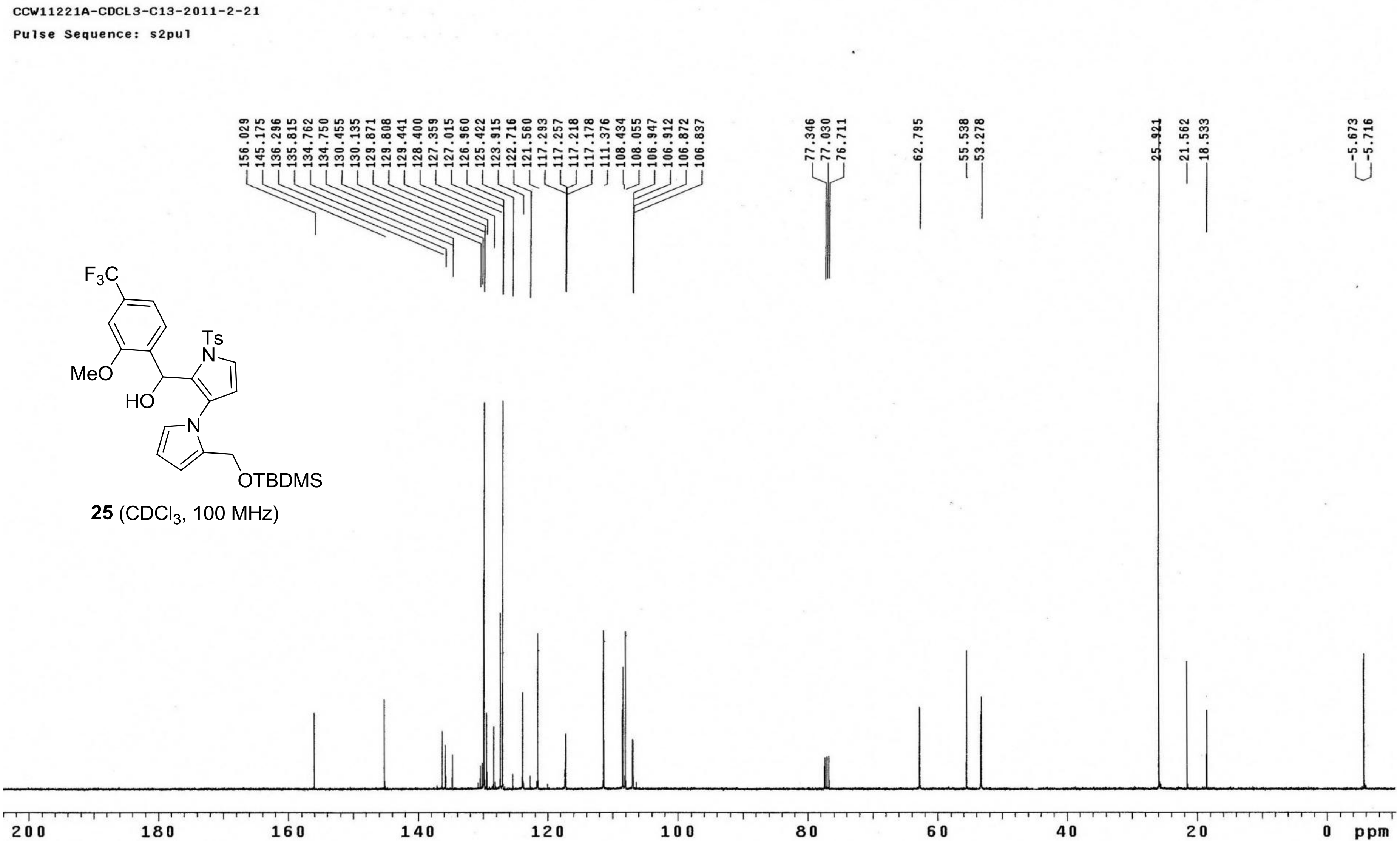

Figure S39. ^{1}H NMR spectrum of **26**.

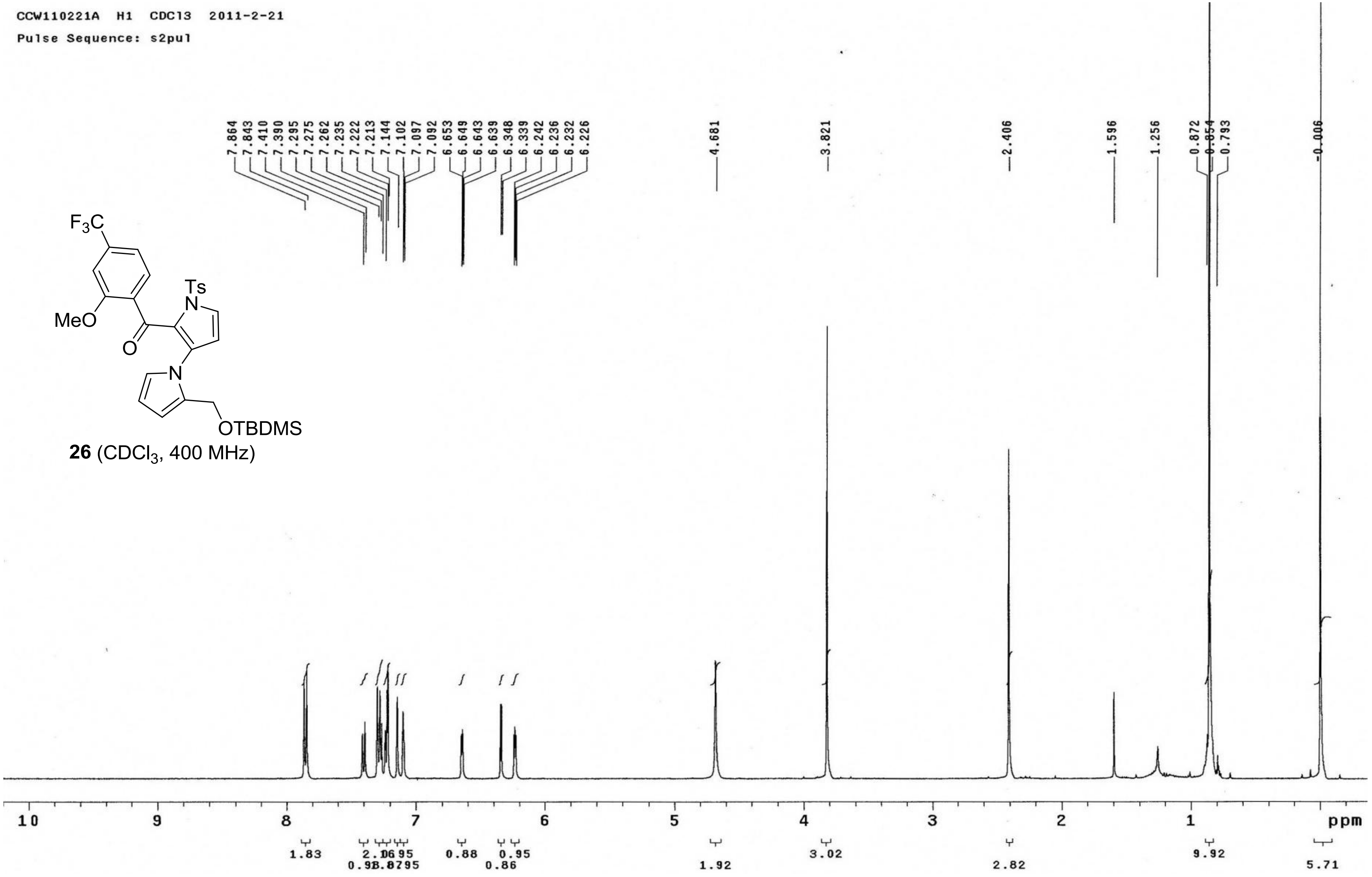

Figure S40. ^{13}C NMR spectrum of **26**.

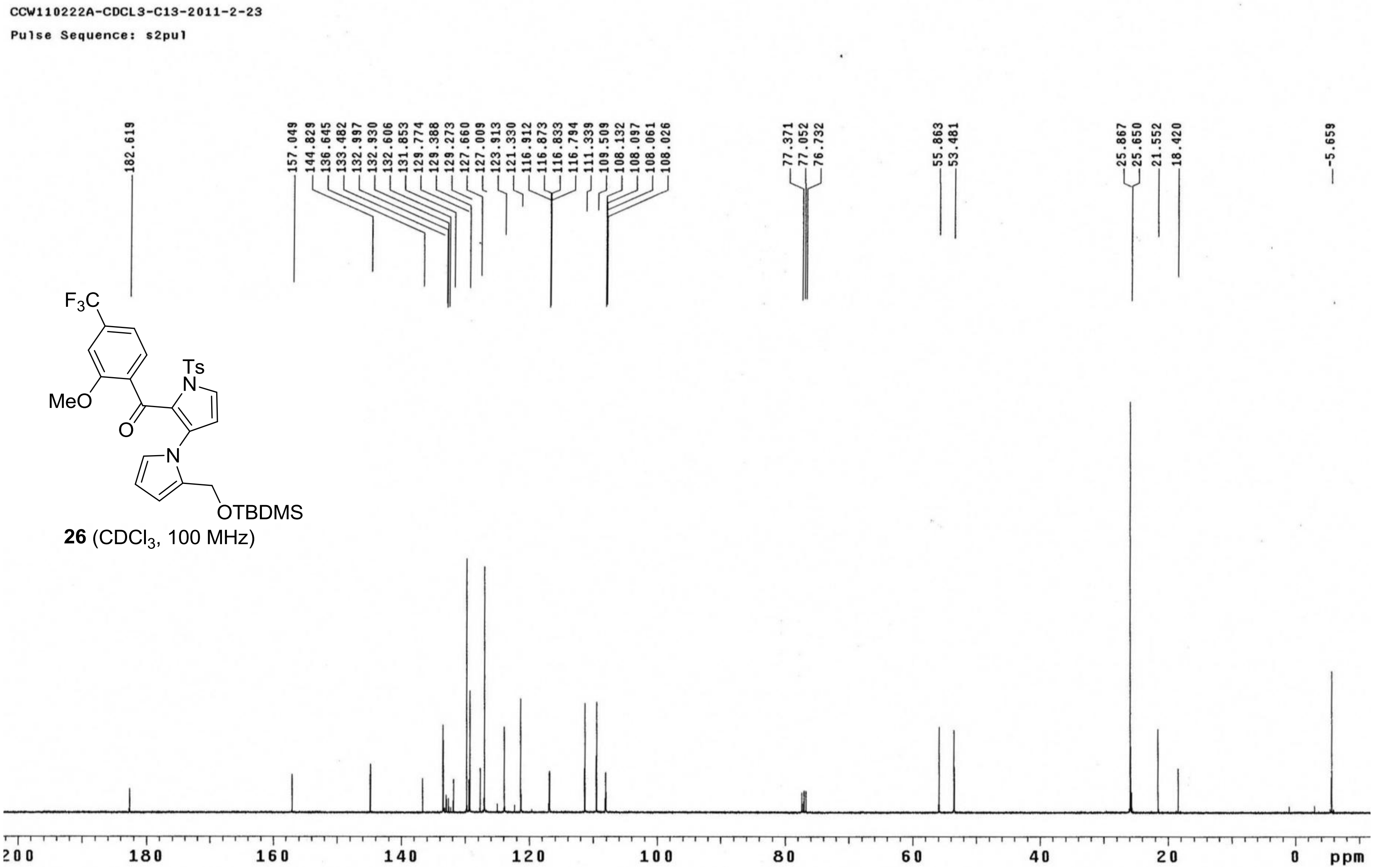

26 ($CDCl_3$, 100 MHz)

Figure S41. ^{1}H NMR spectrum of **27**.

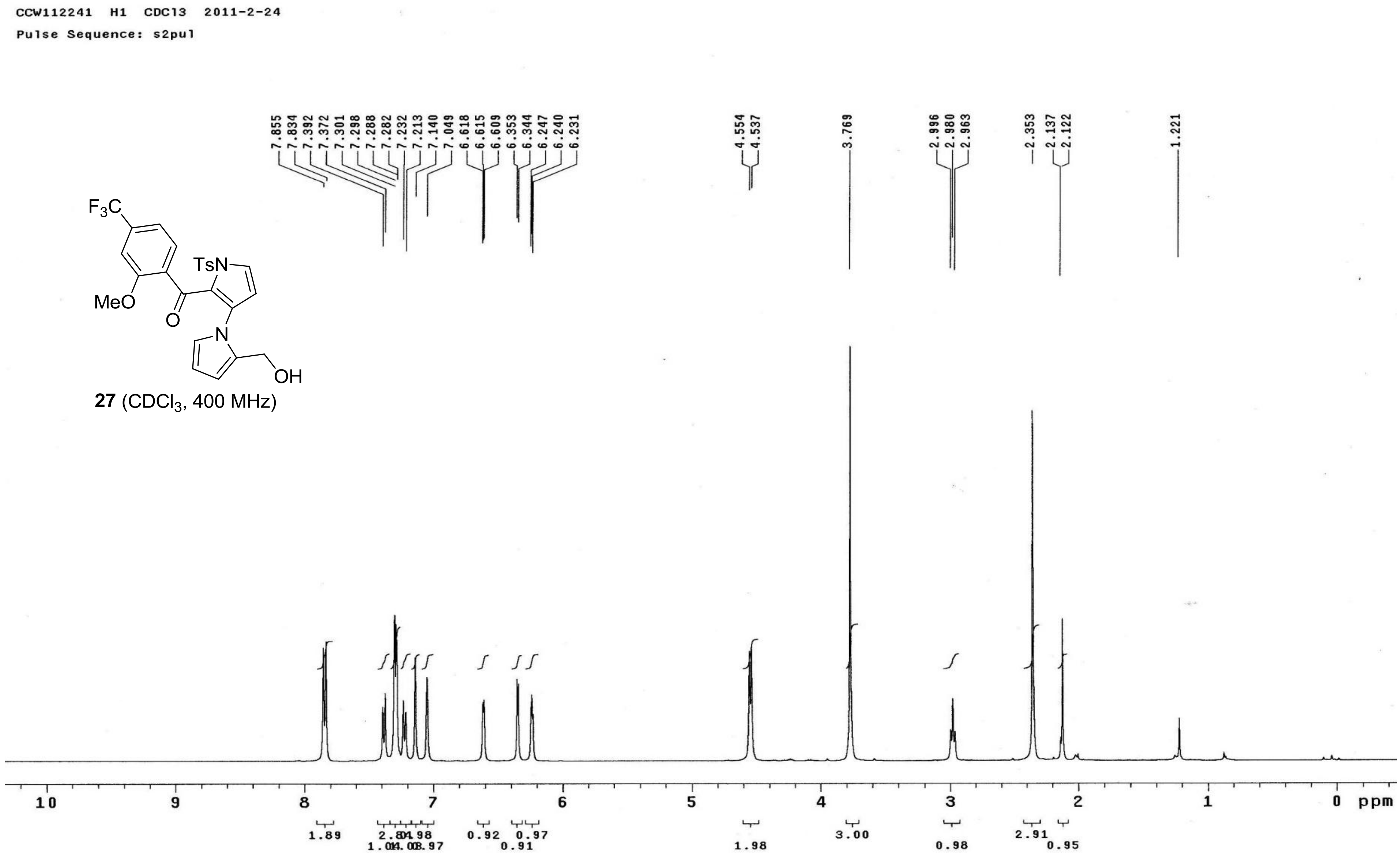

Figure S42. ^{13}C NMR spectrum of **27**.

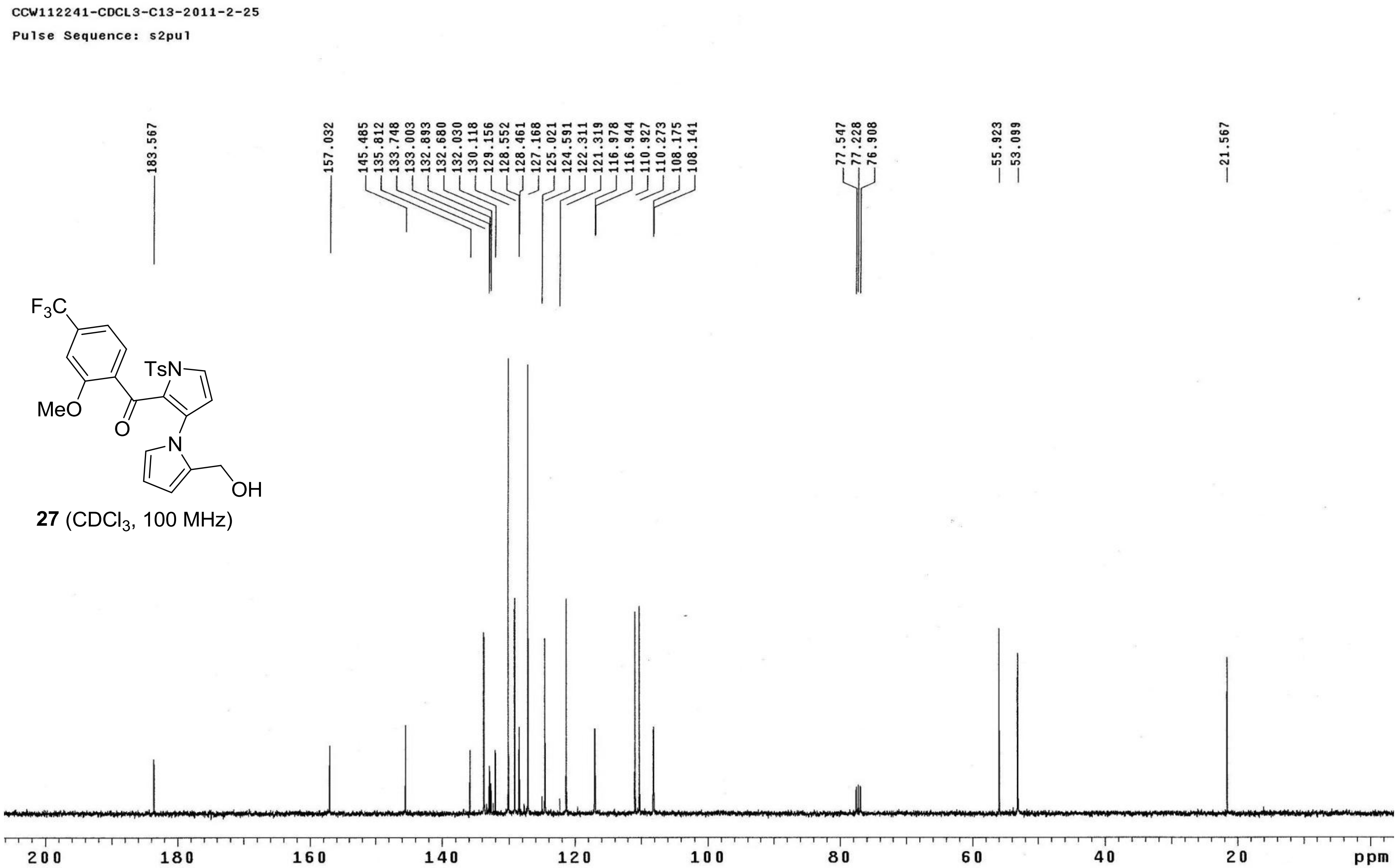

Figure S43. ^{1}H NMR spectrum of **28**.

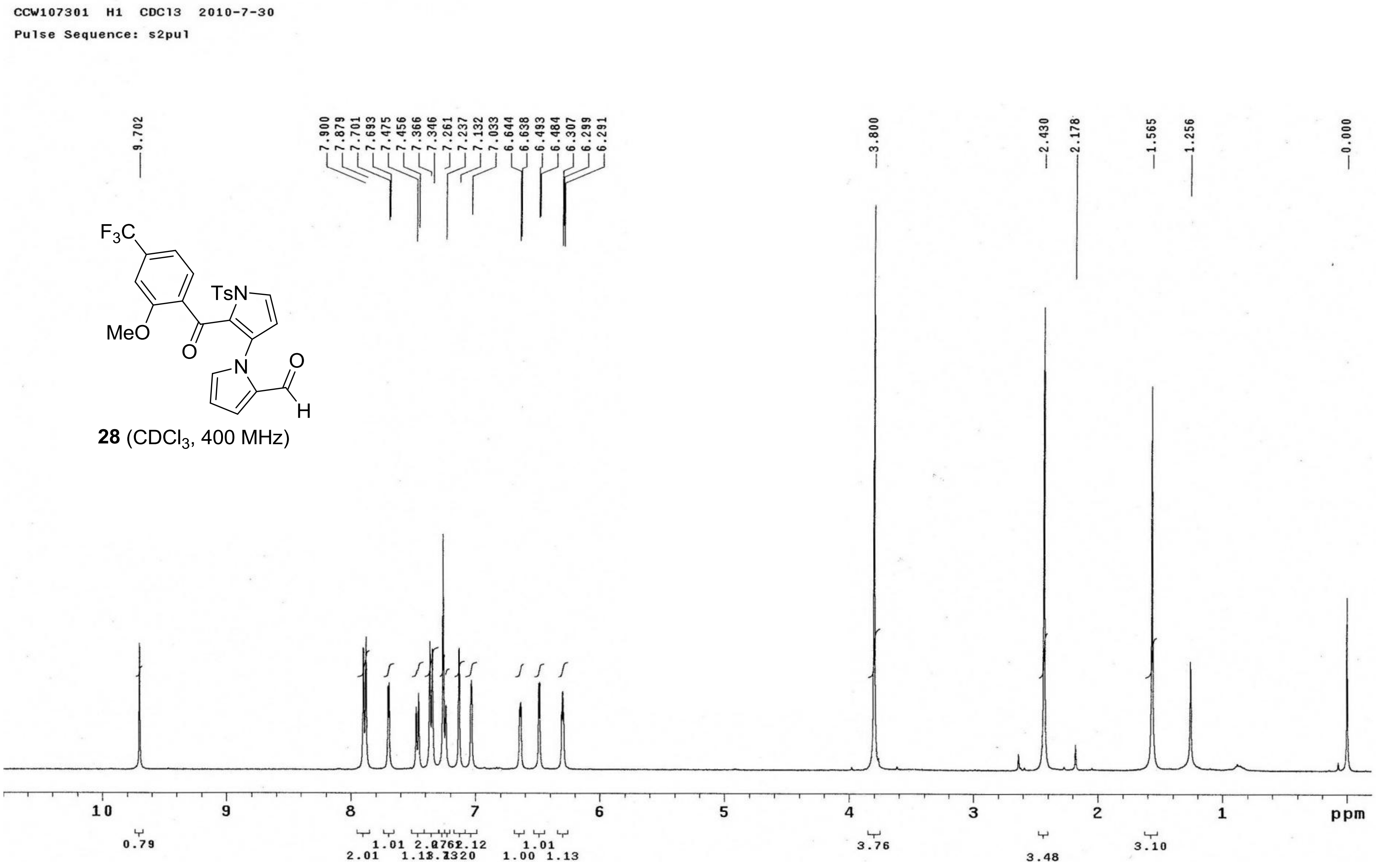

Figure S44. ^{13}C NMR spectrum of **28**.

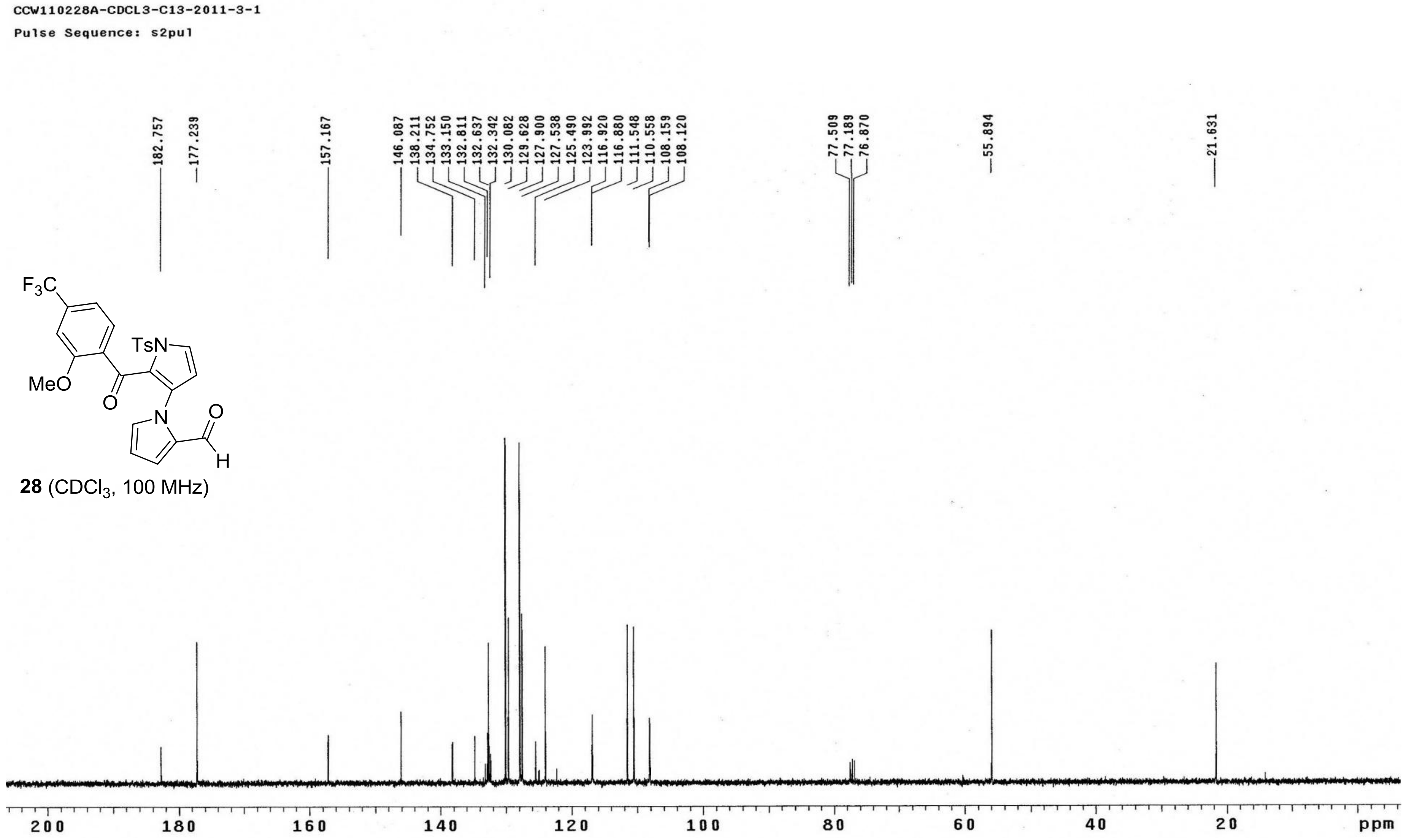

Figure S45. ^{1}H NMR spectrum of **29**.

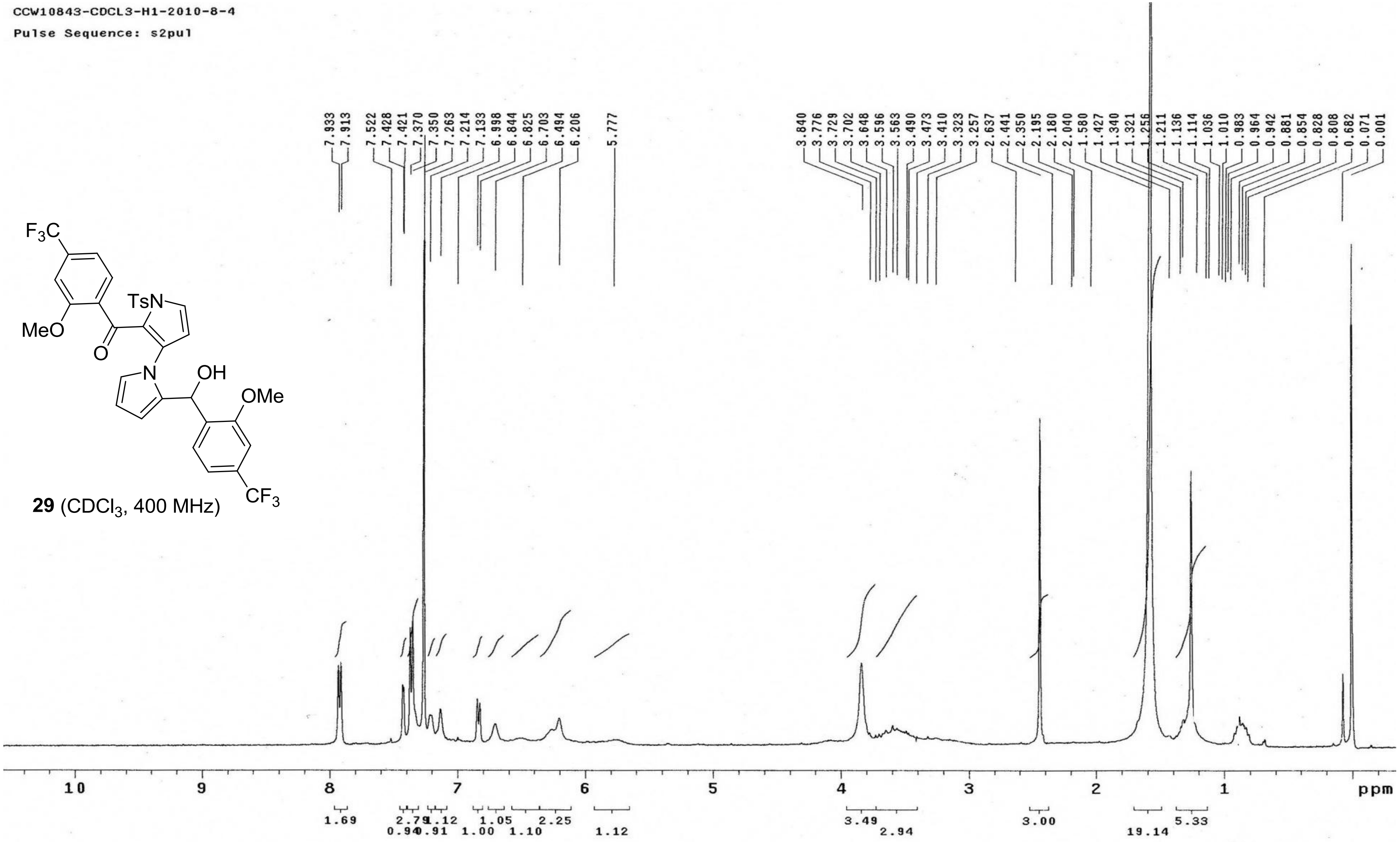

Figure S46. ^{13}C NMR spectrum of **29**.

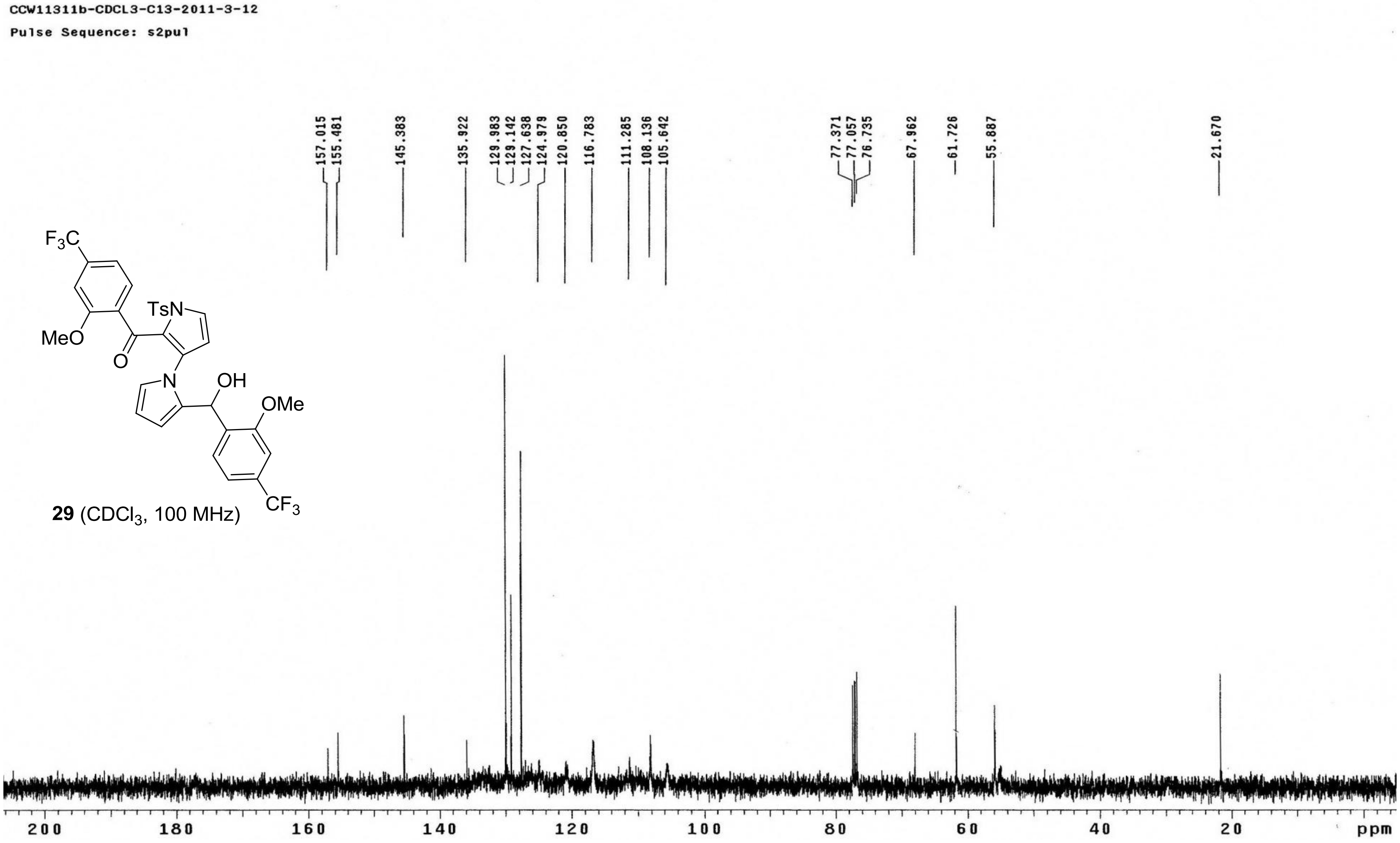

Figure S47. ^{1}H NMR spectrum of **29A**.

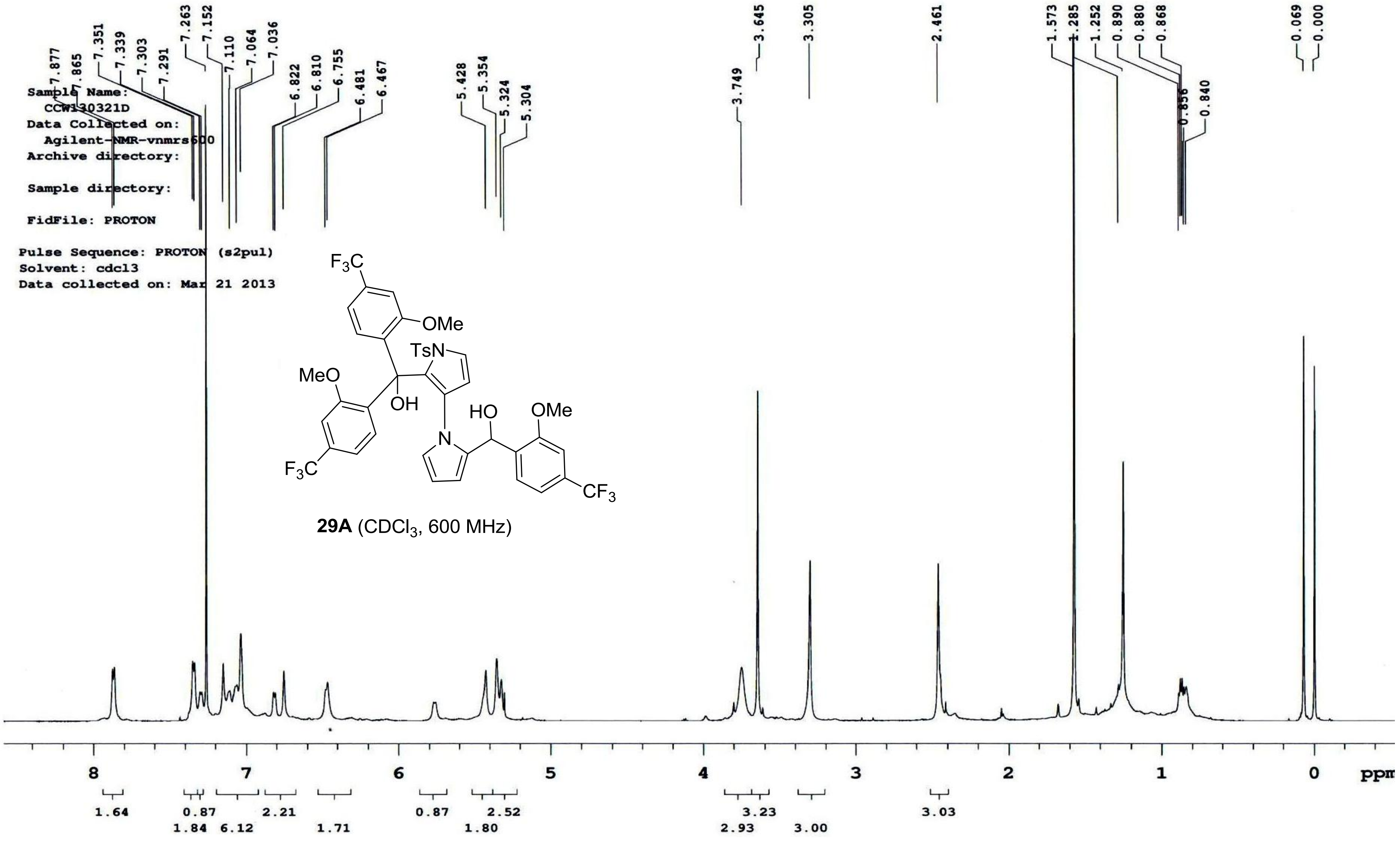

Figure S48. ^{13}C NMR spectrum of **29A**.

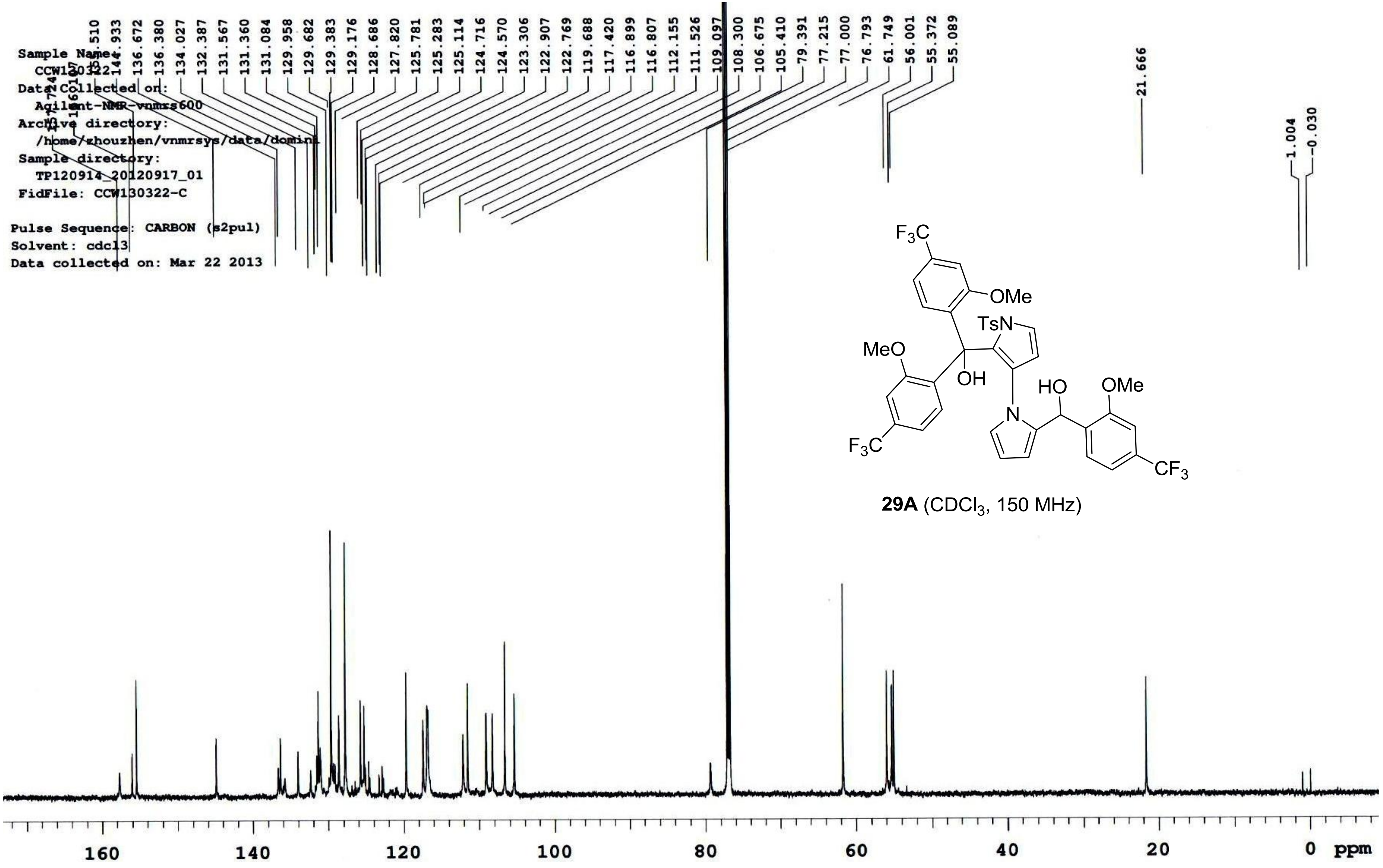

Figure S49. ^{1}H NMR spectrum of **30**.

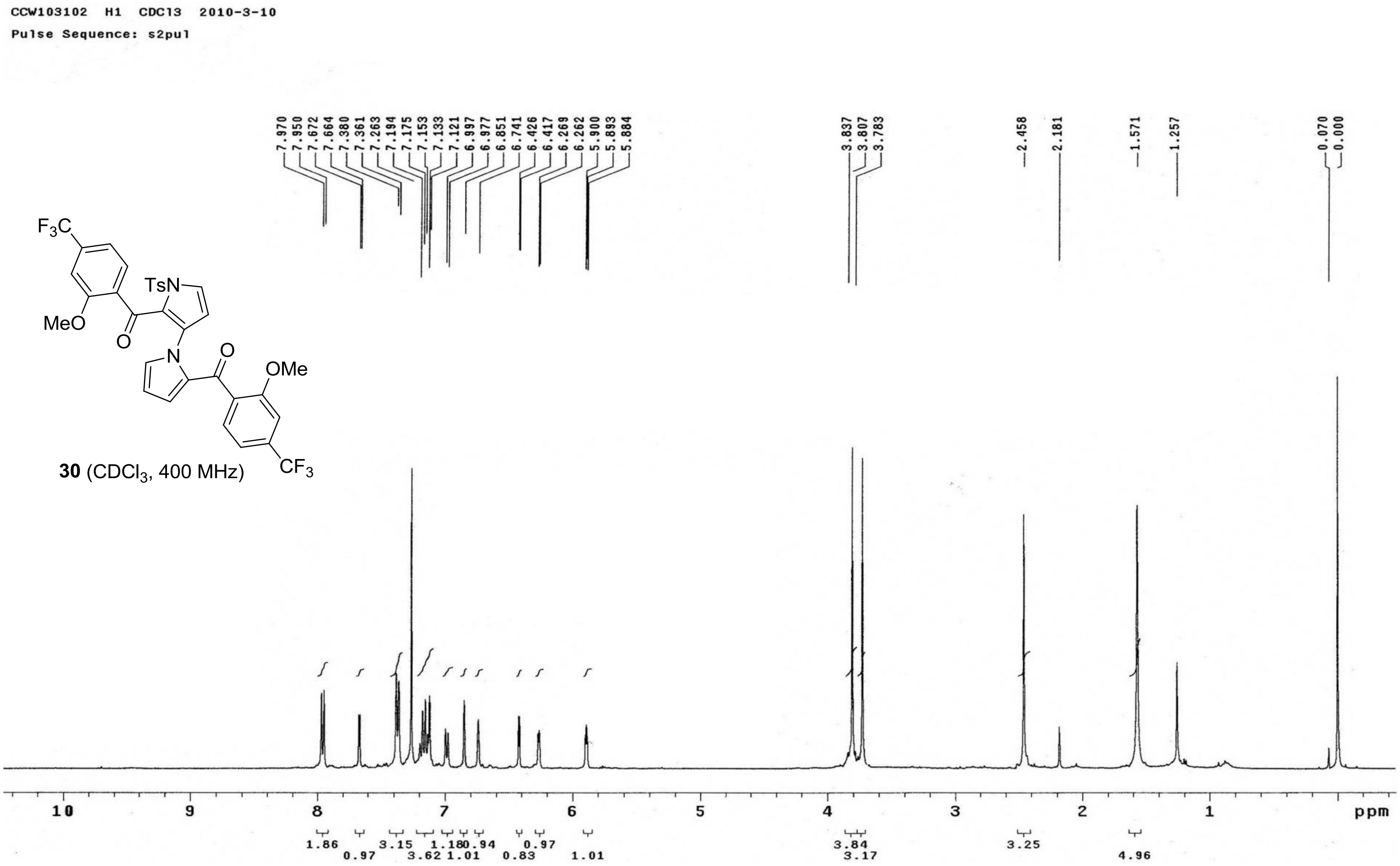

Figure S50. ^{13}C NMR spectrum of **30**.

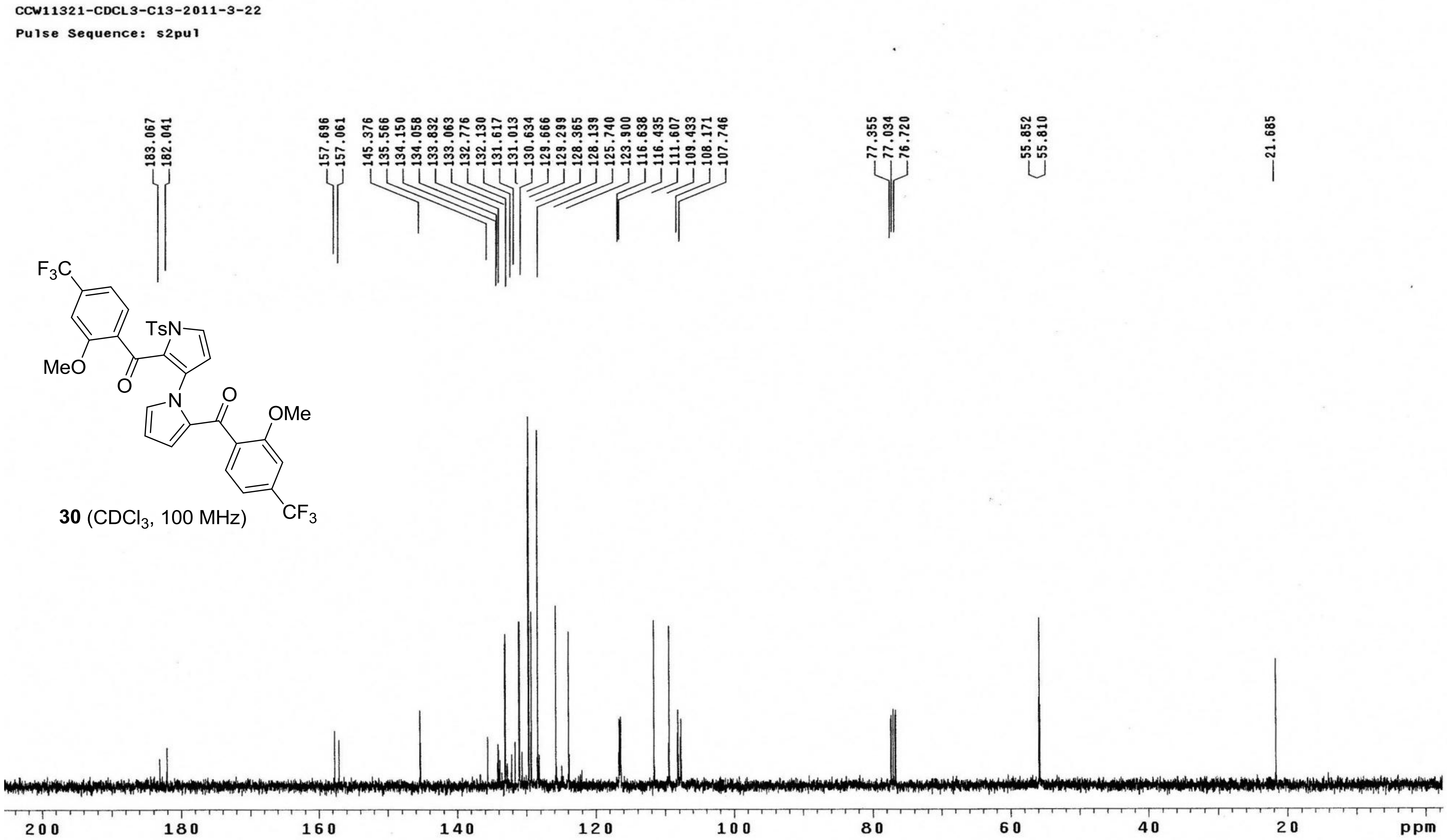

Figure S51. ^{1}H NMR spectrum of **31**.

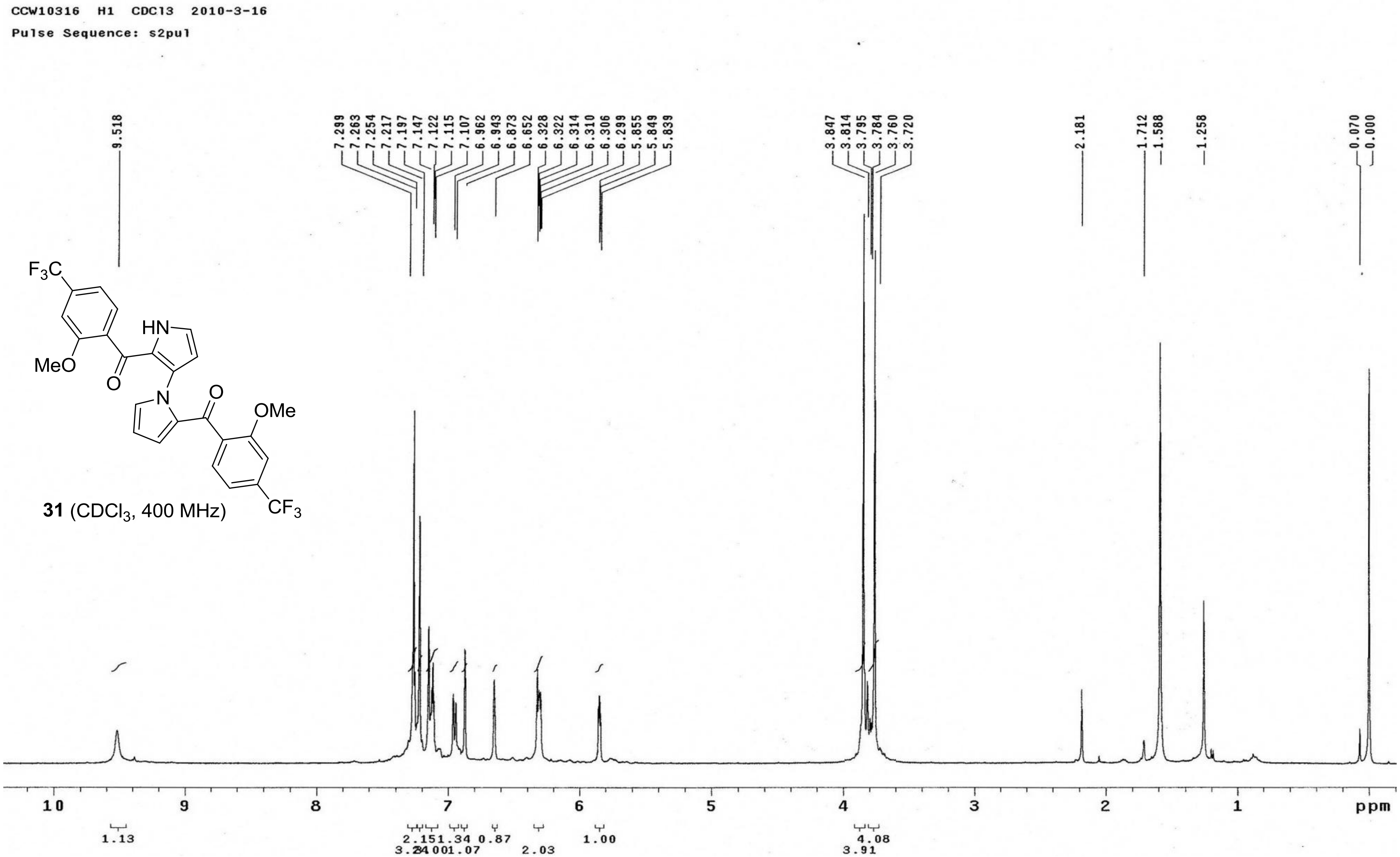

Figure S52. ^{13}C NMR spectrum of **31**.

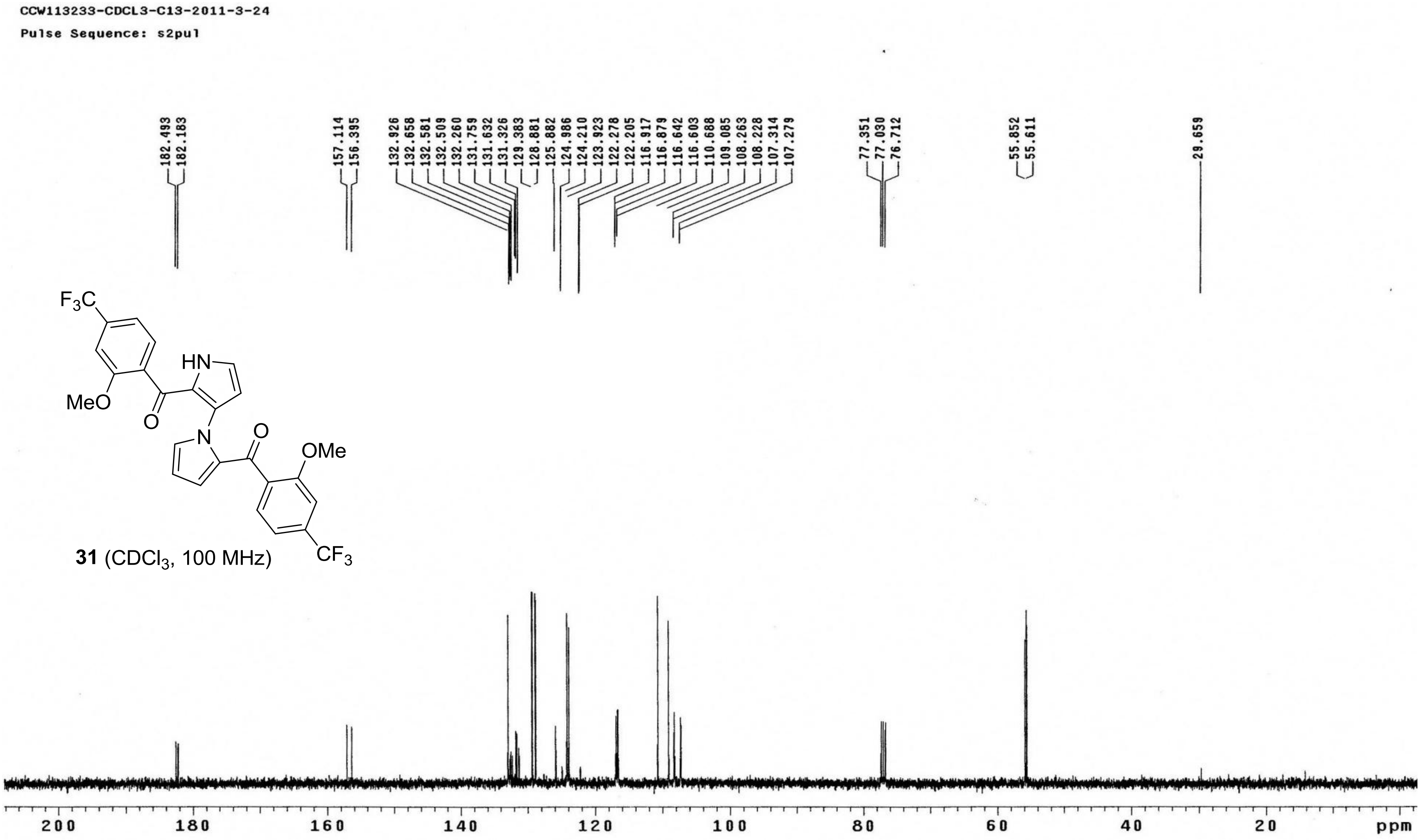

Figure S53. ^{1}H NMR spectrum of **32**.

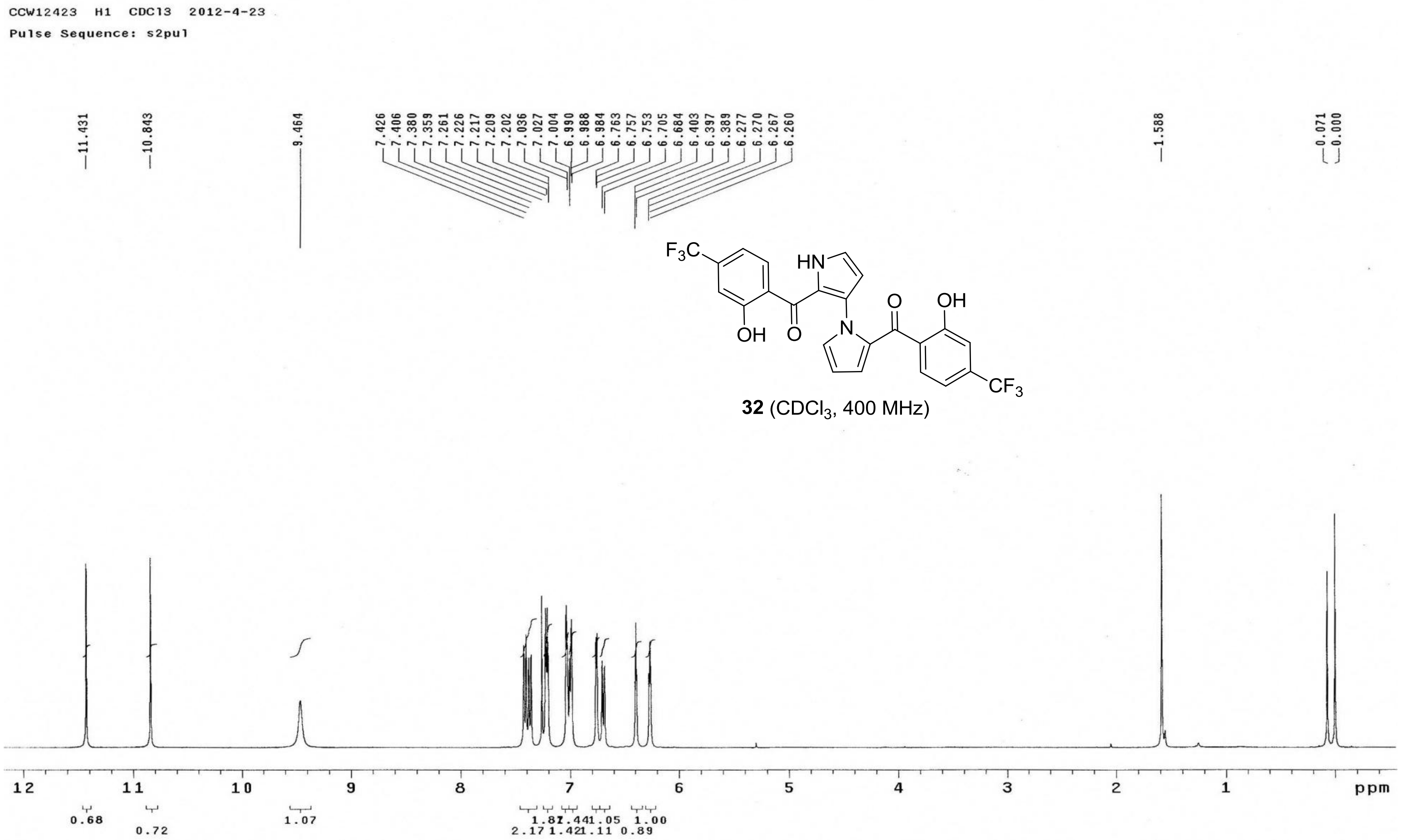

Figure S54. ^{13}C NMR spectrum of **32**.

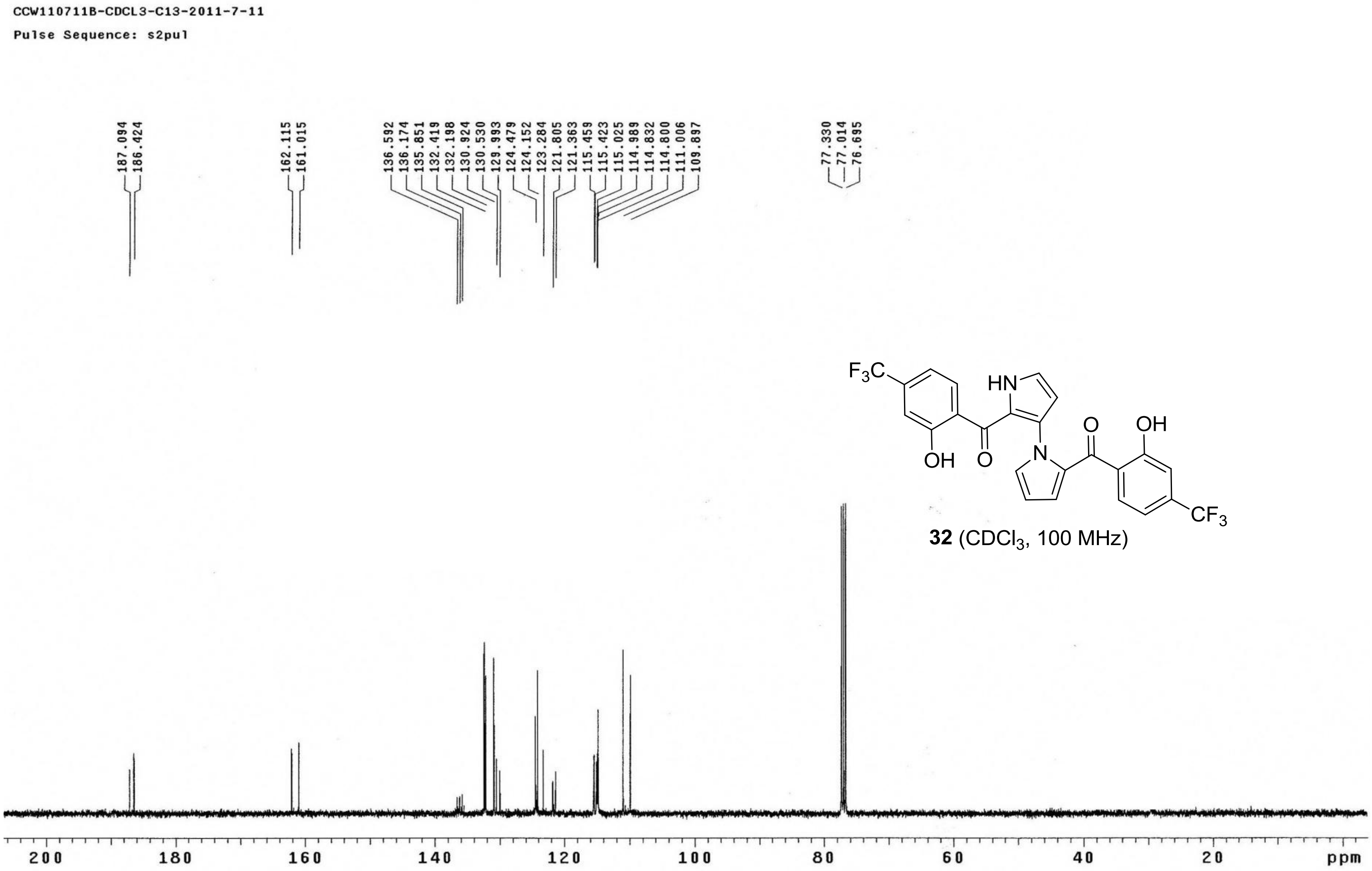

Figure S55. ^{1}H NMR spectrum of **33**.

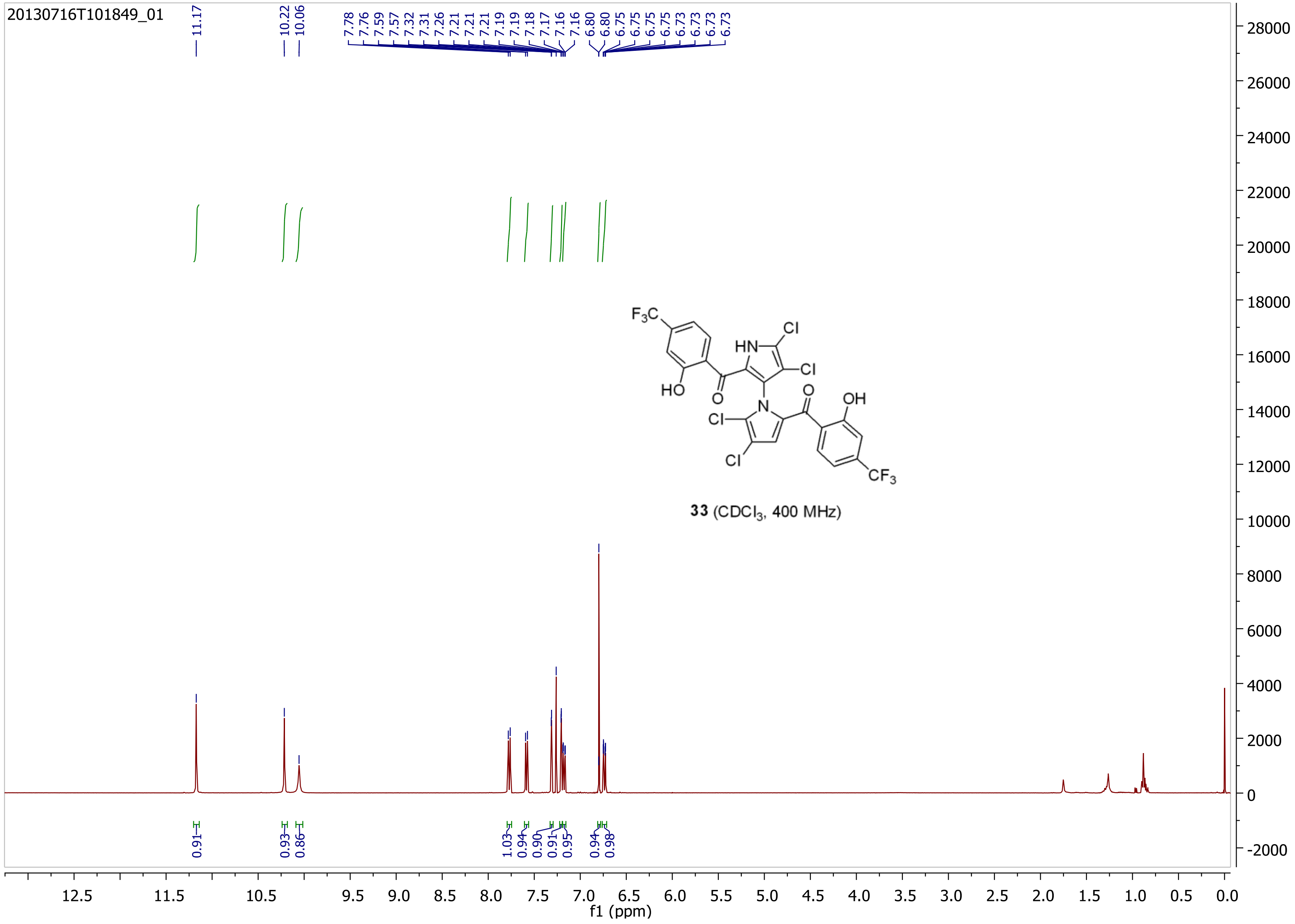

Figure S56. ^{13}C NMR spectrum of **33**.

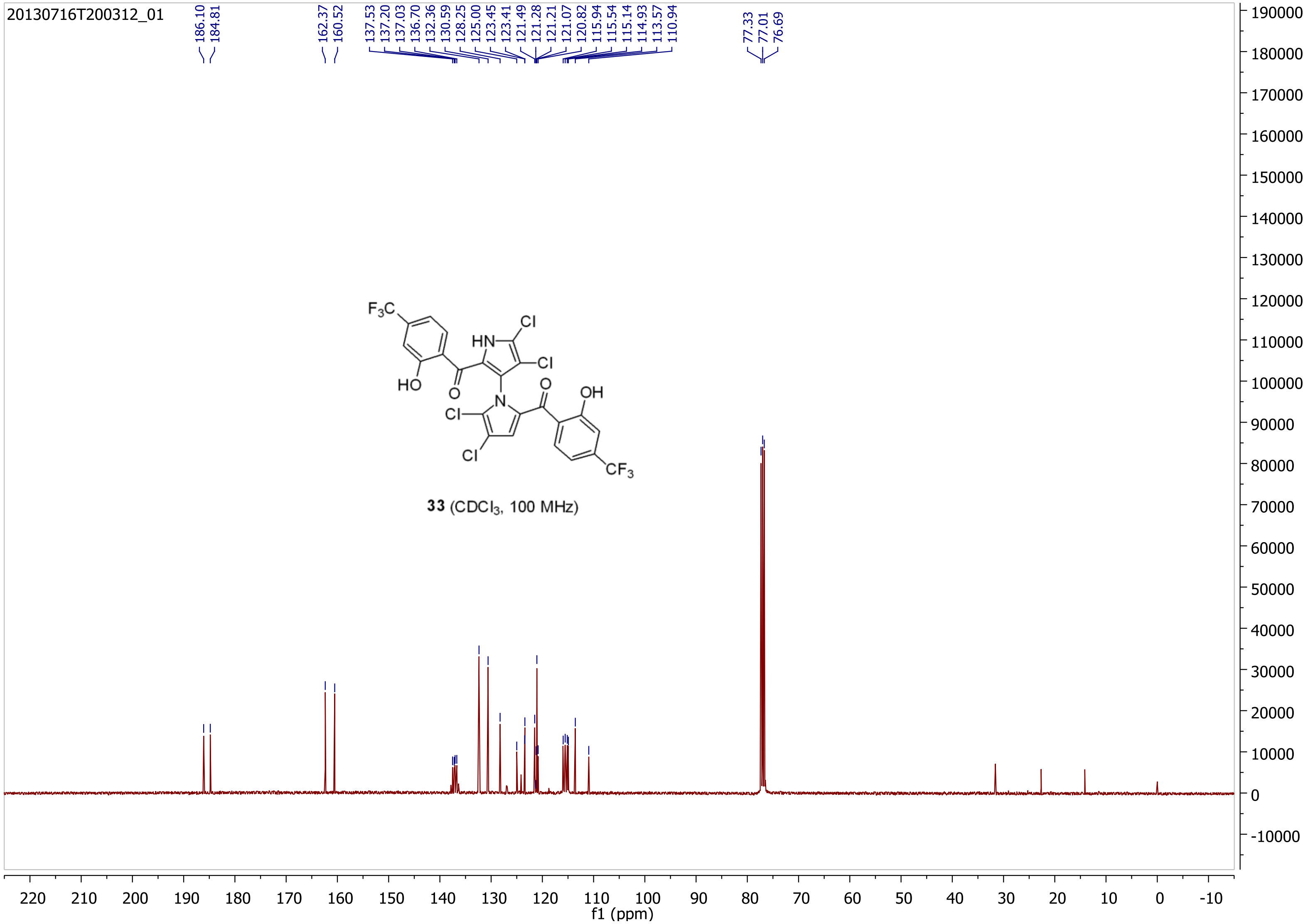

Figure S57. Purity of **24** by HPLC.

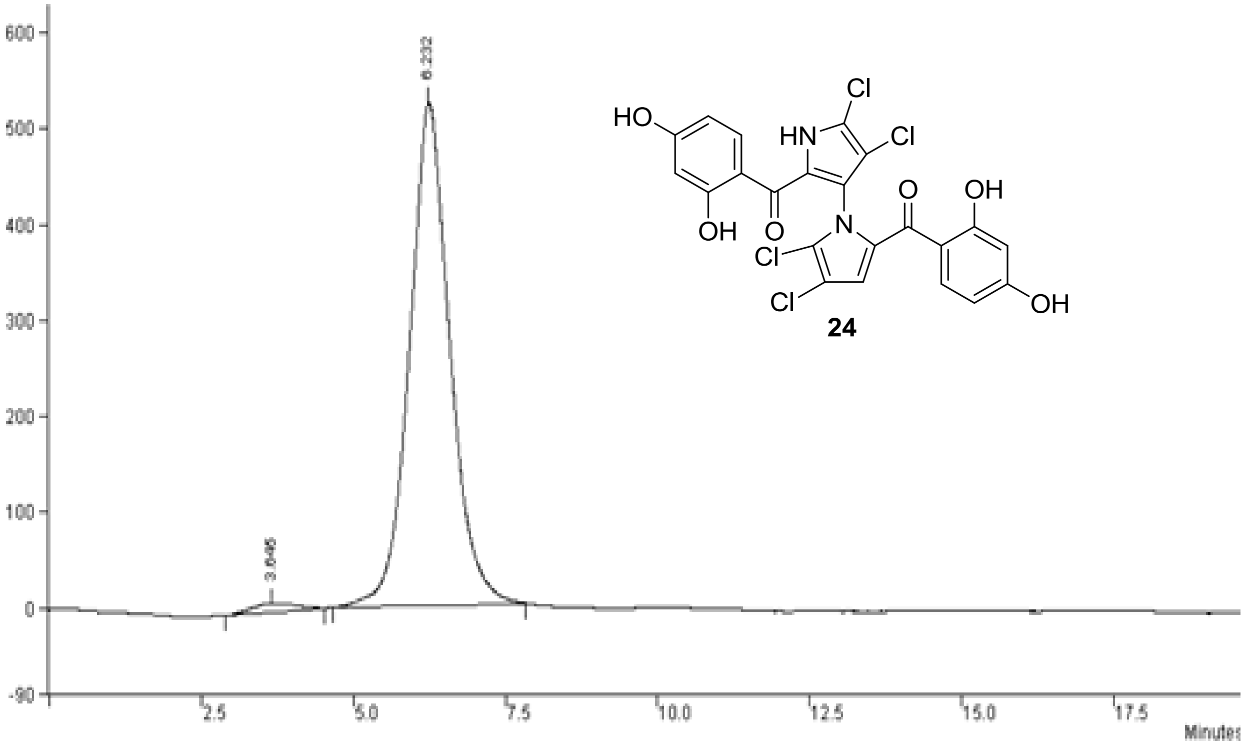

Peak No.	Peak Name	Result ()	Ret. Time (min)	Time Offset (min)	Area (counts)	Sep. Code	Width 1/2 (sec)
1		1.8104	3.645	0.000	4469985	BB	57.1
2		98.1896	6.232	0.000	242439904	BB	41.2
	Totals:	100.0000		0.000	246909889		

Figure S58. Purity of **33** by HPLC.

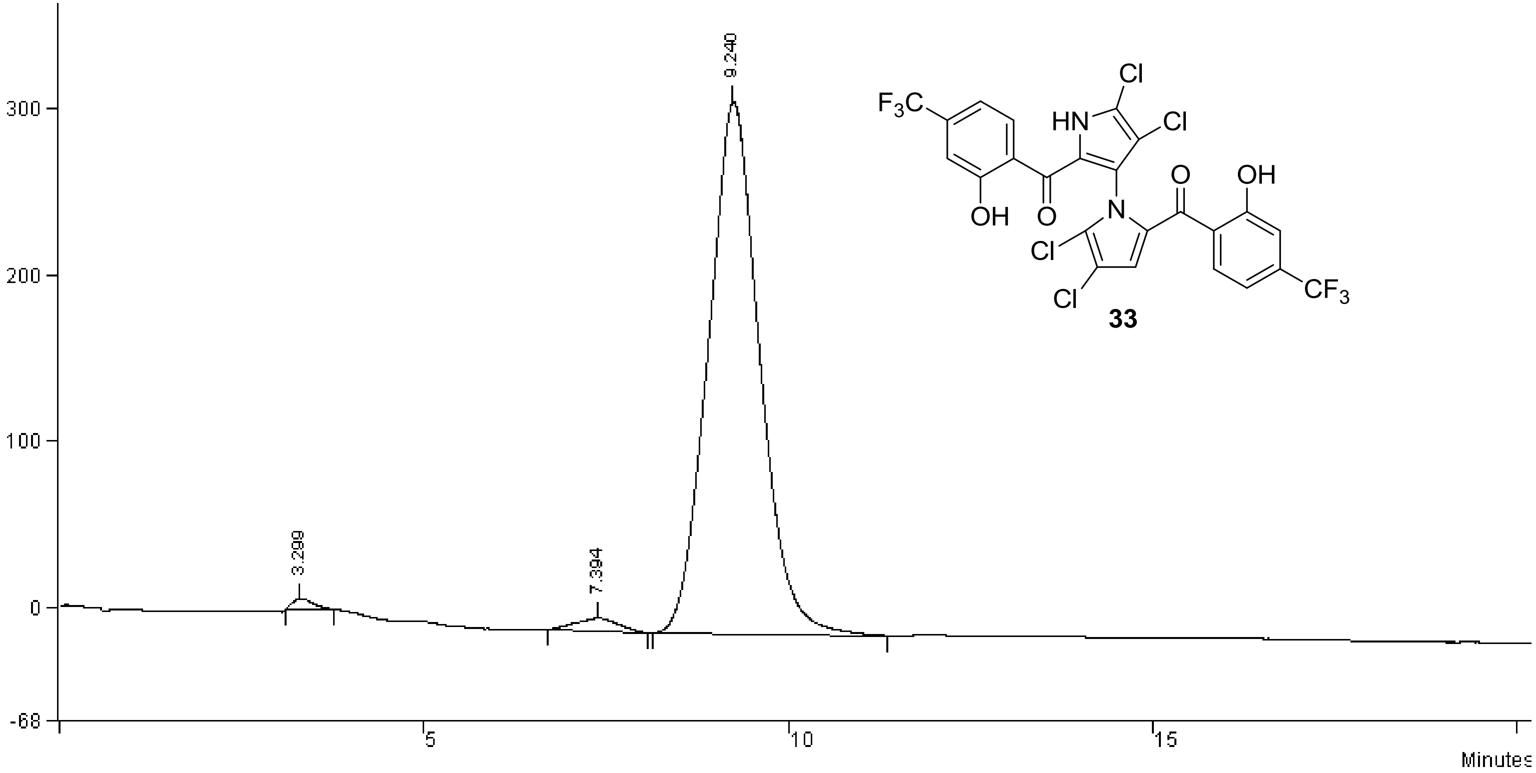

Peak No.	Peak Name	Result ()	Ret. Time (min)	Time Offset (min)	Area (counts)	Sep. Code	Width 1/2 (sec)
1		0.7681	3.299	0.000	1244523	BB	18.7
2		2.0153	7.394	0.000	3265186	BB	38.0
3		97.2166	9.240	0.000	157511904	BB	44.6
	Totals:	100.0000		0.000	162021613		
